# Context-dependent transcriptional regulations between signal transduction pathways

**DOI:** 10.1186/1471-2105-12-19

**Published:** 2011-01-13

**Authors:** Sohyun Hwang, Sangwoo Kim, Heesung Shin, Doheon Lee

**Affiliations:** 1 Department of Bio and Brain Engineering, KAIST, 373-1 Guseong-dong, Yuseong-gu, Deajeon, Republic of Korea; 2 Department of Mathematics, Inha University, 253 Yonghyun-dong, Nam-gu, Incheon, Republic of Korea

## Abstract

**Background:**

Cells coordinate their metabolism, proliferation, and cellular communication according to environmental cues through signal transduction. Because signal transduction has a primary role in cellular processes, many experimental techniques and approaches have emerged to discover the molecular components and dynamics that are dependent on cellular contexts. However, omics approaches based on genome-wide expression analysis data comparing one differing condition (e.g. complex disease patients and normal subjects) did not investigate the dynamics and inter-pathway cross-communication that are dependent on cellular contexts. Therefore, we introduce a new computational omics approach for discovering signal transduction pathways regulated by transcription and transcriptional regulations between pathways in signaling networks that are dependent on cellular contexts, especially focusing on a transcription-mediated mechanism of inter-pathway cross-communication.

**Results:**

Applied to dendritic cells treated with lipopolysaccharide, our analysis well depicted how dendritic cells respond to the treatment through transcriptional regulations between signal transduction pathways in dendritic cell maturation and T cell activation.

**Conclusions:**

Our new approach helps to understand the underlying biological phenomenon of expression data (e.g. complex diseases such as cancer) by providing a graphical network which shows transcriptional regulations between signal transduction pathways. The software programs are available upon request.

## Background

Signal transduction is the primary process by which cells coordinate their metabolism, proliferation, and cellular communication according to environmental signals such as hormones, nutrients, and other chemical stimuli. Cells sense environmental signals by receptor proteins which convert the signals into various responses through signal transduction that are dependent on cellular contexts such as signals, receptor proteins that cells possess, and intracellular machinery by which cells integrate and interpret the signals [1]. For example, the JAK-STAT signal transduction pathway, which provides one of the most direct routes from cell-surface receptors to a nucleus, is activated by more than 30 cytokines of soluble mediators in cell communication. The cellular responses are different according to their cytokines even though they are stimulated by the same JAK-STAT signal transduction pathway [1].

As well as for various responses stimulated by signal transduction pathways or signaling pathways, recent articles have presented abundant evidence for inter-pathway cross-communication according to cellular contexts [2-4]. Cytokine signaling which is critical in immune system regulates functions of other signaling pathways either by transcription-mediated consequences of cytokine signaling or by transcription-independent mechanisms [2]. As an example of transcription-mediated mechanisms, interferon gamma activates signal transduction pathways of toll-like receptors (TLRs) by inducing expression of TLRs [5]. An example of transcription-independent mechanisms, Bezbradica and Medzhitove [2] suggested that lateral interactions between cytokine receptors and other cellular receptors may explain how different cells induce their cell-type specific responses with a highly limited set of janus kinase (JAK) and signal transducer and activator of transcription (STAT) signaling proteins.

Among the two mechanisms of cross-communication between signaling pathways according to cellular contexts, we focus on the transcription-mediated mechanism that can be inferred by integrating omics data as well as genome-wide expression data. Various methods analyzing expression data by integrating omics data have been employed to infer sub-networks perturbed at cellular context with protein-protein interaction (PPI) data[6-10]. Ideker et al. [8] first proposed to identify sub-networks by devising an adequate scoring function on PPI networks based on the significant changes in gene expression. By adapting the scoring concept, many similar approaches have improved the search algorithms [10] or scoring functions [6,9]. However, previous approaches that inferred sub-networks did not provide transcription-mediated communication between signaling pathways, because they could not identify signaling pathways regulated by transcription at cellular contexts and PPI data have the noise problem [11].

Therefore, we propose a new computational omics approach for discovering signaling pathways regulated by transcription, Transcription-Regulating Signaling Pathways (TRS Pathways) and transcriptional regulations between pathways in Transcription-Regulating Signaling Networks (TRS Networks) that are dependent on cellular contexts. In this approach, cellular contexts are restricted to the experimental condition of expression data. TRS Pathways are signaling pathways of which some proteins are regulated by transcription according to the context of expression data. Signaling pathways are chains of proteins relaying a signal from ligands or transmembrane proteins to transcription factors, or some proteins whose roles are clearly known such as caspase3 [1,12]. Proteins regulated by transcription represent the mRNA expression levels of proteins at the context which are significantly changed. TRS Networks are sub-networks which result from transcriptional relation between TRS Pathways. Applied to dendritic cells treated with lipopolysaccharide, we found several biological facts and transcriptional regulations as examples of inter-pathway cross-communication, related to dendritic cell maturation and T cell activation.

## Methods

Our system comprises three major steps: (1) constructing a human omics network from PPIs and Protein-DNA (PD) interactions; (2) identifying TRS Pathways by two strategies: three constraints to reduce the search space for TRS Pathways and designing a scoring function for TRS Pathways; (3) identifying the TRS Networks by a search algorithm. After explaining these three major steps of the system, we briefly explain the scoring function for TRS Networks, designed to compare with sub-networks inferred by previous methods, expression data analyses and kinase reaction annotations.

### Constructing a human omics network

**Figure 1:**
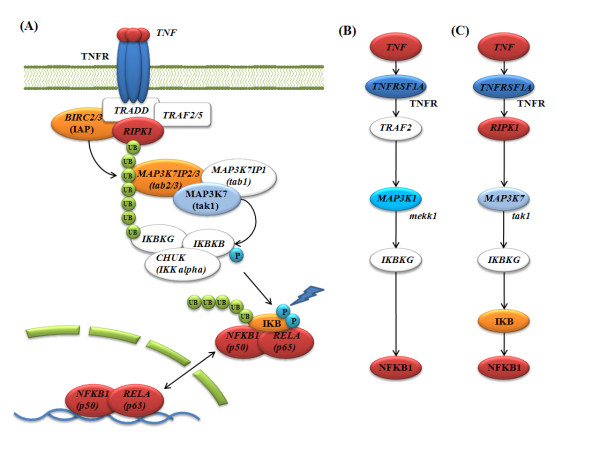
**TRS Pathways relaying of a signal from TNF to NFKB**. Each node represents a protein whose name follows the gene name. The color indicates its expression levels (fold change): red (≥ 3), orange (≥ 2 and < 3), yellow (≥ 1 and < 2), light yellow (≥ 0.5 and <1), white (> -0.5 and < 0.5), light cyan (> -1 and ≤ -0.5), cyan (> -2 and ≤ -1) and navy blue (> -3 and ≤ -2) and blue (≤ -3). The small UB and P circles represent ubiquitins and phosphates. (A) One of the known pathways from TNF to NFKB [35]. (B) In the KEGG TRS Pathway relaying a signal from TNF to NFKB, MAP3K1 (mekk1) is used as IKB kinase kinase (IKKK). (C) The omics network TRS Pathway seems to select each protein from its corresponding protein complex of (A) to make a linear path. The expression condition is 2h after the LPS treatment.

A human omics network is a directed graph comprising Protein-Protein Interactions (PPIs) and Protein-DNA (PD) interactions. The interactions were collected from three types of data: PPI, PD, and KEGG database. Firstly, PPI data were from four public databases; BioGRID version 2.0.26 [13], IntAct [14], HPRD Release 7 [15], and MINT [16]. Secondly, the PD interaction data were from three public data; the results of ORFeome-based analysis [17], bZIPDB [18], and MSigDB [19]. Thirdly, we added the PPI and PD interaction data of KEGG [20] into the above integrated PPI and PD data, because they missed many signaling PPI interactions in the KEGG database (Figure S1). Even though adding KEGG interactions into the omics network can cause a circularity problem of the results, we proved that it is a useful and necessary method to find new pathways that do not exist in KEGG pathways from searching the omics network (Figure 1). These interaction data, collected from the eight databases, were integrated based on the Entrez gene information at NCBI providing abundant external references to other databases [21]. The integrated omics network comprises 10,960 nodes and 113,220 edges. Since PPI interactions from four public PPI database have no directional information, one PPI was transformed into two PPIs having opposite directions.

### The two strategies for identifying TRS Pathways

Because finding the highest-scoring connected sub-networks like TRS Pathways in the entire interaction network is a NP-hard problem [8], we adopted two strategies. The first strategy is to reduce the search space for TRS Pathways by three constraints. The second is to find the pathways only with top scores by defining the scoring function for a TRS Pathway.

### The first strategy for identifying TRS Pathways: three constraints to reduce the search space for TRS Pathways

The first strategy for identifying TRS Pathways is to reduce the search space of the entire network for TRS Pathways by three constraints. The three constraints are based on three previous approaches mining candidate signaling pathways from PPI data, given a pair of starting and ending proteins [12,22,23]. Firstly, we search for TRS Pathways relaying a signal from 1,728 start proteins to 479 end proteins. According to the definition of signaling pathways by which cells convert extracellular signals into cellular responses [1], start proteins are defined as ligand or transmembrane proteins; end proteins as transcription factor proteins or some proteins whose roles are clearly known in cells. 1,728 Start proteins and 479 end proteins were found by searching three databases: 1,310 trans-membrane proteins as start proteins from the Locate database [24]; 236 transcription factors as end proteins from the PD interaction data; 418 start (e.g. ligand proteins) and 243 end proteins (roles are clearly known in cells, e.g. caspase8) from the KEGG database [20]. These 243 end proteins are terminal nodes of KEGG signaling pathways linked to other signaling pathways or biological processes such as apoptosis.

Secondly, because signals are transmitted from an extracellular region into a nucleus, we defined the right orders of pathways as the following: from an extracellular region to a plasma membrane, from a plasma membrane to cytoplasm and from cytoplasm to a nucleus. When we search for TRS Pathways, we remove edges whose directions are opposite to the right orders such as from a plasma membrane to an extracellular region. Removing the opposite edges can lead to the loss of some feedback regulation processes between PPIs, but is an indispensible step to make the complex PPI data into a simple signaling pathway model to deal with. Moreover, since we can identify the transcriptional feedback regulation processes from the PD interaction data, the processes can compensate the loss defect. The sub-cellular localization data were from Locate database [24]. Lastly, we search for TRS Pathways whose path lengths are less than or equal to 10, since the path lengths of all signaling paths in the KEGG database [20] are distributed from 1 to 10 (Figure S2). We search the KEGG database for all signaling paths with the found start and end proteins, then count their path lengths.

### The second strategy for identifying TRS Pathways: the scoring function and search algorithm

The other strategy is to find the pathways only with the top scores by defining the scoring function for a TRS Pathway. Let *N *and *E *represent a set of nodes and edges of a TRS Pathway respectively. The scoring function P(*N,E*) is the sum of the two functions T(*N*) and R(*E*);(1)

T(*N*) measures how many nodes in a TRS Pathway are regulated by transcription. This measure is based on the method of Ideker et al. [8].(2)

Z_*start *_and Z_*end *_represent the Z score values of the start and end nodes of a TRS Pathway. Z score Z_*i *_= Φ^-1^(1-*p*_*i*_) is converted from the p value *p*_*i *_of a gene *i *to a significance level, where Φ^-1^is the inverse normal cumulative density function. The *p*_*i *_value is acquired from the results of the expression data analysis.(3)

We sum the Z_*i *_over all |*N*| genes in a TRS Pathway to produce an aggregate Z score (Z_*N*_) for a TRS Pathway. Then, in order to properly capture the connection between expression and network topology, we investigate whether the score Z_*N *_of a TRS Pathway in Eq. (3) is higher than expected relative to a random set of genes. We randomly take 100,000 samples from all gene sets of size *k *using a Monte Carlo approach and calculate their scores Z_*N*_. The mean μ_*k *_and standard deviation (SD) σ_*k *_for each *k *are estimated and the noise in the estimates is reduced using a sliding window average. Using these estimates, the corrected score S(*N*) is calculated. The corrected score of random TRS Pathways is guaranteed to have a mean of μ = 0 and SD σ = 1.

R(*E*) estimates how reliable PPIs in a TRS Pathway are. Estimating the PPI reliability R(*E*) of a TRS Pathway is absolutely necessary to indentify the signaling pathways consisting of PPIs [12], because the PPI data are noisy [11]. In this study, we adopt Bebek and Yang's method based on a logistic regression model [25]. The model represents the probability of a true interaction  as a function of four observed random variables  on a pair of proteins: (1) the observed number of papers in which the interaction between two proteins was observed, (2) the Pearson correlation coefficient of expression measurements of the corresponding genes, (3) the proteins' small world clustering coefficient [26], and (4) the binary (0/1) protein subcellular localization data of interacting proteins [27]. When interacting proteins co-localize in the same subcellular location, we give 1 to the interacting protein pair.

Given positive and negative training data sets, one can optimize the parameters (β_0_,..., β_4_) to maximize the likelihood of the data. To optimize the parameters, we use the lrm function of the Design R package. We randomly select 5,000 PPIs from 12,363 determined by coimmunoprecipitation as our positive training data set [28]. For the negative training data set, we also randomly select 5,000 PPIs that are not in the interactions. Firstly, we selected 1,000 PPIs for each positive and negative training data as carried out by Bebek and Yang [12]. However, since the number of the entire PPIs was higher than that of Bebek and Yang, we had to increase the training data set to 5,000 PPIs. We repeat these experiments 1,000 times and estimate the mean reliability of each PPI. To calculate a reliability score R(*E*) of a TRS Pathway, we take the same procedure of estimating the corrected expression score S(*N*) from the Z_*N *_scores of a TRS Pathway. For an edge of TRS Pathways, we did not use PPIs which reliability scores were less than 0.6 (about 85 quantile) (Figure S3).(4)

To search for the top path score TRS Pathways, we used Dijkstra's algorithm [29]. Although we reduce the search space for TRS Pathways by three constraints, it is still impossible to search for all possible TRS Pathways in a few days. Also, we do not have to find them all. However, other alternative pathways of the same start and end nodes may also be important, just as the highest-scoring path in cell biology. Therefore, we slightly modified Dijkstra's algorithm, which searches for only the smallest-scoring path, to identify TRS Pathways with several top path scores. The statistical significance of TRS Pathways is measured by randomly permutating the expression of individual genes and performing the same search for TRS Pathways 1,000 times.

### Identification of TRS Networks by a search algorithm

The following is the pseudo code of a search algorithm for a TRS Network. Input: an entire omics network G, all TRS Pathways relevant to the context, and a start gene. If the start gene is not given, the start gene is determined as the start node of the highest scoring TRS Pathway.

Output: a sub-network G_p _of G

(1) Initialize G_p _by the top ranked TRS Pathway whose start node is the start gene

(2) Add the end node of the initial TRS Pathway into Queue;

(3) WHILE end nodes exist in Queue

(4)  POLL an end node e,

(5)   FOR i = 1 to n of outgoing edges of e;

(6)    IF a target node t_i _of e_i _is a DEG (Differentially Expressed Gene) or exists in G_p_, add t_i _and e_i _into G_p_;

(7)    IF added t_i _is a start node, a DEG, and positively regulated, add the top ranked TRS Pathways of t_i _into G_p _and add the end node of the top ranked TRS Pathways into Queue;

(8)    IF added t_i _is an end and DEG, add t_i _into Queue;

(9) Output *G*_p _and its score.

The above search algorithm represents the following; by the top ranked TRS Pathway, the signal is transmitted into the end nodes. The activated end nodes carry out their own roles in cells; transcription feedback regulation, enzyme activity or inducing the expression of new start proteins. The newly activated start proteins can send signals to other end nodes, thus we added the TRS Pathways activated by start proteins of a positively regulated DEG into the TRS Network. Since there are so many TRS Pathways at context, we have to select only the highly significant TRS Pathways for adding a TRS Network. The newly added end nodes also carry out their own roles in cells. Therefore, we repeat this process until there is no protein in the Queue.

### The scoring function for TRS Networks for sub-network comparison

The scoring function for a TRS Network SF comprises three parts: how much its nodes are differentially expressed S, how many significant TRS Pathways P it includes, and how many edges || it has.(5)(6)(7)

here .

In order to calculate S, we use the same function for the expression score of a TRS Pathway (See Eq. (3)). P_*i *_is the score value (See Eq. (1)) of the *i*^th ^TRS Pathway in a TRS Network. *N*_*i *_and *E*_*i *_also represent nodes and edges of *i*^th ^TRS Pathway. *N*_0 _and *E*_0 _represent a set of nodes and a set of edges which do not belong to any TRS Pathway, respectively. They have a role to inter-connect TRS Pathways. To assess the statistical significance of TRS Networks, we take the same procedure in estimating the TRS Pathways by randomly permutating the expression of individual genes and performing the same search for TRS Networks 1,000 times.

### Expression data analysis

We investigated dendritic cells treated with lipopolysaccharide (LPS), one of the TLR agonists. TLRs are very important innate receptors that sense microbial products and trigger dendritic cell maturation and cytokine production, effectively bridging innate and adaptive immunity [30]. We downloaded GSE2706 data from the GEO database [31] and analyzed the data with limma [32]. The limma results were used in three different ways. Firstly, we used the probability values (p values) of genes for the scoring function. Secondly, we selected genes for which the probability value was p < 0.05 as differential expressed genes for the identification of TRS Networks by a search algorithm. When we compared the performance of the TRS Network identification according to several DEG cut-off p values, p < 0.05 was one of the best performances (Figure S4). Lastly, we used fold changes of gene expression for the color of protein nodes in the networks.

### Kinase reaction annotation

Since phosphorylation is one of the most important and critical processes in the signal transduction pathway, we annotate TRS Networks with the relationships between kinases and their substrates [33,34]. We believe that the kinase reaction annotation makes the TRS Networks more reliable and more meaningful for a biological understanding.

## Results

### TRS Pathways found by searching KEGG pathways and the human omics network

To find which signaling paths are regulated by transcription in our known knowledge, we searched the KEGG pathways [20] for TRS Pathways in dendritic cells 2h after the LPS treatment. The top TRS Pathways can be ranked by the two scores: the expression score and the path score (Additional file 1: Table S1). The expression score is an aggregated expression score S(*N*) (Eq. (3)) and the path score is our own path scoring function (Eq. (1)). Among all in the expression score, the path from *TNF *to *MAPK12 (p38) *was the highest-scoring and among all in the path score, the path from *TNF *to *NFKB1 (p50) *was the highest-scoring.

From the viewpoint of understanding the underlying biological phenomena of cellular contexts, the path from *TNF *to *NFKB1 *found by our path scoring function is more helpful, though it is difficult to determine which is more significant than the other. Let us suppose that intermediate signaling proteins exist to some extent in a cell to quickly respond to an environmental signal. Then, when the mRNA expression level of the start node of a path increases, we expect that the signal starting from the start node would be transmitted to the end protein of the path. Likewise if the mRNA expression level of the end node of a path increases, we also expect that the biological process of the end node would occur. In the path from *TNF *to *NFKB*, both the mRNA expression levels of the start node *TNF *and the end node *NFKB1 *were increased. The signal starting from *TNF *would be transmitted to *NFKB1 *and *NFKB1 *would activate the mRNA expression levels of its target genes as a transcription factor. Therefore, we can identify more explicable paths by using our path scoring function, adding the expression scores of the start and end node of a path (Z_*start *_and Z_*end *_of Eq. (2)) into the aggregated expression score, than by using just the aggregated score.

A TRS Pathway from *TNF *to *NFKB1 *was the highest-scoring when we searched the omics network with our path scoring function (See Eq. (1) and Methods). We compared it with the highest-scoring TRS Pathway of KEGG, since both pathways have the same start and end nodes (Figure 1). However, their intermediate nodes were shown to be quite different. Unless we thought of the actual biological system where a protein complex is usually used rather than a single protein for sending a signal, we could not recognize that the TRS Pathway of the omics network is also correct. The TRS Pathway of the omics network (Figure 1C) seems to be constructed by selecting each protein from its corresponding protein complex of Figure 1A to make a linear path.

The signaling pathway of Figure 1A has not been included in KEGG pathways, but it was already known in a paper [35]. This shows that our TRS Pathway method suggests highly reliable TRS Pathway candidates by searching the omics network. Moreover, we also confirmed that our analysis can search not only highly reliable but also significantly regulated TRS Pathways from the omics network by assessing their significances quantitatively (See Methods). The TRS Pathway score (13.635) of the omics network (Figure 1C) was higher than that (11.936) of KEGG (Figure 1B), and the TRS Pathway p value (2.428e-4) of the omics network was lower than that (1.476e-3) of KEGG (Additional file 1: Table S2).

The first pathway sending a signal from LPS to the nuclear factor of kappa light polypeptide gene enhancer in B-cells (NFKB) was unfortunately hardly regulated by transcription. Not only was the path score low, but so was its expression score that we could not identify this pathway as a TRS Pathway. It was the correct result because the first pathway was not a TRS Pathway but a relevant signaling pathway in this context. These results shows that our TRS Pathway analysis can search highly reliable TRS Pathway candidates regulated by transcription according to cellular contexts well, even though it cannot guarantee to find all the relevant signaling pathways to the contexts. In our analysis, we fortunately knew that *TLR4 *senses LPS as the start protein that initiates all relevant signaling pathways. Therefore, we could identify transcriptional regulations between the first pathway and other signaling pathways in TRS Networks, though not when searching for TRS Pathways.

### TRS Networks found by searching the omics network

We found that the TLR signaling pathway and Apoptosis are the most relevant and known pathways among all the KEGG pathways 2h after the LPS treatment, by Impact analysis [36]. Impact analysis is one of the best methods ordering known pathways by estimating how each pathway is over-represented in a specific context. However, a major drawback to analyses using known pathways is that the large number of genes are uninvolved in pathways or have no functional classification [37]. Even though we discovered the over-represented pathways in the context of expression data, we could not sometimes find out the new understanding of the biological phenomenon. Therefore, these two pathways are not the TRS Networks we wanted to discover but the most relevant and known pathways in this context.

**Figure 2:**
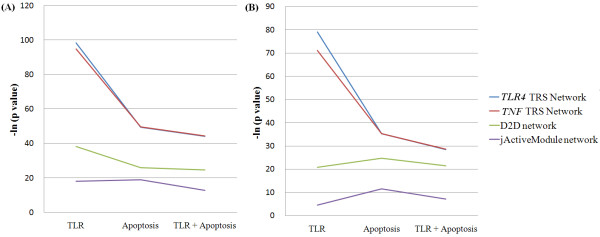
**The overlap of four sub-networks with the TLR signaling pathway and Apoptosis**. It shows the difference of the statistical significance of the node overlap (A) and the edge overlap (B) between four sub-networks. The line color of each sub-network is following: the TLR4 TRS Network (blue), the TNF TRS Network (red), the D2D network (green), and the jActiveModule network (purple). The overlaps of the two TRS Networks are larger than those of other networks: the D2D network and the jActiveModule Network. P values are measured by Fisher exact test [46] and are transformed by the negative natural logarithm. The condition is 2h after the LPS treatment.

We used two pathways, the TLR signaling pathway and Apoptosis, to prove that our TRS Network approach finds more known nodes and edges than other previous approaches. The larger the overlap of a network with the two known pathways, the more known nodes and edges the approach constructing the network finds. To compare, two previous methods inferring sub-networks from omics networks were used: a jActiveModule network and a D2D network. A D2D network is constructed by linking DEGs with their corresponding PPIs [7]. A jActiveModule network is inferred from the omics network using Cytoscape [38]. The jActiveModule analysis infers sub-networks perturbed at the condition with thousands of PPI data by devising an adequate scoring function on PPI networks based on the significant changes of expression data [8]. The overlaps of two TRS Networks (*TLR4 *TRS Network and *TNF *TRS Network) were superior to those of other two networks: the D2D network and the jActiveModule network (Figure 2 and Additional file 1: Table S3). It shows that our TRS Network approach finds more known nodes and edges than the previous approaches do.

The *TLR4 *TRS Network and *TNF *TRS Network were named by the start node used to search for TRS Networks (See Methods). To estimate the performance of our TRS Network analysis without knowing the start node, we searched for the two TRS Networks and compared their overlaps. In this context, TLR4 senses LPS as the start protein. If, however, we did not know the start protein, *TNF*, the start protein of the highest-scoring TRS Pathway would be selected as the start node of the search step. The *TLR4 *TRS Network was found by the actual start node and the *TNF *TRS Network was found by the inferred start node. The overall propensity of the overlaps of two networks looked very similar, though the performance by the actual start node was slightly better than by the inferred start node (Figure 2 and Additional file 1: Table S3). Therefore, this result shows that our TRS Network analysis works quite well on searching for TRS Networks without a known start protein.

By our TRS Network scoring function (Eq. (5)), we ordered 70 KEGG signaling pathways and four sub-networks: the *TLR4 *TRS Network, the *TNF *TRS Network, the jActiveModule network, and the D2D Network (Table 1). We compared the orders of KEGG pathways by our scoring function to those by Impact analysis. We obtained the same results in the top ranked KEGG pathways. By both analyses, the TLR pathway and Apoptosis were the most relevant to this context and highly regulated by transcription. However, the network scores (p values) of the two KEGG pathways were much lower (more significant) than those of the two TRS Networks. It shows that the two KEGG pathways are not good TRS Networks, though they are the most relevant to the context among the known pathways.

**Table 1:** The results of TRS Network analysis.

Networks or pathways	Network score (p value)	S	P	|*ε*|
***TLR4 *TRS Network**	**4.312 (<1.0e-03)**	**17.246**	**942.988**	**218**
***TNF *TRS Network**	**4.046 (<1.0e-03)**	**17.509**	**698.589**	**214**
TLR signaling pathway	2.078 (0.38)	5.173	32.789	213
Apoptosis	1.385 (0.83)	3.958	159.534	158
**D2D network**	**0.118 (0.99)**	**29.244**	**32.089**	**834**
**jActiveModule network**	**-0.194 (0.99)**	**41.025**	**19.484**	**970**

The network scores are calculated by summing InS, and InP and subtracting In|| (See Eq. (5)). S indicates how much its nodes are differentially expressed. is calculated by summing the path scores of all TRS Pathways the network includes. P Sub-networks results are highlighted in bold to be easily recognized from KEGG pathway results. P values are assessed by random permutation of the expression of individual genes. The expression condition is 2h after the LPS treatment.

Among 70 KEGG pathways and the four sub-networks, the highest-scoring pathway or sub-network was the *TLR4 *TRS Network (Table 1). It includes more significant TRS Pathways (P: 942.988) than the *TNF *TRS Network (P: 698.589) and the TLR signaling pathway (P: 32.789), though the number of its edges (| |: 218) is similar to that of the *TNF *TRS Network (| |: 214) and to that of the TLR signaling pathway (| |: 213). This suggests that edges of the *TLR4 *TRS Network have a higher probability in being the components of significant TRS Pathways than those of other networks. Thus, the *TLR4 *TRS Network is more explicable and the edges may be more important. The D2D network and jActiveModule network did not obtain high network scores despite their high expression scores (S: 41.025 and 29.244). The networks comprise many edges (| |: 834 and 970) but do not include a lot of TRS Pathways (P: 19.484 and 32.089). This shows that most edges are not components of TRS Pathways. The two sub-networks are inexplicable and do not provide many clues to understand biological phenomenon of the context. Therefore, the orders of the network scores (Table 1) shows that our TRS Network analysis efficiently finds more TRS Pathways and TRS Networks than other approaches (including canonical pathway analyses).

## Discussion

### TRS Network analysis shows how dendritic cells respond to the LPS treatment

**Figure 3:**
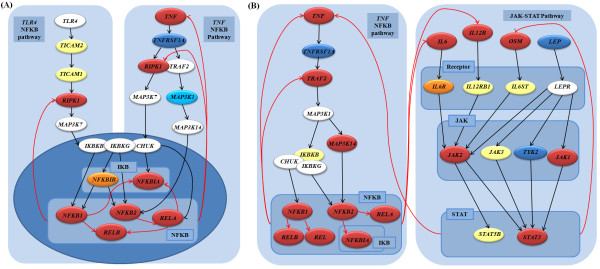
**Transcriptional regulations in dendritic cells treated with LPS**. (A) and (B) shows the transcriptional regulations between the two TRS Pathways at 2h and at 8h after the LPS treatment respectively. (A) At 2h after the treatment, TLR4 sensing LPS sends a signal to NFKB. NFKB reinforces to increase its own expression by increasing the mRNA expression level of TNF. (B) At 8h after the treatment, NFKB activates the JAK-STAT pathway through increasing the mRNA expression levels of IL6, IL12B, and OSM. The nodes represent proteins and their colors show their mRNA expression levels (the same as Figure 1). Light cyan colored boxes represent gene families. The black edges represent PPIs and red edges do positive PD interactions. The positive PD interactions are estimated based on the expression change of source and target genes.

Dendritic cells sense the presence of LPS and are matured by presenting LPS as an antigen-presenting cell to initiate adaptive immune responses through T cell activation [1]. Our TRS Network analysis found that NFKB activated by the signal starting from LPS induces biological process related to the maturation of dendritic cells. We discovered four biological processes related to dendritic cell maturation and among those processes, two processes showed inter-pathway cross-communication of transcriptional regulations between signaling pathways (Figure 3).

Firstly, NFKB has gotten a positive feedback by inducing *TNF*, which in turn reinforces the activation of NFKB (Figure 3A). When the dendritic cells are treated with LPS, LPS activates *TLR4 *which recruits adaptor proteins and sends a signal to NFKB to release NFKB into the nucleus (a *TLR4*-NFKB pathway). NFKB in the nucleus induces the transcription of genes that promote immune and inflammatory responses [1]. *TNF*, one of the target genes increased by NFKB, has the possibility to activate NFKB as a positive feedback by sending a signal to NFKB through a *TNF*-NFKB pathway. A positive feedback is possible because the *TLR4*-NFKB pathway and the *TNF*-NFKB pathway have a common downstream activating NFKB.

Secondly, NFKB activated by the *TNF*-NFKB pathway induces inflammatory responses by increasing the mRNA expression levels of inflammatory cytokines (e.g. *IL6 *and *IL12B*) (Figure 3B and Figure S5). The inflammatory cytokines *IL6 *and *IL2B *stimulates the JAK-STAT signaling pathway. The JAK-STAT signaling pathway provides one of the most direct routes to the nucleus in which transcriptional activation is initiated by each particular member of the STAT family [1]. In spite of the simple pathway that consists of four JAKs and seven STATs, the pathway translates more than 30 cytokines into cell-type specific or context-dependent patterns of cytokine responsiveness and gene expression [39]. In this context, cytokine *IL6*, *IL12B*, and *OSM *were activated by the *TNF*-NFKB pathway and the cytokines increased the mRNA expression levels of the interferon regulatory transcription factor family genes, such as *IRF1 *and *IRF7 *by the JAK-STAT signaling pathway. The chosen genes were transcription factors involved in inflammation and apoptosis [40]. This inter-pathway cross-communication is a good example of transcriptional regulations between signaling pathways.

Thirdly, NFKB elevated the mRNA expression levels of necessary genes for T cell stimulation: *CD40*, *CD80*, *ICAM1*, *CD83*, *CXCL10*, *CCL5*, and *CXCL11 *(Figure S5). *CD40 *and *CD80 *are co-stimulatory molecules that bind to complementary receptors on the T cell surface, in activating a T cell [1]. *ICAM1 *enables a T cell to remain bound to an antigen-presenting cell long enough for the T cell to become activated by binding *lfa-1 *on the T cell surface [41]. Moreover, it was reported that the mature dendritic cells expressed *CD83 *and high levels of *CD40*, *CD80*, and *CD86 *[42]. *CXCL10*, *CXCL11*, and *CCL5 *are chemoattractants that guide the migrations of leukocytes such as T cells to induce a suitable immune response [43-45].

Lastly, NFKB increases the mRNA expression levels of genes related to apoptosis, especially inhibitors of apoptosis (IAPs) such as *BIRC2 *and *BIRC3 *suppressing caspases (Figure S5) [35]. At 2h after the LPS treatment, mRNA expression levels of IAPs were only increased. However, at 8h after the treatment, those of other genes inhibiting apoptosis (*IL15*, *NFKB1*) were also increased. This result shows that apoptosis would be repressed in dendritic cells and it agrees with this context of the dendritic cell maturation. If apoptosis occurred in matured dendritic cells, they could not perform their roles initiating adaptive immune responses as an antigen-presenting cell.

## Conclusions

We propose a new computational omics approach to discover signaling pathways regulated by transcription (TRS Pathways) and transcriptional regulations between them in TRS Networks dependent on cellular contexts to investigate the transcription-mediated mechanism of inter-pathway cross-communication of signaling pathways. Our new approach has three advantages. Firstly, highly reliable TRS Pathway candidates and transcriptional regulations between pathways can be discovered. Secondly, the approach can discover more known knowledge than the previous approaches. Lastly, it helps to understand the underlying biological phenomena of cellular contexts by providing a graphical network. We demonstrated that our analysis performed well in the context of dendritic cells treated with LPS, since dendritic cells commanding the human immune system are very important to disease research. Likewise, our approach determines how some signaling pathways are transcriptionally regulated by other pathways in patients by analyzing expression data comprising patients and normal samples. Therefore, it would be very helpful to understand the underlying biological phenomena of complex diseases such as cancer.

## Authors' contributions

SH, HS and DL conceived and designed the experiments. SH and SW analyzed the data. SH wrote the manuscript. DL directed this project and helped to draft the manuscript. All authors read and approved the final manuscript.

## Supplementary Material

Additional file 1 **Supplementary materials**. It comprises 5 figures (Figure S1, Figure S2, Figure S3, Figure S4, and Figure S5), 3 tables (Additional file 1: Table S1, Table S2, Table S3) and 4 networks (the *TLR4 *TRS Network, the *TNF *TRS Network, the D2D Network and the jActiveModule network). Click here for file
