# Supplementary material for: Context-dependent transcriptional regulations between signal transduction pathways
Source: BMC Bioinformatics. 2011 Jan 13;12:19. doi: 10.1186/1471-2105-12-19 (PMC3225034; doi:10.1186/1471-2105-12-19)
Supplement: Additional file 1 — Supplementary materials. It comprises 5 figures (Figure S1, Figure S2, Figure S3, Figure S4, and Figure S5), 3 tables (Additional file 1: Table S1, Table S2, Table S3) and 4 networks (the TLR4 TRS Network, the TNF TRS Network, the D2D Network and the jActiveModule network). [file 1471-2105-12-19-S1.DOCX]

**Context-dependent transcriptional regulation between signal transduction pathways**

(Supplementary materials)

Sohyun Hwang^1^, Sangwoo Kim^1^, Heesung Shin^2^, Doheon Lee^1§^

^1^Department of Bio and Brain Engineering, KAIST, 373-1 Guseong-dong, Yuseong-gu, Deajeon, Republic of Korea

^2^Department of Mathematics, Inha University, 253 Yonghyun-dong, Nam-gu, Incheon, Republic of Korea

CONTENTS

[1. Supplementary figures 2](#_Toc282538555)

[Fig. S1 Comparing an OMICS data with KEGG database in terms of nodes and edges. 2](#_Toc282538556)

[Fig. S2 The probability distribution of the path length of signaling paths in KEGG database. 2](#_Toc282538557)

[Fig. S3 The distribution of PPI reliability scores. 3](#_Toc282538558)

[Fig. S4 The performance comparison of the TRS Network identification by DEG cut-off p values. 3](#_Toc282538559)

[Fig. S5 Target genes transcriptionally regulated by NFKB at 2h after a lipopolysaccharide treatment. 4](#_Toc282538560)

[2. Supplementary tables 4](#_Toc282538561)

[Table S1 Signaling pathways highly regulated by transcription among KEGG pathways. 5](#_Toc282538562)

[Table S2 TRS Pathways in dendritic cells at 2h after a lipopolysaccharide treatment. 6](#_Toc282538563)

[Table S3 Comparing the overlap of four sub-networks with that of the KEGG TLR pathway. 6](#_Toc282538564)

[3. Networks 6](#_Toc282538565)

[A. The *TLR4* TRS Network 6](#_Toc282538566)

[B. The *TNF* TRS Network 9](#_Toc282538567)

[C. The D2D network 12](#_Toc282538568)

[D. The jActiveModule network 21](#_Toc282538569)

[References 31](#_Toc282538570)

# Supplementary figures


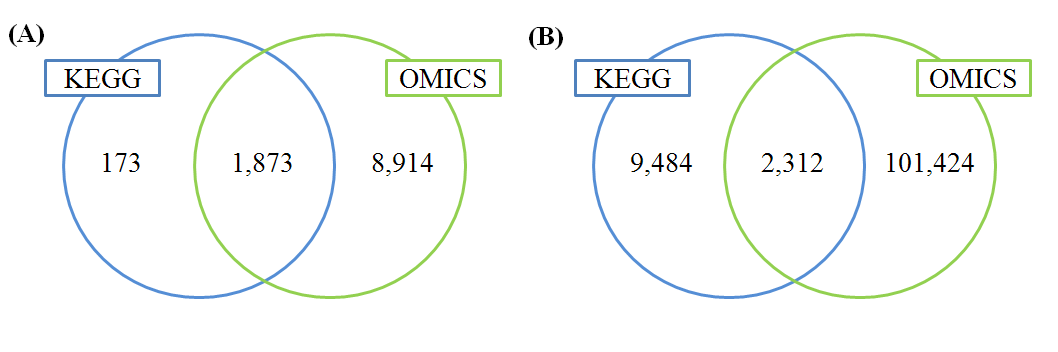


## Fig. S1 Comparing an OMICS data with KEGG database in terms of nodes and edges.

(A) and (B) are compared in terms of nodes and edges respectively. The OMICS data miss many interactions of KEGGs [3] that amount to about 80% of all the KEGG interactions. However, the KEGG data miss more interactions of OMICS (101,424) than the OMICS data (9,484). Therefore, the two things are necessary: investigating the OMICS data to find new signaling pathways and adding the KEGG data into an OMICS network.


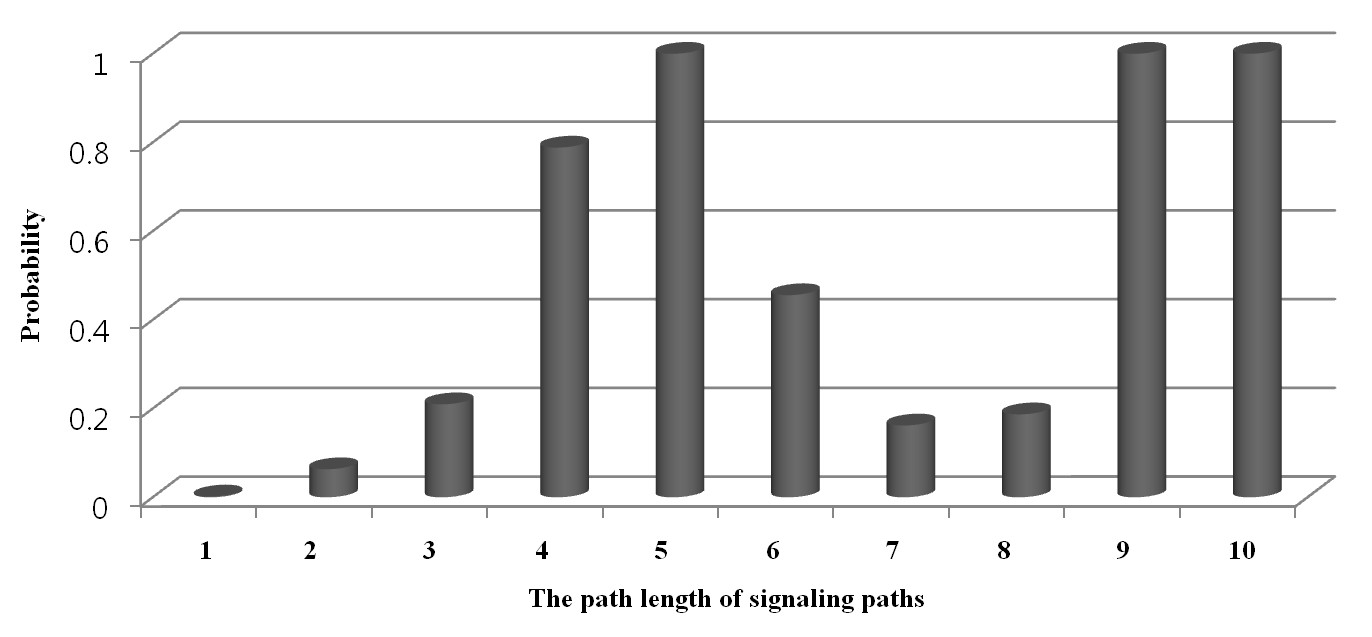


## Fig. S2 The probability distribution of the path length of signaling paths in KEGG database.

In order to estimate the probability distribution of the path length of the known signaling paths, we found all signaling paths in KEGG database with 1,728 start and 429 end proteins, then counted their path lengths. Since the counts of path length 9 and 10 were outliers, we modified the counts of 9 and 10 into the maximum count of path length 5 and then divided each count by the maximum count to get each probability. The definition of outliers is more than 1.5 times the interquartile range of the difference between the 1^st^ and 3^rd^ quartiles. Since their counts were distributed from 1 to 10, the possible range for the path lengths of TRS Pathways was set as less than or equal to 10.


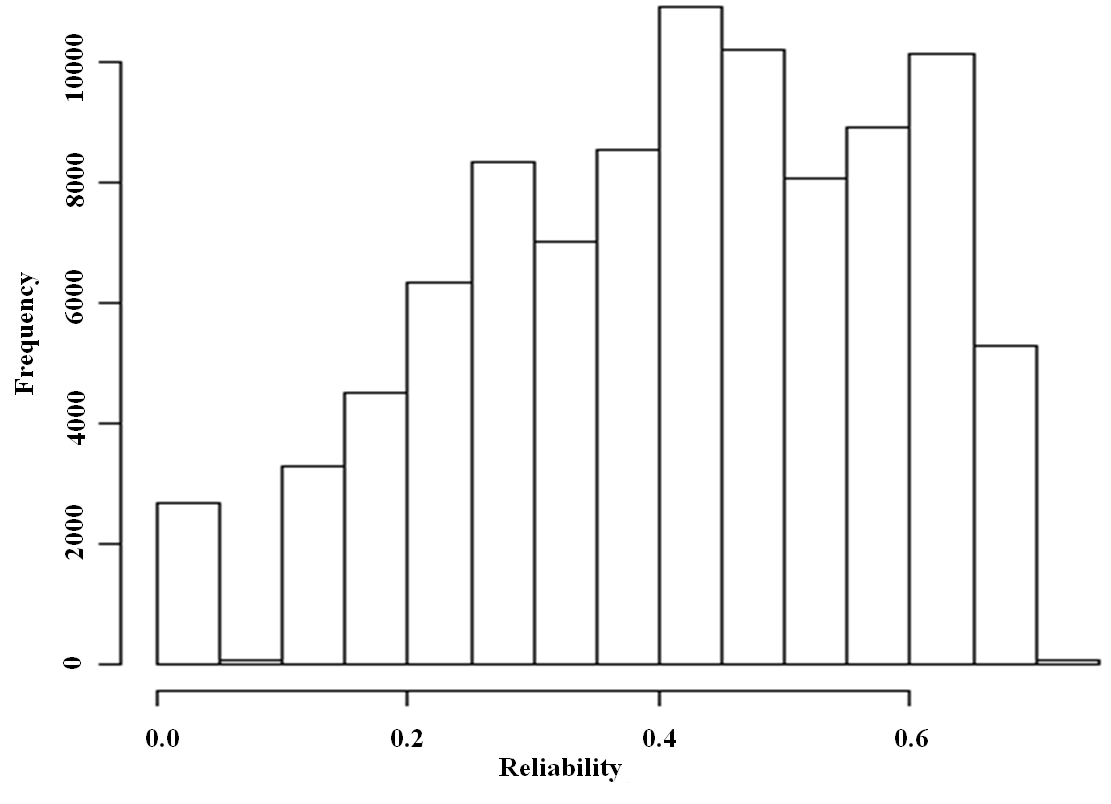


## Fig. S3 The distribution of PPI reliability scores.

To make TRS Pathways highly reliable, we removed protein protein interactions (PPIs) which reliability scores were less than 0.6 (about 85 quantile) while searching for TRS Pathways. This figure shows the distribution of PPI reliability scores. The mean value and standard deviation of PPI reliability scores were $\boldsymbol{\mu=}$0.4157 and $\boldsymbol{\sigma=}$ 0.1660.


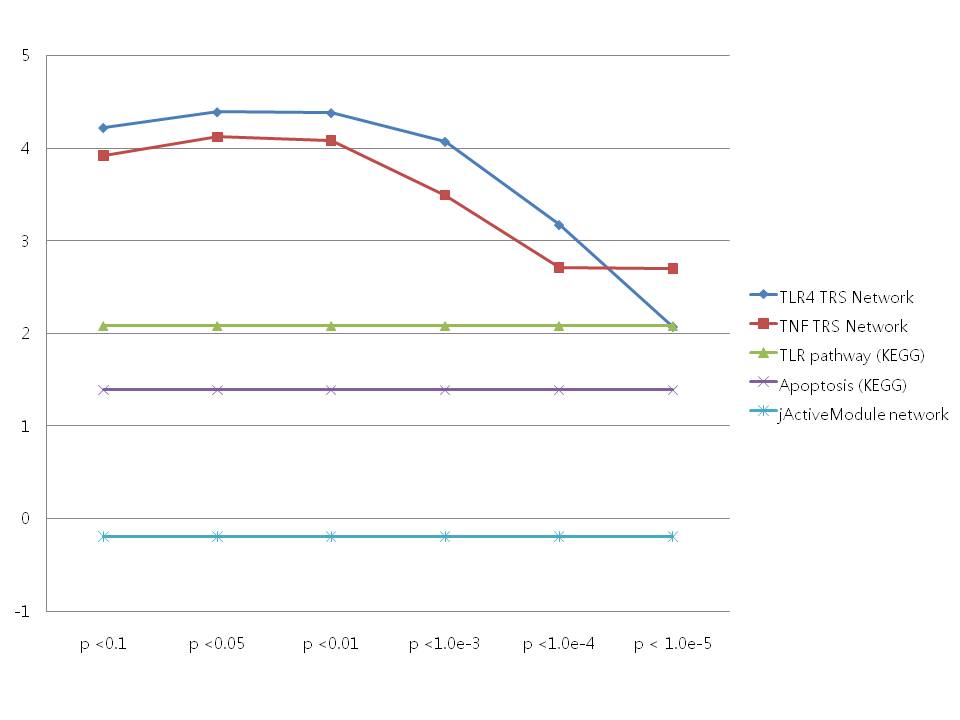


## Fig. S4 The performance comparison of the TRS Network identification by DEG cut-off p values.

When we compared the performances of the TRS Network identification according to several DEG cut-off p values, we found that the performances of DEGs determined by conventional cutoff scores (p value <0.05 and p value <0.01) were better than those of other DEG cut-off p values. We also found that the performances of both TLR4 and TNF TRS Network were always better than those of TLR pathway (KEGG), Apoptosis (KEGG) and jActiveModule network, regardless of changing the DEGs by several cut off p values. It supports that our method works better than the preexisting other methods.

**
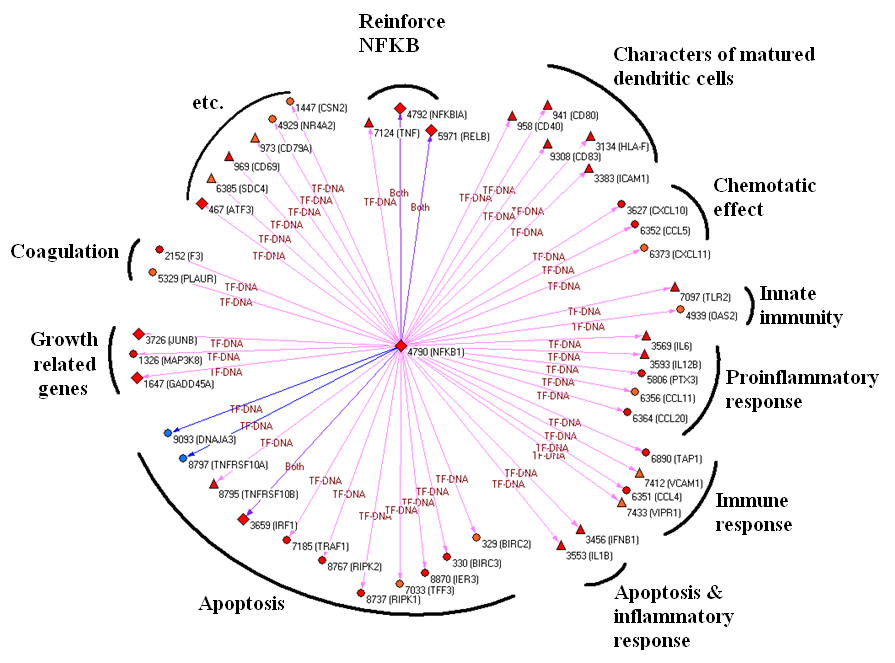
**

## Fig. S5 Target genes transcriptionally regulated by NFKB at 2h after a lipopolysaccharide treatment.

We found 46 genes that were differentially expressed genes as well as target genes of NFKB. They were grouped into ten by their biological function. Most groups are related to dendritic cell maturation and immune response in accordance with this cellular context. The number of each gene represents the Entrez gene id [1] and a gene symbol in parenthesis is the HGNC approved gene symbol [2]. Among 46 target genes, the expression of two genes (DNAJA3, TNFRSF10A) are repressed. It can be happened, because we have not fully understand the each transcriptional regulation process in detail; transcriptional regulation does not always occur positively and transcription can be regulated by several transcription factors or other auxillary proteins. Therefore, we think that TRS Pathways and Network in this situation provide some interesting clues to help understanding the expression context more deeply when we find out the cause.

# Supplementary tables

| Expression score (Rank) | Path score  (Rank) | Path |
| --- | --- | --- |
| 4.467 (1) | **10.505 (5)** | ***TNF* → *TNFRSF1A* (TNFR) → *TRAF2* → *MAP3K5 (ask1)* → *MAP2K3 (mkk3)* → *MAPK12 (p38)*** |
| 4.416 (2) | 10.328 (6) | *TNF* → *TNFRSF1A* (TNFR) → *TRAF2* → *MAP3K5 (ask1)* → *MAP2K3 (mkk3)* → *MAPK14 (p38)* |
| 4.413 (3) | 10.315 (7) | *TNF* → *TNFRSF1A* (TNFR) → *TRAF2* → *MAP3K5 (ask1)* → *MAP2K3 (mkk3)* → *MAPK11 (p38)* |
| 4.364 (4) | 8.915 (37) | *TNF* → *TNFRSF1A* (TNFR) → *FADD* → *CASP8* |
| 4.213 (5) | 9.625 (18) | *TNF* → *TNFRSF1A* (TNFR) → *TRAF2* → *MAP3K5 (ask1)* → *MAP2K3 (mkk3)* → *MAPK13 (p38)* |
| 3.258 (15) | **11.945 (1)** | ***TNF* → *TNFRSF1A* (TNFR) → *TRAF2* → *MAP3K1 (mekk1)* → *IKBKG (IKK gamma)* → *NFKB1 (p50)*** |
| 3.249 (16) | 11.936 (2) | *TNF* →  *TNFRSF1A* (TNFR) → *TRAF2* → *MAP3K1 (mekk1)* → *IKBKG (IKK beta)* → *NFKB1 (p50)* |
| 2.981 (28) | 11.668 (3) | *TNF* →  *TNFRSF1A* (TNFR) → *TRAF2* →  *MAP3K1 (mekk1)* → *CHUK (IKK αlpha)* → *NFKB1 (p50)* |
| 2.936 (30) | 10.643 (4) | *TNF* →  *TNFRSF1A* (TNFR) → *TRADD* → *TRAF2* → *MTOR (frap1)* → *IRS2* |

## Table S1 Signaling pathways highly regulated by transcription among KEGG pathways.

The path from TNF to MAPK12 (p38) in the first row was the most highly regulated by transcription among all in the expression score column (See Eq. (3)). Among all in the path score column (See Eq. (1)), the path from TNF to NFKB1 (p50) in the sixth row was the most highly regulated by transcription. The rows of the two paths are highlighted in bold. The expression condition is 2h after the LPS treatment.

| Path | Path Score | P value |
| --- | --- | --- |
| 7124 (*TNF*) → 7132 (*TNFRSF1A*) → 8737 (*RIPK1*) → 6885 (*MAP3K7*) → 8517 (*IKBKG*) → 4793 (*NFKBIB*) → 4790 (*NFKB1*) | 13.635 | 2.428e-04 |
| 7124 (*TNF*) → 7132 (*TNFRSF1A*) → 8737 (*RIPK1*) → 6885 (*MAP3K7*) → 6416 (*MAP2K4*) → 2318 (*FLNC*) → 3675 (*ITGA3*) → 1399 (*CRKL*) → 27 (*ABL2*) | 13.118 | 4.305e-04 |
| 7124 (*TNF*) → 7132 (*TNFRSF1A*) → 8737 (*RIPK1*) → 6885 (*MAP3K7*) → 6416 (*MAP2K4*) → 1399 (*CRKL*) → 5295 (*PIK3R1*)→ 695 (*BTK*) → 5335 (*PLCG1*) → 5535 (*PPP3R2*) → 4775 (*NFATC3*) | 12.899 | 5.425e-04 |
| 3569 (*IL6*) → 3570 (*IL6R*) → 3716 (*JAK1*) → 5295 (*PIK3R1*) → 3702 (*ITK*) → 3937 (*LCP2*) → 5777 (*PTPN6*) → 6850 (*SYK*) → 23533 (*PIK3R5*) → 2475 (*MTOR*) →8660 (*IRS2*) | 12.626 | 7.258e-04 |
| 3593 (*IL12B*) → 3595 (*IL12RB2*) → 3716 (*JAK1*) → 5295 (*PIK3R1*) → 3702 (*ITK*) → 3937 (*LCP2*) → 5777 (*PTPN6*) →6850 (*SYK*) →23533 (*PIK3R5*) → 2475 (*MTOR*) → 8660 (*IRS2*) | 12.559 | 7.800e-04 |
| 29760 (*BLNK*) → 695 (*BTK*) → 6850 (*SYK*) → 5295 (*PIK3R1*) → 3702 (*ITK*) → 3937 (*LCP2*) → 5335 (*PLCG1*) → 5588 (*PRKCQ*) → 84433 (*CARD11*) → 8517 (*IKBKG*) → 4790 (*NFKB1*) | 12.531 | 8.028e-04 |
| 3553 (*IL1B*) → 3556 (*IL1RAP*) → 8772 (*FADD*) → 4615 (*MYD88)* → 3654 (*IRAK1*) → 3265 (*HRAS*) → 5295 (*PIK3R1*) → 2475 (*MTOR*) → 8660 (*IRS2*) | 12.446 | 8.755e-04 |
| 3569 (*IL6*) → 3570 (*IL6R*) → 3716 (*JAK1*) → 5295 (*PIK3R1*) → 3702 (*ITK*) → 3937 *(LCP2*) → 5777 (*PTPN6*) → 6850 (*SYK*) → 23533 (*PIK3R5*) → 2475 (*MTOR*) → 8408 (*ULK1*) | 12.442 | 8.796e-04 |
| 7124 (*TNF*) → 7132 (*TNFRSF1A*) → 7186 (*TRAF2*) → 4214 (*MAP3K1*) → 8517 (*IKBKG*) → 4790 (*NFKB1*) | 11.945 | 1.463e-3 |
| 7124 (*TNF*) → 7132 (*TNFRSF1A*) → 7186 (*TRAF2*) → 4214 (*MAP3K1*) → 3551 (*IKBKB*) → 4790 (*NFKB1*) | 11.936 | 1.476e-3 |
| 7124 (*TNF*) → 7132 (*TNFRSF1A*) → 7186 (*TRAF2*) → 4214 (*MAP3K1*) → 1147 (CHUK) → 4790 (*NFKB1*) | 11.668 | 1.931e-3 |
| 7097 (*TLR2*) → 4615 (*MYD88*) → 8772 (*FADD*) → 3654 (*IRAK1*) → 353376 (*TICAM2*) → 148022 (*TICAM1*) → 8737 (*RIPK1*) → 6885 (*MAP3K7*) → 8517 (*IKBKG*) → 4793 (*NFKBIB*) → 4790 (*NFKB1*) | 11.596 | 2.070e-03 |
| 7124 (*TNF*) → 7132 (*TNFRSF1A*) → 8717 (*TRADD*) → 7186 (*TRAF2*) → 2475 (*MTOR*) → 8660 (*IRS2*) | 10.643 | 5.143e-3 |
| 7124 (*TNF*) → 7132 (*TNFRSF1A*) → 7186 (*TRAF2*) → 4217 (*MAP3K5*) → 5606 (*MAP2K3*) → 6300 *(MAPK12*) | 10.505 | 5.837e-3 |
| 7124 (*TNF*) → 7132 (*TNFRSF1A*) → 7186 (*TRAF2*) → 4217 (*MAP3K5*) → 5606 (*MAP2K3*) → 1432 (*MAPK14*) | 10.328 | 6.859e-3 |
| 7124 (*TNF*) → 7132 (*TNFRSF1A*) → 7186 (*TRAF2*) → 4217 (*MAP3K5*) → 5606 (*MAP2K3*) → 5600 (*MAPK11*) | 10.315 | 6,936e-3 |
| 7097 (*TLR2*) → 4615 (*MYD88*) → 51135 (*IRAK4*) → 7189 (*TRAF6*) → 6885 (*MAP3K7*) → 8517 (*IKBKG*) → 4790 (NFKB1) | 10.284 | 7.133e-3 |
| 7097 (*TLR2*) → 4615 (*MYD88*) → 51135 (*IRAK4*) → 7189 (*TRAF6*) → 6885 (*MAP3K7*) → 3551 (*IKBKB*) → 4790 (NFKB1) | 10.276 | 7.184e-3 |
| 7124 (*TNF*) → 7132 (*TNFRSF1A*) → 8717 (*TRADD*) → 7186 (*TRAF2*) → 8517 (*IKBKG*) → 8660 (*IRS2*) | 10.094 | 8.449e-3 |
| 7124 (*TNF*) → 7132 (*TNFRSF1A*) → 8717 (*TRADD*) → 7186 (*TRAF2*) → 3551 (*IKBKB*) → 8660 (*IRS2*) | 10.086 | 8.512e-3 |
| 7124 (*TNF*) → 7132 (*TNFRSF1A*) → 8717 (*TRADD*) → 7186 (*TRAF2*) → 2475 (*MTOR*) → 8471 (*IRS4*) | 10.063 | 8.678e-3 |
| 7097 (*TLR2*) → 4615 (*MYD88*) → 51135 (*IRAK4*) → 7189 (*TRAF6*) → 6885 (*MAP3K7*) → 1147 (*CHUK*) → 4790 (*NFKB1*) | 10.028 | 8.959e-3 |

## Table S2 TRS Pathways in dendritic cells at 2h after a lipopolysaccharide treatment.

The number of each gene represents the Entrez gene id [1] and a gene symbol in parenthesis is the HGNC approved gene symbol [2]. The signalificance of TRS Pathways were assessed by random permutation. We randomly permutate the expression of individual genes and perform the same search for TRS Pathways 1,000 times.

| Network | nodes | overlap of  nodes | significance of nodes (p value) | edges | overlap of  edges | significance of edges (p value) |
| --- | --- | --- | --- | --- | --- | --- |
| *TLR4*  TRS Network | 146 | 36 | 1.964e-43 | 218 | 24 | 4.243e-35 |
| *TNF*  TRS Network | 144 | 35 | 6.248e-42 | 213 | 22 | 1.411e-31 |
| D2D network | 314 | 25 | 2.785e-17 | 834 | 14 | 9.474e-10 |
| jActiveModule network | 270 | 15 | 1.711e-8 | 970 | 6 | 1.059e-2 |

## Table S3 Comparing the overlap of four sub-networks with that of the KEGG TLR pathway.

The KEGG TLR pathway comprises 99 nodes and 213 edges. Statistical significance (p value) was measured by Fisher exact test. The *TLR4* TRS Network and *TNF* TRS Network were named by the start node used for searcing TRS networks. The D2D network was constructed by linking differentially expressed genes. The jActiveModule analysis inferred sub-networks perturbed at the condition with thousands of PPI data by devising an adequate scoring function on PPI networks based on the significant changes of expression data [4].

# Networks

We provide the four networks used for the Table S3 as a pajek format [5].

## The *TLR4* TRS Network

*Vertices 146

1 "185 (AGTR1)" triangle s_10 ic Orange bw 10 fos 15

2 "208 (AKT2)" diamond s_10 ic White bw 10 fos 15

3 "329 (BIRC2)" ellipse s_6 ic Orange fos 15

4 "330 (BIRC3)" ellipse s_6 ic Red fos 15

5 "467 (ATF3)" diamond s_10 ic Red bw 10 fos 15

6 "602 (BCL3)" diamond s_10 ic Orange bw 10 fos 15

7 "675 (BRCA2)" diamond s_10 ic White bw 10 fos 15

8 "701 (BUB1B)" ellipse s_6 ic LightYellow fos 15

9 "801 (CALM1)" ellipse s_6 ic LightYellow fos 15

10 "810 (CALML3)" ellipse s_6 ic Yellow fos 15

11 "861 (RUNX1)" diamond s_10 ic LightCyan bw 10 fos 15

12 "867 (CBL)" diamond s_10 ic Cyan bw 10 fos 15

13 "925 (CD8A)" triangle s_10 ic Orange bw 10 fos 15

14 "930 (CD19)" triangle s_10 ic White bw 10 fos 15

15 "940 (CD28)" triangle s_10 ic LightYellow bw 10 fos 15

16 "941 (CD80)" triangle s_10 ic Red bw 10 fos 15

17 "958 (CD40)" triangle s_10 ic Red bw 10 fos 15

18 "969 (CD69)" triangle s_10 ic Red bw 10 fos 15

19 "973 (CD79A)" triangle s_10 ic Orange bw 10 fos 15

20 "1027 (CDKN1B)" diamond s_10 ic Blue bw 10 fos 15

21 "1030 (CDKN2B)" ellipse s_6 ic Orange fos 15

22 "1326 (MAP3K8)" ellipse s_6 ic Red fos 15

23 "1445 (CSK)" ellipse s_6 ic Cyan fos 15

24 "1447 (CSN2)" ellipse s_6 ic Orange fos 15

25 "1452 (CSNK1A1)" ellipse s_6 ic LightYellow fos 15

26 "1457 (CSNK2A1)" ellipse s_6 ic White fos 15

27 "1487 (CTBP1)" ellipse s_6 ic White fos 15

28 "1592 (CYP26A1)" ellipse s_6 ic Blue fos 15

29 "1647 (GADD45A)" diamond s_10 ic Red bw 10 fos 15

30 "1736 (DKC1)" ellipse s_6 ic NavyBlue fos 15

31 "1786 (DNMT1)" ellipse s_6 ic NavyBlue fos 15

32 "1796 (DOK1)" ellipse s_6 ic Blue fos 15

33 "1856 (DVL2)" ellipse s_6 ic Cyan fos 15

34 "2107 (ETF1)" ellipse s_6 ic Orange fos 15

35 "2152 (F3)" ellipse s_6 ic Red fos 15

36 "2767 (GNA11)" ellipse s_6 ic Cyan fos 15

37 "3064 (HD)" ellipse s_6 ic Cyan fos 15

38 "3106 (HLA-B)" ellipse s_6 ic Orange fos 15

39 "3134 (HLA-F)" triangle s_10 ic Red bw 10 fos 15

40 "3265 (HRAS)" ellipse s_6 ic NavyBlue fos 15

41 "3320 (HSP90AA1)" ellipse s_6 ic LightCyan fos 15

42 "3383 (ICAM1)" triangle s_10 ic Red bw 10 fos 15

43 "3399 (ID3)" ellipse s_6 ic Blue fos 15

44 "3456 (IFNB1)" triangle s_10 ic Red bw 10 fos 15

45 "3459 (IFNGR1)" ellipse s_6 ic Blue fos 15

46 "3553 (IL1B)" triangle s_10 ic Red bw 10 fos 15

47 "3556 (IL1RAP)" triangle s_10 ic Red bw 10 fos 15

48 "3569 (IL6)" triangle s_10 ic Red bw 10 fos 15

49 "3570 (IL6R)" ellipse s_6 ic Orange fos 15

50 "3587 (IL10RA)" ellipse s_6 ic Red fos 15

51 "3593 (IL12B)" triangle s_10 ic Red bw 10 fos 15

52 "3595 (IL12RB2)" ellipse s_6 ic Red fos 15

53 "3627 (CXCL10)" ellipse s_6 ic Red fos 15

54 "3654 (IRAK1)" ellipse s_6 ic LightCyan fos 15

55 "3659 (IRF1)" diamond s_10 ic Red bw 10 fos 15

56 "3688 (ITGB1)" triangle s_10 ic White bw 10 fos 15

57 "3702 (ITK)" ellipse s_6 ic Orange fos 15

58 "3716 (JAK1)" ellipse s_6 ic Yellow fos 15

59 "3726 (JUNB)" diamond s_10 ic Red bw 10 fos 15

60 "3803 (KIR2DL2)" ellipse s_6 ic Yellow fos 15

61 "3937 (LCP2)" ellipse s_6 ic Red fos 15

62 "4089 (SMAD4)" diamond s_10 ic White bw 10 fos 15

63 "4615 (MYD88)" ellipse s_6 ic Yellow fos 15

64 "4616 (GADD45B)" diamond s_10 ic Orange bw 10 fos 15

65 "4790 (NFKB1)" diamond s_10 ic Red bw 10 fos 15

66 "4792 (NFKBIA)" diamond s_10 ic Red bw 10 fos 15

67 "4793 (NFKBIB)" ellipse s_6 ic Orange fos 15

68 "4929 (NR4A2)" ellipse s_6 ic Orange fos 15

69 "4939 (OAS2)" ellipse s_6 ic Orange fos 15

70 "5054 (SERPINE1)" diamond s_10 ic Red bw 10 fos 15

71 "5170 (PDPK1)" ellipse s_6 ic NavyBlue fos 15

72 "5295 (PIK3R1)" ellipse s_6 ic Blue fos 15

73 "5329 (PLAUR)" ellipse s_6 ic Orange fos 15

74 "5330 (PLCB2)" ellipse s_6 ic LightCyan fos 15

75 "5335 (PLCG1)" ellipse s_6 ic White fos 15

76 "5336 (PLCG2)" ellipse s_6 ic Cyan fos 15

77 "5371 (PML)" ellipse s_6 ic Yellow fos 15

78 "5501 (PPP1CC)" ellipse s_6 ic NavyBlue fos 15

79 "5502 (PPP1R1A)" ellipse s_6 ic LightYellow fos 15

80 "5584 (PRKCI)" ellipse s_6 ic LightCyan fos 15

81 "5586 (PKN2)" ellipse s_6 ic LightCyan fos 15

82 "5588 (PRKCQ)" ellipse s_6 ic White fos 15

83 "5590 (PRKCZ)" ellipse s_6 ic White fos 15

84 "5606 (MAP2K3)" ellipse s_6 ic Red fos 15

85 "5777 (PTPN6)" ellipse s_6 ic NavyBlue fos 15

86 "5806 (PTX3)" ellipse s_6 ic Red fos 15

87 "5837 (PYGM)" diamond s_10 ic White bw 10 fos 15

88 "5879 (RAC1)" diamond s_10 ic LightYellow bw 10 fos 15

89 "5932 (RBBP8)" ellipse s_6 ic Yellow fos 15

90 "5970 (RELA)" diamond s_10 ic Red bw 10 fos 15

91 "5971 (RELB)" diamond s_10 ic Red bw 10 fos 15

92 "6300 (MAPK12)" diamond s_10 ic White bw 10 fos 15

93 "6351 (CCL4)" ellipse s_6 ic Red fos 15

94 "6352 (CCL5)" ellipse s_6 ic Red fos 15

95 "6356 (CCL11)" ellipse s_6 ic Orange fos 15

96 "6364 (CCL20)" ellipse s_6 ic Red fos 15

97 "6373 (CXCL11)" ellipse s_6 ic Orange fos 15

98 "6385 (SDC4)" triangle s_10 ic Orange bw 10 fos 15

99 "6477 (SIAH1)" diamond s_10 ic Cyan bw 10 fos 15

100 "6507 (SLC1A3)" triangle s_10 ic Red bw 10 fos 15

101 "6517 (SLC2A4)" diamond s_10 ic Orange bw 10 fos 15

102 "6574 (SLC20A1)" ellipse s_6 ic NavyBlue fos 15

103 "6670 (SP3)" diamond s_10 ic Cyan bw 10 fos 15

104 "6714 (SRC)" ellipse s_6 ic Yellow fos 15

105 "6839 (SUV39H1)" ellipse s_6 ic NavyBlue fos 15

106 "6850 (SYK)" ellipse s_6 ic Blue fos 15

107 "6885 (MAP3K7)" ellipse s_6 ic LightCyan fos 15

108 "6890 (TAP1)" ellipse s_6 ic Red fos 15

109 "7033 (TFF3)" ellipse s_6 ic Orange fos 15

110 "7097 (TLR2)" triangle s_10 ic Red bw 10 fos 15

111 "7099 (TLR4)" triangle s_10 ic White bw 10 fos 15

112 "7124 (TNF)" triangle s_10 ic Red bw 10 fos 15

113 "7132 (TNFRSF1A)" triangle s_10 ic Blue bw 10 fos 15

114 "7185 (TRAF1)" ellipse s_6 ic Red fos 15

115 "7187 (TRAF3)" ellipse s_6 ic Yellow fos 15

116 "7189 (TRAF6)" ellipse s_6 ic LightCyan fos 15

117 "7329 (UBE2I)" ellipse s_6 ic LightCyan fos 15

118 "7412 (VCAM1)" triangle s_10 ic Orange bw 10 fos 15

119 "7433 (VIPR1)" triangle s_10 ic Orange bw 10 fos 15

120 "7534 (YWHAZ)" ellipse s_6 ic LightYellow fos 15

121 "8312 (AXIN1)" ellipse s_6 ic Cyan fos 15

122 "8398 (PLA2G6)" diamond s_10 ic LightYellow bw 10 fos 15

123 "8517 (IKBKG)" ellipse s_6 ic White fos 15

124 "8737 (RIPK1)" ellipse s_6 ic Red fos 15

125 "8767 (RIPK2)" ellipse s_6 ic Red fos 15

126 "8772 (FADD)" ellipse s_6 ic Blue fos 15

127 "8795 (TNFRSF10B)" triangle s_10 ic Red bw 10 fos 15

128 "8797 (TNFRSF10A)" ellipse s_6 ic NavyBlue fos 15

129 "8870 (IER3)" ellipse s_6 ic Red fos 15

130 "9093 (DNAJA3)" ellipse s_6 ic NavyBlue fos 15

131 "9134 (CCNE2)" diamond s_10 ic Red bw 10 fos 15

132 "9184 (BUB3)" ellipse s_6 ic Cyan fos 15

133 "9308 (CD83)" triangle s_10 ic Red bw 10 fos 15

134 "9641 (IKBKE)" ellipse s_6 ic Red fos 15

135 "10125 (RASGRP1)" ellipse s_6 ic LightYellow fos 15

136 "10746 (MAP3K2)" ellipse s_6 ic White fos 15

137 "11261 (CHP)" ellipse s_6 ic Yellow fos 15

138 "11344 (TWF2)" ellipse s_6 ic NavyBlue fos 15

139 "23118 (MAP3K7IP2)" ellipse s_6 ic Orange fos 15

140 "23533 (PIK3R5)" ellipse s_6 ic Orange fos 15

141 "27289 (RND1)" diamond s_10 ic Red bw 10 fos 15

142 "29984 (RHOD)" ellipse s_6 ic LightCyan fos 15

143 "51135 (IRAK4)" ellipse s_6 ic Cyan fos 15

144 "56288 (PARD3)" ellipse s_6 ic LightYellow fos 15

145 "148022 (TICAM1)" ellipse s_6 ic Yellow fos 15

146 "353376 (TICAM2)" ellipse s_6 ic Yellow fos 15

*Arcs

79 78 1.0 ap 0 c Black

50 58 1.0 ap 0 c Black

90 118 1.0 ap 0 c Lavender l TF-DNA

48 49 1.0 ap 0 c Black

123 67 1.0 ap 0 c Black

103 34 1.0 ap 0 c Blue l TF-DNA

61 75 1.0 ap 0 c Black

123 65 1.0 ap 0 c Black

137 79 1.0 ap 0 c Black

72 83 1.0 ap 0 c Black

72 57 1.0 ap 0 c Black

65 91 1.0 ap 0 c Purple l Both

65 130 1.0 ap 0 c Purple l Both

62 70 1.0 ap 0 c Black l TF-DNA

140 88 1.0 ap 0 c Black

65 44 1.0 ap 0 c Lavender l TF-DNA

41 139 0.6090037224768146 ap 0 c Black

65 22 1.0 ap 0 c Purple l Both

121 33 1.0 ap 0 c Black

54 40 0.6781178226079649 ap 0 c Black

72 80 1.0 ap 0 c Black

136 84 1.0 ap 0 c Black

90 64 1.0 ap 0 c Lavender l TF-DNA

65 51 1.0 ap 0 c Lavender l TF-DNA

57 75 1.0 ap 0 c Black

103 112 1.0 ap 0 c Blue l TF-DNA

83 144 1.0 ap 0 c Black

90 46 1.0 ap 0 c Lavender l TF-DNA

65 127 1.0 ap 0 c Lavender l TF-DNA

90 128 1.0 ap 0 c Blue l TF-DNA

59 62 1.0 ap 0 c Purple l Both

65 128 1.0 ap 0 c Blue l TF-DNA

90 48 1.0 ap 0 c Lavender l TF-DNA

36 74 1.0 ap 0 c Black

8 7 0.6820867698421389 ap 0 c Black

67 65 1.0 ap 0 c Black

9 37 1.0 ap 0 c Black

65 48 1.0 ap 0 c Lavender l TF-DNA

90 127 1.0 ap 0 c Lavender l TF-DNA

10 40 1.0 ap 0 c Black

75 81 0.630438026261182 ap 0 c Black

65 129 1.0 ap 0 c Lavender l TF-DNA

65 133 1.0 ap 0 c Lavender l TF-DNA

84 92 1.0 ap 0 c Black

112 113 1.0 ap 0 c Black

15 72 1.0 ap 0 c Black

71 2 1.0 ap 0 c Black

75 82 1.0 ap 0 c Black

85 50 1.0 ap 0 c Black

58 72 1.0 ap 0 c Black

55 118 1.0 ap 0 c Lavender l TF-DNA

126 25 0.6539582681000604 ap 0 c Black

76 122 1.0 ap 0 c Black

65 119 1.0 ap 0 c Lavender l TF-DNA

74 10 1.0 ap 0 c Black

117 103 0.6528420726732054 ap 0 c Black

110 63 1.0 ap 0 c Black

63 143 1.0 ap 0 c Black

103 102 1.0 ap 0 c Lavender l TF-DNA

20 131 1.0 ap 0 c Black

115 134 1.0 ap 0 c Black

103 35 1.0 ap 0 c Blue l TF-DNA

65 16 1.0 ap 0 c Lavender l TF-DNA

132 8 1.0 ap 0 c Black

105 11 0.6180705731103185 ap 0 c Black

62 43 1.0 ap 0 c Black l TF-DNA

120 31 0.6794494213283769 ap 0 c Black

57 61 1.0 ap 0 c Black

52 58 1.0 ap 0 c Black

26 77 0.6487006226498353 ap 0 c Black

65 109 1.0 ap 0 c Lavender l TF-DNA

29 64 0.19122411567153658 ap 0 c Black

103 70 1.0 ap 0 c Blue l TF-DNA

90 1 1.0 ap 0 c Lavender l TF-DNA

65 73 1.0 ap 0 c Lavender l TF-DNA

65 46 1.0 ap 0 c Lavender l TF-DNA

90 18 1.0 ap 0 c Lavender l TF-DNA

135 40 1.0 ap 0 c Black

42 75 1.0 ap 0 c Black

65 118 1.0 ap 0 c Lavender l TF-DNA

65 19 1.0 ap 0 c Lavender l TF-DNA

90 53 1.0 ap 0 c Lavender l TF-DNA

51 52 1.0 ap 0 c Black

39 13 1.0 ap 0 c Black

44 45 1.0 ap 0 c Black

23 104 1.0 ap 0 c Black

113 124 1.0 ap 0 c Black

65 17 1.0 ap 0 c Lavender l TF-DNA

90 42 1.0 ap 0 c Lavender l TF-DNA

90 115 1.0 ap 0 c Lavender l TF-DNA

90 116 1.0 ap 0 c Black l TF-DNA

142 141 1.0 ap 0 c Black

88 65 1.0 ap 0 c Black

116 132 0.6537272048436442 ap 0 c Black

90 114 1.0 ap 0 c Lavender l TF-DNA

81 136 0.660072001313463 ap 0 c Black

137 9 1.0 ap 0 c Black

65 114 1.0 ap 0 c Lavender l TF-DNA

65 53 1.0 ap 0 c Lavender l TF-DNA

65 35 1.0 ap 0 c Lavender l TF-DNA

139 116 1.0 ap 0 c Black

65 18 1.0 ap 0 c Lavender l TF-DNA

65 59 1.0 ap 0 c Lavender l TF-DNA

63 126 1.0 ap 0 c Black

103 28 1.0 ap 0 c Lavender l TF-DNA

65 3 1.0 ap 0 c Lavender l TF-DNA

64 29 0.19122411567153658 ap 0 c Black

31 105 0.6697679278342372 ap 0 c Black

5 94 1.0 ap 0 c Lavender l TF-DNA

65 4 1.0 ap 0 c Lavender l TF-DNA

90 94 1.0 ap 0 c Lavender l TF-DNA

90 93 1.0 ap 0 c Lavender l TF-DNA

25 121 1.0 ap 0 c Black

127 126 1.0 ap 0 c Black

103 110 1.0 ap 0 c Blue l TF-DNA

65 39 1.0 ap 0 c Lavender l TF-DNA

46 47 1.0 ap 0 c Black

90 66 1.0 ap 0 c Purple l Both

19 14 0.6667488312172034 ap 0 c Black

56 23 1.0 ap 0 c Black

90 7 1.0 ap 0 c Black l TF-DNA

75 137 1.0 ap 0 c Black

65 55 1.0 ap 0 c Purple l Both

90 3 1.0 ap 0 c Lavender l TF-DNA

103 30 1.0 ap 0 c Lavender l TF-DNA

131 20 1.0 ap 0 c Black

90 96 1.0 ap 0 c Lavender l TF-DNA

90 35 1.0 ap 0 c Lavender l TF-DNA

65 112 1.0 ap 0 c Lavender l TF-DNA

65 108 1.0 ap 0 c Lavender l TF-DNA

55 17 1.0 ap 0 c Lavender l TF-DNA

14 72 1.0 ap 0 c Black

27 89 0.6456182620846943 ap 0 c Black

124 107 1.0 ap 0 c Black

75 135 1.0 ap 0 c Black

90 4 1.0 ap 0 c Lavender l TF-DNA

90 129 1.0 ap 0 c Lavender l TF-DNA

103 52 1.0 ap 0 c Blue l TF-DNA

62 20 1.0 ap 0 c Black l TF-DNA

103 51 1.0 ap 0 c Blue l TF-DNA

116 146 0.6511494958816968 ap 0 c Black

88 90 1.0 ap 0 c Black

62 32 1.0 ap 0 c Black l TF-DNA

61 85 0.6093167987368007 ap 0 c Black

49 58 1.0 ap 0 c Black

38 60 1.0 ap 0 c Black

1 36 1.0 ap 0 c Black

107 123 1.0 ap 0 c Black

47 63 1.0 ap 0 c Black

10 87 1.0 ap 0 c Black

91 17 1.0 ap 0 c Lavender l TF-DNA

78 10 1.0 ap 0 c Black

106 140 1.0 ap 0 c Black

134 117 0.6442101598603441 ap 0 c Black

75 61 1.0 ap 0 c Black

146 145 1.0 ap 0 c Black

145 115 1.0 ap 0 c Black

65 66 1.0 ap 0 c Purple l Both

126 63 0.674752024399367 ap 0 c Black

65 5 1.0 ap 0 c Purple l Both

101 2 0.15880354132184865 ap 0 c Black

103 100 1.0 ap 0 c Blue l TF-DNA

2 20 1.0 ap 0 c Black

62 21 1.0 ap 0 c Black l TF-DNA

103 1 1.0 ap 0 c Blue l TF-DNA

65 68 1.0 ap 0 c Lavender l TF-DNA

40 72 1.0 ap 0 c Black

89 99 0.6011579672210154 ap 0 c Black

45 58 1.0 ap 0 c Black

37 27 0.6098905157501813 ap 0 c Black

65 93 1.0 ap 0 c Lavender l TF-DNA

71 41 0.626371237931438 ap 0 c Black

65 94 1.0 ap 0 c Lavender l TF-DNA

13 38 1.0 ap 0 c Black

65 124 1.0 ap 0 c Lavender l TF-DNA

104 72 1.0 ap 0 c Black

65 95 1.0 ap 0 c Lavender l TF-DNA

90 51 1.0 ap 0 c Lavender l TF-DNA

65 29 1.0 ap 0 c Lavender l TF-DNA

62 64 1.0 ap 0 c Black l TF-DNA

144 62 0.6119875531361855 ap 0 c Black

118 56 1.0 ap 0 c Black

33 26 1.0 ap 0 c Black

65 69 1.0 ap 0 c Lavender l TF-DNA

65 24 1.0 ap 0 c Lavender l TF-DNA

143 116 1.0 ap 0 c Black

5 18 1.0 ap 0 c Lavender l TF-DNA

80 120 0.6492549217905756 ap 0 c Black

10 137 1.0 ap 0 c Black

61 76 1.0 ap 0 c Black

65 96 1.0 ap 0 c Lavender l TF-DNA

72 71 1.0 ap 0 c Black

60 85 1.0 ap 0 c Black

90 86 1.0 ap 0 c Lavender l TF-DNA

77 105 0.6237971163248115 ap 0 c Black

82 123 1.0 ap 0 c Black

76 10 1.0 ap 0 c Black

90 44 1.0 ap 0 c Lavender l TF-DNA

85 106 1.0 ap 0 c Black

65 97 1.0 ap 0 c Lavender l TF-DNA

65 110 1.0 ap 0 c Lavender l TF-DNA

90 97 1.0 ap 0 c Lavender l TF-DNA

103 133 1.0 ap 0 c Blue l TF-DNA

103 21 1.0 ap 0 c Blue l TF-DNA

90 91 1.0 ap 0 c Purple l Both

16 15 1.0 ap 0 c Black

65 42 1.0 ap 0 c Lavender l TF-DNA

65 98 1.0 ap 0 c Lavender l TF-DNA

17 12 0.6102158566950838 ap 0 c Black

40 142 0.6051834770220744 ap 0 c Black

90 112 1.0 ap 0 c Lavender l TF-DNA

90 138 1.0 ap 0 c Blue l TF-DNA

111 63 1.0 ap 0 c Black

90 6 1.0 ap 0 c Lavender l TF-DNA

126 54 0.6987593798324877 ap 0 c Black

2 101 0.15880354132184865 ap 0 c Black

65 125 1.0 ap 0 c Lavender l TF-DNA

65 86 1.0 ap 0 c Lavender l TF-DNA

## The *TNF* TRS Network

*Vertices 144

1 "185 (AGTR1)" triangle s_10 ic Orange bw 10 fos 15

2 "208 (AKT2)" diamond s_10 ic White bw 10 fos 15

3 "329 (BIRC2)" ellipse s_6 ic Orange fos 15

4 "330 (BIRC3)" ellipse s_6 ic Red fos 15

5 "467 (ATF3)" diamond s_10 ic Red bw 10 fos 15

6 "602 (BCL3)" diamond s_10 ic Orange bw 10 fos 15

7 "675 (BRCA2)" diamond s_10 ic White bw 10 fos 15

8 "701 (BUB1B)" ellipse s_6 ic LightYellow fos 15

9 "801 (CALM1)" ellipse s_6 ic LightYellow fos 15

10 "810 (CALML3)" ellipse s_6 ic Yellow fos 15

11 "861 (RUNX1)" diamond s_10 ic LightCyan bw 10 fos 15

12 "867 (CBL)" diamond s_10 ic Cyan bw 10 fos 15

13 "925 (CD8A)" triangle s_10 ic Orange bw 10 fos 15

14 "930 (CD19)" triangle s_10 ic White bw 10 fos 15

15 "940 (CD28)" triangle s_10 ic LightYellow bw 10 fos 15

16 "941 (CD80)" triangle s_10 ic Red bw 10 fos 15

17 "958 (CD40)" triangle s_10 ic Red bw 10 fos 15

18 "969 (CD69)" triangle s_10 ic Red bw 10 fos 15

19 "973 (CD79A)" triangle s_10 ic Orange bw 10 fos 15

20 "1027 (CDKN1B)" diamond s_10 ic Blue bw 10 fos 15

21 "1030 (CDKN2B)" ellipse s_6 ic Orange fos 15

22 "1326 (MAP3K8)" ellipse s_6 ic Red fos 15

23 "1445 (CSK)" ellipse s_6 ic Cyan fos 15

24 "1447 (CSN2)" ellipse s_6 ic Orange fos 15

25 "1452 (CSNK1A1)" ellipse s_6 ic LightYellow fos 15

26 "1457 (CSNK2A1)" ellipse s_6 ic White fos 15

27 "1487 (CTBP1)" ellipse s_6 ic White fos 15

28 "1592 (CYP26A1)" ellipse s_6 ic Blue fos 15

29 "1647 (GADD45A)" diamond s_10 ic Red bw 10 fos 15

30 "1736 (DKC1)" ellipse s_6 ic NavyBlue fos 15

31 "1786 (DNMT1)" ellipse s_6 ic NavyBlue fos 15

32 "1796 (DOK1)" ellipse s_6 ic Blue fos 15

33 "1856 (DVL2)" ellipse s_6 ic Cyan fos 15

34 "2107 (ETF1)" ellipse s_6 ic Orange fos 15

35 "2152 (F3)" ellipse s_6 ic Red fos 15

36 "2767 (GNA11)" ellipse s_6 ic Cyan fos 15

37 "3064 (HD)" ellipse s_6 ic Cyan fos 15

38 "3106 (HLA-B)" ellipse s_6 ic Orange fos 15

39 "3134 (HLA-F)" triangle s_10 ic Red bw 10 fos 15

40 "3265 (HRAS)" ellipse s_6 ic NavyBlue fos 15

41 "3320 (HSP90AA1)" ellipse s_6 ic LightCyan fos 15

42 "3383 (ICAM1)" triangle s_10 ic Red bw 10 fos 15

43 "3399 (ID3)" ellipse s_6 ic Blue fos 15

44 "3456 (IFNB1)" triangle s_10 ic Red bw 10 fos 15

45 "3459 (IFNGR1)" ellipse s_6 ic Blue fos 15

46 "3553 (IL1B)" triangle s_10 ic Red bw 10 fos 15

47 "3556 (IL1RAP)" triangle s_10 ic Red bw 10 fos 15

48 "3569 (IL6)" triangle s_10 ic Red bw 10 fos 15

49 "3570 (IL6R)" ellipse s_6 ic Orange fos 15

50 "3587 (IL10RA)" ellipse s_6 ic Red fos 15

51 "3593 (IL12B)" triangle s_10 ic Red bw 10 fos 15

52 "3595 (IL12RB2)" ellipse s_6 ic Red fos 15

53 "3627 (CXCL10)" ellipse s_6 ic Red fos 15

54 "3654 (IRAK1)" ellipse s_6 ic LightCyan fos 15

55 "3659 (IRF1)" diamond s_10 ic Red bw 10 fos 15

56 "3688 (ITGB1)" triangle s_10 ic White bw 10 fos 15

57 "3702 (ITK)" ellipse s_6 ic Orange fos 15

58 "3716 (JAK1)" ellipse s_6 ic Yellow fos 15

59 "3726 (JUNB)" diamond s_10 ic Red bw 10 fos 15

60 "3803 (KIR2DL2)" ellipse s_6 ic Yellow fos 15

61 "3937 (LCP2)" ellipse s_6 ic Red fos 15

62 "4089 (SMAD4)" diamond s_10 ic White bw 10 fos 15

63 "4615 (MYD88)" ellipse s_6 ic Yellow fos 15

64 "4616 (GADD45B)" diamond s_10 ic Orange bw 10 fos 15

65 "4790 (NFKB1)" diamond s_10 ic Red bw 10 fos 15

66 "4792 (NFKBIA)" diamond s_10 ic Red bw 10 fos 15

67 "4793 (NFKBIB)" ellipse s_6 ic Orange fos 15

68 "4929 (NR4A2)" ellipse s_6 ic Orange fos 15

69 "4939 (OAS2)" ellipse s_6 ic Orange fos 15

70 "5054 (SERPINE1)" diamond s_10 ic Red bw 10 fos 15

71 "5170 (PDPK1)" ellipse s_6 ic NavyBlue fos 15

72 "5295 (PIK3R1)" ellipse s_6 ic Blue fos 15

73 "5329 (PLAUR)" ellipse s_6 ic Orange fos 15

74 "5330 (PLCB2)" ellipse s_6 ic LightCyan fos 15

75 "5335 (PLCG1)" ellipse s_6 ic White fos 15

76 "5336 (PLCG2)" ellipse s_6 ic Cyan fos 15

77 "5371 (PML)" ellipse s_6 ic Yellow fos 15

78 "5501 (PPP1CC)" ellipse s_6 ic NavyBlue fos 15

79 "5502 (PPP1R1A)" ellipse s_6 ic LightYellow fos 15

80 "5584 (PRKCI)" ellipse s_6 ic LightCyan fos 15

81 "5586 (PKN2)" ellipse s_6 ic LightCyan fos 15

82 "5590 (PRKCZ)" ellipse s_6 ic White fos 15

83 "5606 (MAP2K3)" ellipse s_6 ic Red fos 15

84 "5777 (PTPN6)" ellipse s_6 ic NavyBlue fos 15

85 "5806 (PTX3)" ellipse s_6 ic Red fos 15

86 "5837 (PYGM)" diamond s_10 ic White bw 10 fos 15

87 "5879 (RAC1)" diamond s_10 ic LightYellow bw 10 fos 15

88 "5932 (RBBP8)" ellipse s_6 ic Yellow fos 15

89 "5970 (RELA)" diamond s_10 ic Red bw 10 fos 15

90 "5971 (RELB)" diamond s_10 ic Red bw 10 fos 15

91 "6300 (MAPK12)" diamond s_10 ic White bw 10 fos 15

92 "6351 (CCL4)" ellipse s_6 ic Red fos 15

93 "6352 (CCL5)" ellipse s_6 ic Red fos 15

94 "6356 (CCL11)" ellipse s_6 ic Orange fos 15

95 "6364 (CCL20)" ellipse s_6 ic Red fos 15

96 "6373 (CXCL11)" ellipse s_6 ic Orange fos 15

97 "6385 (SDC4)" triangle s_10 ic Orange bw 10 fos 15

98 "6477 (SIAH1)" diamond s_10 ic Cyan bw 10 fos 15

99 "6507 (SLC1A3)" triangle s_10 ic Red bw 10 fos 15

100 "6517 (SLC2A4)" diamond s_10 ic Orange bw 10 fos 15

101 "6574 (SLC20A1)" ellipse s_6 ic NavyBlue fos 15

102 "6670 (SP3)" diamond s_10 ic Cyan bw 10 fos 15

103 "6714 (SRC)" ellipse s_6 ic Yellow fos 15

104 "6839 (SUV39H1)" ellipse s_6 ic NavyBlue fos 15

105 "6850 (SYK)" ellipse s_6 ic Blue fos 15

106 "6885 (MAP3K7)" ellipse s_6 ic LightCyan fos 15

107 "6890 (TAP1)" ellipse s_6 ic Red fos 15

108 "7033 (TFF3)" ellipse s_6 ic Orange fos 15

109 "7097 (TLR2)" triangle s_10 ic Red bw 10 fos 15

110 "7124 (TNF)" triangle s_10 ic Red bw 10 fos 15

111 "7132 (TNFRSF1A)" triangle s_10 ic Blue bw 10 fos 15

112 "7185 (TRAF1)" ellipse s_6 ic Red fos 15

113 "7187 (TRAF3)" ellipse s_6 ic Yellow fos 15

114 "7189 (TRAF6)" ellipse s_6 ic LightCyan fos 15

115 "7329 (UBE2I)" ellipse s_6 ic LightCyan fos 15

116 "7412 (VCAM1)" triangle s_10 ic Orange bw 10 fos 15

117 "7433 (VIPR1)" triangle s_10 ic Orange bw 10 fos 15

118 "7534 (YWHAZ)" ellipse s_6 ic LightYellow fos 15

119 "8312 (AXIN1)" ellipse s_6 ic Cyan fos 15

120 "8398 (PLA2G6)" diamond s_10 ic LightYellow bw 10 fos 15

121 "8517 (IKBKG)" ellipse s_6 ic White fos 15

122 "8737 (RIPK1)" ellipse s_6 ic Red fos 15

123 "8767 (RIPK2)" ellipse s_6 ic Red fos 15

124 "8772 (FADD)" ellipse s_6 ic Blue fos 15

125 "8795 (TNFRSF10B)" triangle s_10 ic Red bw 10 fos 15

126 "8797 (TNFRSF10A)" ellipse s_6 ic NavyBlue fos 15

127 "8870 (IER3)" ellipse s_6 ic Red fos 15

128 "9093 (DNAJA3)" ellipse s_6 ic NavyBlue fos 15

129 "9134 (CCNE2)" diamond s_10 ic Red bw 10 fos 15

130 "9184 (BUB3)" ellipse s_6 ic Cyan fos 15

131 "9308 (CD83)" triangle s_10 ic Red bw 10 fos 15

132 "9641 (IKBKE)" ellipse s_6 ic Red fos 15

133 "10125 (RASGRP1)" ellipse s_6 ic LightYellow fos 15

134 "10746 (MAP3K2)" ellipse s_6 ic White fos 15

135 "11261 (CHP)" ellipse s_6 ic Yellow fos 15

136 "11344 (TWF2)" ellipse s_6 ic NavyBlue fos 15

137 "23118 (MAP3K7IP2)" ellipse s_6 ic Orange fos 15

138 "23533 (PIK3R5)" ellipse s_6 ic Orange fos 15

139 "27289 (RND1)" diamond s_10 ic Red bw 10 fos 15

140 "29984 (RHOD)" ellipse s_6 ic LightCyan fos 15

141 "51135 (IRAK4)" ellipse s_6 ic Cyan fos 15

142 "56288 (PARD3)" ellipse s_6 ic LightYellow fos 15

143 "148022 (TICAM1)" ellipse s_6 ic Yellow fos 15

144 "353376 (TICAM2)" ellipse s_6 ic Yellow fos 15

*Arcs

79 78 1.0 ap 0 c Black

50 58 1.0 ap 0 c Black

48 49 1.0 ap 0 c Black

89 116 1.0 ap 0 c Lavender l TF-DNA

121 67 1.0 ap 0 c Black

102 34 1.0 ap 0 c Blue l TF-DNA

61 75 1.0 ap 0 c Black

135 79 1.0 ap 0 c Black

72 82 1.0 ap 0 c Black

72 57 1.0 ap 0 c Black

65 90 1.0 ap 0 c Purple l Both

65 128 1.0 ap 0 c Purple l Both

62 70 1.0 ap 0 c Black l TF-DNA

138 87 1.0 ap 0 c Black

65 44 1.0 ap 0 c Lavender l TF-DNA

41 137 0.6090037224768146 ap 0 c Black

65 22 1.0 ap 0 c Purple l Both

119 33 1.0 ap 0 c Black

54 40 0.6781178226079649 ap 0 c Black

72 80 1.0 ap 0 c Black

134 83 1.0 ap 0 c Black

89 64 1.0 ap 0 c Lavender l TF-DNA

65 51 1.0 ap 0 c Lavender l TF-DNA

57 75 1.0 ap 0 c Black

102 110 1.0 ap 0 c Blue l TF-DNA

82 142 1.0 ap 0 c Black

89 46 1.0 ap 0 c Lavender l TF-DNA

65 125 1.0 ap 0 c Lavender l TF-DNA

89 126 1.0 ap 0 c Blue l TF-DNA

59 62 1.0 ap 0 c Purple l Both

65 126 1.0 ap 0 c Blue l TF-DNA

89 48 1.0 ap 0 c Lavender l TF-DNA

36 74 1.0 ap 0 c Black

8 7 0.6820867698421389 ap 0 c Black

67 65 1.0 ap 0 c Black

9 37 1.0 ap 0 c Black

65 48 1.0 ap 0 c Lavender l TF-DNA

89 125 1.0 ap 0 c Lavender l TF-DNA

10 40 1.0 ap 0 c Black

75 81 0.630438026261182 ap 0 c Black

65 127 1.0 ap 0 c Lavender l TF-DNA

65 131 1.0 ap 0 c Lavender l TF-DNA

83 91 1.0 ap 0 c Black

110 111 1.0 ap 0 c Black

15 72 1.0 ap 0 c Black

71 2 1.0 ap 0 c Black

84 50 1.0 ap 0 c Black

58 72 1.0 ap 0 c Black

55 116 1.0 ap 0 c Lavender l TF-DNA

124 25 0.6539582681000604 ap 0 c Black

76 120 1.0 ap 0 c Black

65 117 1.0 ap 0 c Lavender l TF-DNA

74 10 1.0 ap 0 c Black

115 102 0.6528420726732054 ap 0 c Black

109 63 1.0 ap 0 c Black

63 141 1.0 ap 0 c Black

102 101 1.0 ap 0 c Lavender l TF-DNA

20 129 1.0 ap 0 c Black

113 132 1.0 ap 0 c Black

102 35 1.0 ap 0 c Blue l TF-DNA

65 16 1.0 ap 0 c Lavender l TF-DNA

130 8 1.0 ap 0 c Black

104 11 0.6180705731103185 ap 0 c Black

62 43 1.0 ap 0 c Black l TF-DNA

118 31 0.6794494213283769 ap 0 c Black

57 61 1.0 ap 0 c Black

52 58 1.0 ap 0 c Black

26 77 0.6487006226498353 ap 0 c Black

65 108 1.0 ap 0 c Lavender l TF-DNA

29 64 0.19122411567153658 ap 0 c Black

102 70 1.0 ap 0 c Blue l TF-DNA

89 1 1.0 ap 0 c Lavender l TF-DNA

65 73 1.0 ap 0 c Lavender l TF-DNA

65 46 1.0 ap 0 c Lavender l TF-DNA

89 18 1.0 ap 0 c Lavender l TF-DNA

133 40 1.0 ap 0 c Black

42 75 1.0 ap 0 c Black

65 116 1.0 ap 0 c Lavender l TF-DNA

65 19 1.0 ap 0 c Lavender l TF-DNA

89 53 1.0 ap 0 c Lavender l TF-DNA

51 52 1.0 ap 0 c Black

39 13 1.0 ap 0 c Black

44 45 1.0 ap 0 c Black

23 103 1.0 ap 0 c Black

111 122 1.0 ap 0 c Black

65 17 1.0 ap 0 c Lavender l TF-DNA

89 42 1.0 ap 0 c Lavender l TF-DNA

89 113 1.0 ap 0 c Lavender l TF-DNA

89 114 1.0 ap 0 c Black l TF-DNA

140 139 1.0 ap 0 c Black

87 65 1.0 ap 0 c Black

114 130 0.6537272048436442 ap 0 c Black

89 112 1.0 ap 0 c Lavender l TF-DNA

81 134 0.660072001313463 ap 0 c Black

135 9 1.0 ap 0 c Black

65 112 1.0 ap 0 c Lavender l TF-DNA

65 53 1.0 ap 0 c Lavender l TF-DNA

65 35 1.0 ap 0 c Lavender l TF-DNA

137 114 1.0 ap 0 c Black

65 18 1.0 ap 0 c Lavender l TF-DNA

65 59 1.0 ap 0 c Lavender l TF-DNA

63 124 1.0 ap 0 c Black

102 28 1.0 ap 0 c Lavender l TF-DNA

65 3 1.0 ap 0 c Lavender l TF-DNA

64 29 0.19122411567153658 ap 0 c Black

31 104 0.6697679278342372 ap 0 c Black

5 93 1.0 ap 0 c Lavender l TF-DNA

65 4 1.0 ap 0 c Lavender l TF-DNA

89 93 1.0 ap 0 c Lavender l TF-DNA

89 92 1.0 ap 0 c Lavender l TF-DNA

25 119 1.0 ap 0 c Black

125 124 1.0 ap 0 c Black

102 109 1.0 ap 0 c Blue l TF-DNA

65 39 1.0 ap 0 c Lavender l TF-DNA

46 47 1.0 ap 0 c Black

89 66 1.0 ap 0 c Purple l Both

19 14 0.6667488312172034 ap 0 c Black

56 23 1.0 ap 0 c Black

89 7 1.0 ap 0 c Black l TF-DNA

75 135 1.0 ap 0 c Black

65 55 1.0 ap 0 c Purple l Both

89 3 1.0 ap 0 c Lavender l TF-DNA

102 30 1.0 ap 0 c Lavender l TF-DNA

129 20 1.0 ap 0 c Black

89 95 1.0 ap 0 c Lavender l TF-DNA

89 35 1.0 ap 0 c Lavender l TF-DNA

65 110 1.0 ap 0 c Lavender l TF-DNA

65 107 1.0 ap 0 c Lavender l TF-DNA

55 17 1.0 ap 0 c Lavender l TF-DNA

14 72 1.0 ap 0 c Black

122 106 1.0 ap 0 c Black

27 88 0.6456182620846943 ap 0 c Black

75 133 1.0 ap 0 c Black

89 4 1.0 ap 0 c Lavender l TF-DNA

89 127 1.0 ap 0 c Lavender l TF-DNA

102 52 1.0 ap 0 c Blue l TF-DNA

62 20 1.0 ap 0 c Black l TF-DNA

102 51 1.0 ap 0 c Blue l TF-DNA

114 144 0.6511494958816968 ap 0 c Black

87 89 1.0 ap 0 c Black

62 32 1.0 ap 0 c Black l TF-DNA

61 84 0.6093167987368007 ap 0 c Black

49 58 1.0 ap 0 c Black

38 60 1.0 ap 0 c Black

1 36 1.0 ap 0 c Black

106 121 1.0 ap 0 c Black

47 63 1.0 ap 0 c Black

10 86 1.0 ap 0 c Black

90 17 1.0 ap 0 c Lavender l TF-DNA

78 10 1.0 ap 0 c Black

105 138 1.0 ap 0 c Black

132 115 0.6442101598603441 ap 0 c Black

75 61 1.0 ap 0 c Black

144 143 1.0 ap 0 c Black

143 113 1.0 ap 0 c Black

65 66 1.0 ap 0 c Purple l Both

124 63 0.674752024399367 ap 0 c Black

65 5 1.0 ap 0 c Purple l Both

100 2 0.15880354132184865 ap 0 c Black

102 99 1.0 ap 0 c Blue l TF-DNA

2 20 1.0 ap 0 c Black

62 21 1.0 ap 0 c Black l TF-DNA

102 1 1.0 ap 0 c Blue l TF-DNA

65 68 1.0 ap 0 c Lavender l TF-DNA

40 72 1.0 ap 0 c Black

88 98 0.6011579672210154 ap 0 c Black

45 58 1.0 ap 0 c Black

37 27 0.6098905157501813 ap 0 c Black

65 92 1.0 ap 0 c Lavender l TF-DNA

71 41 0.626371237931438 ap 0 c Black

65 93 1.0 ap 0 c Lavender l TF-DNA

13 38 1.0 ap 0 c Black

65 122 1.0 ap 0 c Lavender l TF-DNA

103 72 1.0 ap 0 c Black

65 94 1.0 ap 0 c Lavender l TF-DNA

89 51 1.0 ap 0 c Lavender l TF-DNA

65 29 1.0 ap 0 c Lavender l TF-DNA

62 64 1.0 ap 0 c Black l TF-DNA

142 62 0.6119875531361855 ap 0 c Black

116 56 1.0 ap 0 c Black

33 26 1.0 ap 0 c Black

65 69 1.0 ap 0 c Lavender l TF-DNA

65 24 1.0 ap 0 c Lavender l TF-DNA

141 114 1.0 ap 0 c Black

5 18 1.0 ap 0 c Lavender l TF-DNA

80 118 0.6492549217905756 ap 0 c Black

10 135 1.0 ap 0 c Black

61 76 1.0 ap 0 c Black

65 95 1.0 ap 0 c Lavender l TF-DNA

72 71 1.0 ap 0 c Black

60 84 1.0 ap 0 c Black

89 85 1.0 ap 0 c Lavender l TF-DNA

77 104 0.6237971163248115 ap 0 c Black

76 10 1.0 ap 0 c Black

89 44 1.0 ap 0 c Lavender l TF-DNA

84 105 1.0 ap 0 c Black

65 96 1.0 ap 0 c Lavender l TF-DNA

65 109 1.0 ap 0 c Lavender l TF-DNA

89 96 1.0 ap 0 c Lavender l TF-DNA

102 131 1.0 ap 0 c Blue l TF-DNA

102 21 1.0 ap 0 c Blue l TF-DNA

89 90 1.0 ap 0 c Purple l Both

16 15 1.0 ap 0 c Black

65 42 1.0 ap 0 c Lavender l TF-DNA

65 97 1.0 ap 0 c Lavender l TF-DNA

17 12 0.6102158566950838 ap 0 c Black

40 140 0.6051834770220744 ap 0 c Black

89 110 1.0 ap 0 c Lavender l TF-DNA

89 136 1.0 ap 0 c Blue l TF-DNA

89 6 1.0 ap 0 c Lavender l TF-DNA

124 54 0.6987593798324877 ap 0 c Black

2 100 0.15880354132184865 ap 0 c Black

65 123 1.0 ap 0 c Lavender l TF-DNA

1. 5 1.0 ap 0 c Lavender l TF-DNA

## The D2D network

*Vertices 314

1 "131 (ADH7)" ellipses_6 ic Orange fos 15

2 "161 (AP2A2)" ellipses_6 ic Blue fos 15

3 "185 (AGTR1)" triangles_10 ic Orange bw 10 fos 15

4 "196 (AHR)" ellipses_6 ic NavyBlue fos 15

5 "273 (AMPH)" ellipses_6 ic Blue fos 15

6 "317 (APAF1)" ellipses_6 ic LightCyan fos 15

7 "329 (BIRC2)" ellipses_6 ic Orange fos 15

8 "330 (BIRC3)" ellipses_6 ic Red fos 15

9 "467 (ATF3)" diamonds_10 ic Red bw 10 fos 15

10 "468 (ATF4)" diamonds_10 ic Orange bw 10 fos 15

11 "481 (ATP1B1)" triangles_10 ic Red bw 10 fos 15

12 "545 (ATR)" triangles_10 ic NavyBlue bw 10 fos 15

13 "597 (BCL2A1)" ellipses_6 ic Red fos 15

14 "602 (BCL3)" diamonds_10 ic Orange bw 10 fos 15

15 "605 (BCL7A)" ellipses_6 ic Blue fos 15

16 "637 (BID)" ellipses_6 ic Red fos 15

17 "694 (BTG1)" ellipses_6 ic Red fos 15

18 "941 (CD80)" triangles_10 ic Red bw 10 fos 15

19 "958 (CD40)" triangles_10 ic Red bw 10 fos 15

20 "960 (CD44)" triangles_10 ic Red bw 10 fos 15

21 "965 (CD58)" triangles_10 ic Red bw 10 fos 15

22 "969 (CD69)" triangles_10 ic Red bw 10 fos 15

23 "973 (CD79A)" triangles_10 ic Orange bw 10 fos 15

24 "994 (CDC25B)" ellipses_6 ic Blue fos 15

25 "1027 (CDKN1B)" diamonds_10 ic Blue bw 10 fos 15

26 "1050 (CEBPA)" diamonds_10 ic Blue bw 10 fos 15

27 "1103 (CHAT)" ellipses_6 ic Orange fos 15

28 "1230 (CCR1)" triangles_10 ic Blue bw 10 fos 15

29 "1231 (CCR2)" ellipses_6 ic Blue fos 15

30 "1326 (MAP3K8)" ellipses_6 ic Red fos 15

31 "1447 (CSN2)" ellipses_6 ic Orange fos 15

32 "1503 (CTPS)" ellipses_6 ic Blue fos 15

33 "1581 (CYP7A1)" ellipses_6 ic Orange fos 15

34 "1628 (DBP)" diamonds_10 ic LightCyan bw 10 fos 15

35 "1647 (GADD45A)" diamonds_10 ic Red bw 10 fos 15

36 "1786 (DNMT1)" ellipses_6 ic NavyBlue fos 15

37 "1796 (DOK1)" ellipses_6 ic Blue fos 15

38 "1912 (PHC2)" ellipses_6 ic LightCyan fos 15

39 "1958 (EGR1)" diamonds_10 ic Orange bw 10 fos 15

40 "1959 (EGR2)" diamonds_10 ic NavyBlue bw 10 fos 15

41 "1960 (EGR3)" diamonds_10 ic Red bw 10 fos 15

42 "2071 (ERCC3)" ellipses_6 ic NavyBlue fos 15

43 "2107 (ETF1)" ellipses_6 ic Orange fos 15

44 "2114 (ETS2)" diamonds_10 ic Orange bw 10 fos 15

45 "2146 (EZH2)" ellipses_6 ic Red fos 15

46 "2152 (F3)" ellipses_6 ic Red fos 15

47 "2158 (F9)" ellipses_6 ic Orange fos 15

48 "2159 (F10)" ellipses_6 ic Orange fos 15

49 "2178 (FANCE)" ellipses_6 ic Blue fos 15

50 "2188 (FANCF)" ellipses_6 ic Blue fos 15

51 "2189 (FANCG)" ellipses_6 ic NavyBlue fos 15

52 "2242 (FES)" ellipses_6 ic Blue fos 15

53 "2274 (FHL2)" diamonds_10 ic Red bw 10 fos 15

54 "2275 (FHL3)" ellipses_6 ic NavyBlue fos 15

55 "2535 (FZD2)" triangles_10 ic Blue bw 10 fos 15

56 "2627 (GATA6)" ellipses_6 ic Orange fos 15

57 "2634 (GBP2)" ellipses_6 ic Red fos 15

58 "2643 (GCH1)" ellipses_6 ic Red fos 15

59 "2745 (GLRX)" ellipses_6 ic Red fos 15

60 "2959 (GTF2B)" diamonds_10 ic Red bw 10 fos 15

61 "2960 (GTF2E1)" ellipses_6 ic NavyBlue fos 15

62 "2984 (GUCY2C)" triangles_10 ic Orange bw 10 fos 15

63 "3087 (HHEX)" ellipses_6 ic Blue fos 15

64 "3096 (HIVEP1)" ellipses_6 ic Orange fos 15

65 "3105 (HLA-A)" ellipses_6 ic Orange fos 15

66 "3106 (HLA-B)" ellipses_6 ic Orange fos 15

67 "3134 (HLA-F)" triangles_10 ic Red bw 10 fos 15

68 "3135 (HLA-G)" ellipses_6 ic Orange fos 15

69 "3183 (HNRNPC)" ellipses_6 ic Yellow fos 15

70 "3269 (HRH1)" triangles_10 ic Blue bw 10 fos 15

71 "3301 (DNAJA1)" ellipses_6 ic Red fos 15

72 "3303 (HSPA1A)" ellipses_6 ic Orange fos 15

73 "3305 (HSPA1L)" ellipses_6 ic Blue fos 15

74 "3383 (ICAM1)" triangles_10 ic Red bw 10 fos 15

75 "3394 (IRF8)" ellipses_6 ic Red fos 15

76 "3399 (ID3)" ellipses_6 ic Blue fos 15

77 "3433 (IFIT2)" ellipses_6 ic Red fos 15

78 "3434 (IFIT1)" ellipses_6 ic Red fos 15

79 "3437 (IFIT3)" ellipses_6 ic Red fos 15

80 "3456 (IFNB1)" triangles_10 ic Red bw 10 fos 15

81 "3553 (IL1B)" triangles_10 ic Red bw 10 fos 15

82 "3556 (IL1RAP)" triangles_10 ic Red bw 10 fos 15

83 "3561 (IL2RG)" ellipses_6 ic Orange fos 15

84 "3569 (IL6)" triangles_10 ic Red bw 10 fos 15

85 "3577 (IL8RA)" triangles_10 ic Blue bw 10 fos 15

86 "3579 (IL8RB)" ellipses_6 ic Blue fos 15

87 "3587 (IL10RA)" ellipses_6 ic Red fos 15

88 "3588 (IL10RB)" triangles_10 ic NavyBlue bw 10 fos 15

89 "3593 (IL12B)" triangles_10 ic Red bw 10 fos 15

90 "3595 (IL12RB2)" ellipses_6 ic Red fos 15

91 "3603 (IL16)" ellipses_6 ic LightCyan fos 15

92 "3615 (IMPDH2)" ellipses_6 ic White fos 15

93 "3627 (CXCL10)" ellipses_6 ic Red fos 15

94 "3659 (IRF1)" diamonds_10 ic Red bw 10 fos 15

95 "3699 (ITIH3)" ellipses_6 ic Orange fos 15

96 "3726 (JUNB)" diamonds_10 ic Red bw 10 fos 15

97 "3759 (KCNJ2)" ellipses_6 ic Red fos 15

98 "3856 (KRT8)" ellipses_6 ic Orange fos 15

99 "3936 (LCP1)" ellipses_6 ic Orange fos 15

100 "3937 (LCP2)" ellipses_6 ic Red fos 15

101 "3952 (LEP)" triangles_10 ic Blue bw 10 fos 15

102 "4066 (LYL1)" ellipses_6 ic Blue fos 15

103 "4090 (SMAD5)" diamonds_10 ic Blue bw 10 fos 15

104 "4188 (MDFI)" ellipses_6 ic Orange fos 15

105 "4215 (MAP3K3)" triangles_10 ic NavyBlue bw 10 fos 15

106 "4217 (MAP3K5)" ellipses_6 ic Blue fos 15

107 "4221 (MEN1)" triangles_10 ic NavyBlue bw 10 fos 15

108 "4283 (CXCL9)" ellipses_6 ic Red fos 15

109 "4356 (MPP3)" ellipses_6 ic Orange fos 15

110 "4435 (CITED1)" ellipses_6 ic Red fos 15

111 "4486 (MST1R)" triangles_10 ic Orange bw 10 fos 15

112 "4616 (GADD45B)" diamonds_10 ic Orange bw 10 fos 15

113 "4664 (NAB1)" ellipses_6 ic Orange fos 15

114 "4778 (NFE2)" diamonds_10 ic Blue bw 10 fos 15

115 "4790 (NFKB1)" diamonds_10 ic Red bw 10 fos 15

116 "4792 (NFKBIA)" diamonds_10 ic Red bw 10 fos 15

117 "4920 (ROR2)" ellipses_6 ic Orange fos 15

118 "4929 (NR4A2)" ellipses_6 ic Orange fos 15

119 "4939 (OAS2)" ellipses_6 ic Orange fos 15

120 "5054 (SERPINE1)" diamonds_10 ic Red bw 10 fos 15

121 "5295 (PIK3R1)" ellipses_6 ic Blue fos 15

122 "5329 (PLAUR)" ellipses_6 ic Orange fos 15

123 "5366 (PMAIP1)" ellipses_6 ic Red fos 15

124 "5437 (POLR2H)" ellipses_6 ic NavyBlue fos 15

125 "5552 (SRGN)" ellipses_6 ic Red fos 15

126 "5606 (MAP2K3)" ellipses_6 ic Red fos 15

127 "5687 (PSMA6)" ellipses_6 ic Red fos 15

128 "5777 (PTPN6)" ellipses_6 ic NavyBlue fos 15

129 "5806 (PTX3)" ellipses_6 ic Red fos 15

130 "5927 (JARID1A)" ellipses_6 ic NavyBlue fos 15

131 "5970 (RELA)" diamonds_10 ic Red bw 10 fos 15

132 "5971 (RELB)" diamonds_10 ic Red bw 10 fos 15

133 "5977 (DPF2)" ellipses_6 ic Blue fos 15

134 "5987 (TRIM27)" ellipses_6 ic Blue fos 15

135 "6047 (RNF4)" ellipses_6 ic NavyBlue fos 15

136 "6195 (RPS6KA1)" ellipses_6 ic NavyBlue fos 15

137 "6311 (ATXN2)" ellipses_6 ic NavyBlue fos 15

138 "6351 (CCL4)" ellipses_6 ic Red fos 15

139 "6352 (CCL5)" ellipses_6 ic Red fos 15

140 "6355 (CCL8)" ellipses_6 ic Red fos 15

141 "6356 (CCL11)" ellipses_6 ic Orange fos 15

142 "6360 (CCL16)" ellipses_6 ic Red fos 15

143 "6364 (CCL20)" ellipses_6 ic Red fos 15

144 "6373 (CXCL11)" ellipses_6 ic Orange fos 15

145 "6385 (SDC4)" triangles_10 ic Orange bw 10 fos 15

146 "6389 (SDHA)" ellipses_6 ic Blue fos 15

147 "6447 (SCG5)" ellipses_6 ic Orange fos 15

148 "6523 (SLC5A1)" ellipses_6 ic Orange fos 15

149 "6603 (SMARCD2)" ellipses_6 ic NavyBlue fos 15

150 "6617 (SNAPC1)" ellipses_6 ic Blue fos 15

151 "6624 (FSCN1)" ellipses_6 ic Red fos 15

152 "6837 (MED22)" ellipses_6 ic NavyBlue fos 15

153 "6839 (SUV39H1)" ellipses_6 ic NavyBlue fos 15

154 "6850 (SYK)" ellipses_6 ic Blue fos 15

155 "6877 (TAF5)" ellipses_6 ic Blue fos 15

156 "6890 (TAP1)" ellipses_6 ic Red fos 15

157 "6908 (TBP)" ellipses_6 ic NavyBlue fos 15

158 "7033 (TFF3)" ellipses_6 ic Orange fos 15

159 "7097 (TLR2)" triangles_10 ic Red bw 10 fos 15

160 "7124 (TNF)" triangles_10 ic Red bw 10 fos 15

161 "7128 (TNFAIP3)" ellipses_6 ic Red fos 15

162 "7132 (TNFRSF1A)" triangles_10 ic Blue bw 10 fos 15

163 "7159 (TP53BP2)" ellipses_6 ic Red fos 15

164 "7185 (TRAF1)" ellipses_6 ic Red fos 15

165 "7203 (CCT3)" ellipses_6 ic NavyBlue fos 15

166 "7412 (VCAM1)" triangles_10 ic Orange bw 10 fos 15

167 "7433 (VIPR1)" triangles_10 ic Orange bw 10 fos 15

168 "7469 (WHSC2)" ellipses_6 ic Blue fos 15

169 "7472 (WNT2)" triangles_10 ic Red bw 10 fos 15

170 "7474 (WNT5A)" triangles_10 ic Red bw 10 fos 15

171 "7533 (YWHAH)" ellipses_6 ic NavyBlue fos 15

172 "7568 (ZNF20)" ellipses_6 ic Blue fos 15

173 "7629 (ZNF76)" ellipses_6 ic NavyBlue fos 15

174 "7727 (ZNF174)" ellipses_6 ic NavyBlue fos 15

175 "7832 (BTG2)" ellipses_6 ic Red fos 15

176 "7884 (SLBP)" ellipses_6 ic NavyBlue fos 15

177 "8013 (NR4A3)" ellipses_6 ic Red fos 15

178 "8161 (COIL)" ellipses_6 ic NavyBlue fos 15

179 "8266 (UBL4A)" ellipses_6 ic NavyBlue fos 15

180 "8321 (FZD1)" triangles_10 ic Red bw 10 fos 15

181 "8405 (SPOP)" ellipses_6 ic Blue fos 15

182 "8660 (IRS2)" diamonds_10 ic NavyBlue bw 10 fos 15

183 "8676 (STX11)" ellipses_6 ic Red fos 15

184 "8737 (RIPK1)" ellipses_6 ic Red fos 15

185 "8767 (RIPK2)" ellipses_6 ic Red fos 15

186 "8772 (FADD)" ellipses_6 ic Blue fos 15

187 "8795 (TNFRSF10B)" triangles_10 ic Red bw 10 fos 15

188 "8797 (TNFRSF10A)" ellipses_6 ic NavyBlue fos 15

189 "8809 (IL18R1)" ellipses_6 ic Red fos 15

190 "8834 (TMEM11)" ellipses_6 ic NavyBlue fos 15

191 "8837 (CFLAR)" ellipses_6 ic Red fos 15

192 "8870 (IER3)" ellipses_6 ic Red fos 15

193 "8930 (MBD4)" ellipses_6 ic Blue fos 15

194 "9015 (TAF1A)" ellipses_6 ic NavyBlue fos 15

195 "9046 (DOK2)" ellipses_6 ic Blue fos 15

196 "9051 (PSTPIP1)" ellipses_6 ic NavyBlue fos 15

197 "9093 (DNAJA3)" ellipses_6 ic NavyBlue fos 15

198 "9134 (CCNE2)" diamonds_10 ic Red bw 10 fos 15

199 "9308 (CD83)" triangles_10 ic Red bw 10 fos 15

200 "9337 (CNOT8)" ellipses_6 ic NavyBlue fos 15

201 "9456 (HOMER1)" ellipses_6 ic Orange fos 15

202 "9519 (TBPL1)" ellipses_6 ic NavyBlue fos 15

203 "9530 (BAG4)" ellipses_6 ic Blue fos 15

204 "9641 (IKBKE)" ellipses_6 ic Red fos 15

205 "9655 (SOCS5)" ellipses_6 ic Blue fos 15

206 "9775 (EIF4A3)" ellipses_6 ic NavyBlue fos 15

207 "9817 (KEAP1)" ellipses_6 ic Blue fos 15

208 "9868 (TOMM70A)" ellipses_6 ic Blue fos 15

209 "9997 (SCO2)" ellipses_6 ic Orange fos 15

210 "10127 (ZNF263)" ellipses_6 ic NavyBlue fos 15

211 "10199 (MPHOSPH10)" ellipses_6 ic NavyBlue fos 15

212 "10318 (TNIP1)" ellipses_6 ic Red fos 15

213 "10333 (TLR6)" triangles_10 ic NavyBlue bw 10 fos 15

214 "10392 (NOD1)" ellipses_6 ic Blue fos 15

215 "10436 (EMG1)" ellipses_6 ic NavyBlue fos 15

216 "10475 (TRIM38)" ellipses_6 ic Blue fos 15

217 "10557 (RPP38)" ellipses_6 ic Blue fos 15

218 "10799 (RPP40)" ellipses_6 ic Blue fos 15

219 "10808 (HSPH1)" ellipses_6 ic Blue fos 15

220 "10817 (FRS3)" ellipses_6 ic NavyBlue fos 15

221 "10845 (CLPX)" ellipses_6 ic Blue fos 15

222 "10849 (CD3EAP)" ellipses_6 ic NavyBlue fos 15

223 "10910 (SUGT1)" ellipses_6 ic NavyBlue fos 15

224 "10989 (IMMT)" ellipses_6 ic NavyBlue fos 15

225 "11080 (DNAJB4)" ellipses_6 ic Red fos 15

226 "11102 (RPP14)" ellipses_6 ic NavyBlue fos 15

227 "11140 (CDC37)" ellipses_6 ic Red fos 15

228 "11143 (MYST2)" ellipses_6 ic Blue fos 15

229 "11156 (PTP4A3)" ellipses_6 ic Red fos 15

230 "11162 (NUDT6)" ellipses_6 ic NavyBlue fos 15

231 "11164 (NUDT5)" ellipses_6 ic NavyBlue fos 15

232 "11200 (CHEK2)" ellipses_6 ic Blue fos 15

233 "11218 (DDX20)" ellipses_6 ic NavyBlue fos 15

234 "11344 (TWF2)" ellipses_6 ic NavyBlue fos 15

235 "22841 (RAB11FIP2)" ellipses_6 ic NavyBlue fos 15

236 "22955 (SCMH1)" ellipses_6 ic Orange fos 15

237 "22984 (PDCD11)" ellipses_6 ic NavyBlue fos 15

238 "23011 (RAB21)" ellipses_6 ic Red fos 15

239 "23020 (ASCC3L1)" ellipses_6 ic Blue fos 15

240 "23118 (MAP3K7IP2)" ellipses_6 ic Orange fos 15

241 "23424 (TDRD7)" ellipses_6 ic Orange fos 15

242 "23533 (PIK3R5)" ellipses_6 ic Orange fos 15

243 "23645 (PPP1R15A)" ellipses_6 ic Red fos 15

244 "23659 (LYPLA3)" ellipses_6 ic NavyBlue fos 15

245 "23673 (STX12)" ellipses_6 ic Orange fos 15

246 "23708 (GSPT2)" ellipses_6 ic Blue fos 15

247 "23764 (MAFF)" diamonds_10 ic Red bw 10 fos 15

248 "25888 (ZNF473)" ellipses_6 ic Blue fos 15

249 "25920 (RP13-122B23.3)" ellipses_6 ic NavyBlue fos 15

250 "25929 (GEMIN5)" ellipses_6 ic Blue fos 15

251 "26122 (EPC2)" ellipses_6 ic NavyBlue fos 15

252 "26505 (CNNM3)" ellipses_6 ic NavyBlue fos 15

253 "27289 (RND1)" diamonds_10 ic Red bw 10 fos 15

254 "28512 (NKIRAS1)" ellipses_6 ic Red fos 15

255 "29760 (BLNK)" triangles_10 ic Blue bw 10 fos 15

256 "51053 (GMNN)" ellipses_6 ic Blue fos 15

257 "51076 (CUTC)" ellipses_6 ic NavyBlue fos 15

258 "51147 (ING4)" ellipses_6 ic NavyBlue fos 15

259 "51367 (POP5)" ellipses_6 ic NavyBlue fos 15

260 "51499 (TRIAP1)" ellipses_6 ic Blue fos 15

261 "51537 (MTP18)" ellipses_6 ic NavyBlue fos 15

262 "51545 (ZNF581)" ellipses_6 ic Blue fos 15

263 "51562 (MBIP)" ellipses_6 ic NavyBlue fos 15

264 "54407 (SLC38A2)" triangles_10 ic NavyBlue bw 10 fos 15

265 "54512 (EXOSC4)" ellipses_6 ic NavyBlue fos 15

266 "54700 (RRN3)" ellipses_6 ic Orange fos 15

267 "54913 (RPP25)" ellipses_6 ic NavyBlue fos 15

268 "54977 (SLC25A38)" ellipses_6 ic Blue fos 15

269 "55068 (ENOX1)" ellipses_6 ic Red fos 15

270 "55257 (C20orf20)" ellipses_6 ic NavyBlue fos 15

271 "55272 (IMP3)" ellipses_6 ic Blue fos 15

272 "55290 (BRF2)" ellipses_6 ic NavyBlue fos 15

273 "55324 (ABCF3)" ellipses_6 ic NavyBlue fos 15

274 "55367 (LRDD)" ellipses_6 ic NavyBlue fos 15

275 "55643 (BTBD2)" ellipses_6 ic Orange fos 15

276 "55664 (CDC37L1)" ellipses_6 ic NavyBlue fos 15

277 "55922 (NKRF)" ellipses_6 ic NavyBlue fos 15

278 "55929 (DMAP1)" ellipses_6 ic NavyBlue fos 15

279 "56896 (DPYSL5)" ellipses_6 ic Orange fos 15

280 "60491 (NIF3L1)" ellipses_6 ic Blue fos 15

281 "64210 (MMS19)" ellipses_6 ic NavyBlue fos 15

282 "64320 (RNF25)" ellipses_6 ic Blue fos 15

283 "64745 (METT11D1)" ellipses_6 ic NavyBlue fos 15

284 "64965 (MRPS9)" ellipses_6 ic Blue fos 15

285 "79155 (TNIP2)" ellipses_6 ic Red fos 15

286 "79159 (NOL12)" ellipses_6 ic NavyBlue fos 15

287 "79666 (PLEKHF2)" ellipses_6 ic Red fos 15

288 "79686 (C14orf139)" ellipses_6 ic Red fos 15

289 "79833 (GEMIN6)" ellipses_6 ic Blue fos 15

290 "79902 (NUP85)" ellipses_6 ic Blue fos 15

291 "79959 (CEP76)" ellipses_6 ic Blue fos 15

292 "80345 (ZSCAN16)" ellipses_6 ic Blue fos 15

293 "83759 (RBM4B)" ellipses_6 ic NavyBlue fos 15

294 "84078 (KBTBD7)" ellipses_6 ic Blue fos 15

295 "84080 (C16orf48)" ellipses_6 ic NavyBlue fos 15

296 "84106 (PRAM1)" ellipses_6 ic Blue fos 15

297 "84273 (C4orf14)" ellipses_6 ic Blue fos 15

298 "84446 (BRSK1)" ellipses_6 ic Orange fos 15

299 "84527 (ZNF559)" ellipses_6 ic NavyBlue fos 15

300 "90594 (ZNF439)" ellipses_6 ic NavyBlue fos 15

301 "92595 (ZNF764)" ellipses_6 ic Blue fos 15

302 "112950 (MED8)" ellipses_6 ic NavyBlue fos 15

303 "116143 (WDR92)" ellipses_6 ic Blue fos 15

304 "118788 (PIK3AP1)" ellipses_6 ic Red fos 15

305 "124923 (FLJ25006)" ellipses_6 ic Blue fos 15

306 "134728 (IRAK1BP1)" ellipses_6 ic NavyBlue fos 15

307 "140885 (SIRPA)" triangles_10 ic Red bw 10 fos 15

308 "149420 (PDIK1L)" ellipses_6 ic Blue fos 15

309 "196513 (DCP1B)" ellipses_6 ic Blue fos 15

310 "282616 (IL28A)" triangles_10 ic Orange bw 10 fos 15

311 "282618 (IL29)" triangles_10 ic Red bw 10 fos 15

312 "283464 (GLT8D3)" ellipses_6 ic Blue fos 15

313 "339230 (CCDC137)" ellipses_6 ic NavyBlue fos 15

314 "494514 (C18orf56)" ellipses_6 ic Red fos 15

*Arcs

236 38 0.4342233333182914 ap 0 c Black

303 105 0.6346846155762462 ap 0 c Black

132 115 0.5421148828035831 ap 0 c Black

132 116 0.5362195273457306 ap 0 c Black

57 275 0.381908126053177 ap 0 c Black

204 116 0.5546006240038455 ap 0 c Black

106 59 0.44705573624029277 ap 0 c Black

8 164 0.6345072259226064 ap 0 c Black

72 208 0.27559550410241757 ap 0 c Black

104 295 0.1769730085325099 ap 0 c Black

60 202 0.23769732745013633 ap 0 c Black

26 89 1.0 ap 0 c Blue l TF-DNA

115 73 0.38912356889206706 ap 0 c Black

12 232 1.0 ap 0 c Black

115 72 0.42054649846867037 ap 0 c Black

39 120 1.0 ap 0 c Lavender l TF-DNA

28 140 0.37067877393285364 ap 0 c Black

115 71 0.5641625514920002 ap 0 c Black

28 139 0.36156212200445986 ap 0 c Black

115 197 1.0 ap 0 c Purple l Both

28 138 0.3222824047342254 ap 0 c Black

277 115 0.19980341355612483 ap 0 c Black

184 216 0.3137423808299148 ap 0 c Black

217 259 0.29117003906834915 ap 0 c Black

72 184 0.16268153046528963 ap 0 c Black

14 115 0.4848062288616039 ap 0 c Black

156 104 0.1561691629509758 ap 0 c Black

279 52 0.11592789460327077 ap 0 c Black

73 30 0.37694984811917964 ap 0 c Black

164 8 0.6345072259226064 ap 0 c Black

151 191 0.4131767740128001 ap 0 c Black

115 14 0.4848062288616039 ap 0 c Black

191 227 0.5802990291435908 ap 0 c Black

7 185 0.6496917621709739 ap 0 c Black

30 72 0.42640401746212525 ap 0 c Black

44 96 1.0 ap 0 c Lavender l TF-DNA

115 89 1.0 ap 0 c Lavender l TF-DNA

2 5 0.20062341419201196 ap 0 c Black

106 126 1.0 ap 0 c Black

174 172 0.47372835680009456 ap 0 c Black

52 279 0.11592789460327077 ap 0 c Black

30 73 0.37694984811917964 ap 0 c Black

164 7 0.5219330324636395 ap 0 c Black

285 161 0.5310431068084502 ap 0 c Black

184 274 0.21239169222143076 ap 0 c Black

38 236 0.4342233333182914 ap 0 c Black

161 164 0.43353014708444765 ap 0 c Black

285 30 0.49923100120929886 ap 0 c Black

115 84 1.0 ap 0 c Lavender l TF-DNA

269 280 0.21766653541810854 ap 0 c Black

231 204 0.298717902564725 ap 0 c Black

124 204 0.29415347253195406 ap 0 c Black

114 247 0.38621277478940275 ap 0 c Black

136 116 0.5736432719910762 ap 0 c Black

236 256 0.395842005538379 ap 0 c Black

110 115 0.13707816654328459 ap 0 c Black

71 162 0.4359985311201446 ap 0 c Black

285 115 0.5559333486376676 ap 0 c Black

115 199 1.0 ap 0 c Lavender l TF-DNA

97 91 0.23239944683244448 ap 0 c Black

115 212 0.5176701623436856 ap 0 c Black

187 191 0.3538054910003756 ap 0 c Black

100 255 0.45482486486005386 ap 0 c Black

78 79 0.29919714886718923 ap 0 c Black

103 107 0.629935707997234 ap 0 c Black

40 233 1.0 ap 0 c Lavender l TF-DNA

52 37 0.49085808144580906 ap 0 c Black

28 142 0.2529135833271356 ap 0 c Black

207 43 0.30951132781302043 ap 0 c Black

36 278 0.5989128057669467 ap 0 c Black

117 55 0.14851934051546847 ap 0 c Black

237 115 0.5668204543471446 ap 0 c Black

185 19 0.42818824250392745 ap 0 c Black

132 149 0.3787178710856305 ap 0 c Black

21 71 0.49823358034928855 ap 0 c Black

52 121 0.44084114598668533 ap 0 c Black

297 273 0.479611348047747 ap 0 c Black

131 237 0.4886342166303141 ap 0 c Black

185 127 0.6580840863874308 ap 0 c Black

66 146 0.2927029514568834 ap 0 c Black

109 297 0.35204170646774474 ap 0 c Black

205 87 1.0 ap 0 c Black

233 289 0.6354893497110092 ap 0 c Black

205 88 1.0 ap 0 c Black

227 204 0.6358532216505587 ap 0 c Black

60 157 0.2935093096551368 ap 0 c Black

313 109 0.36332829735430566 ap 0 c Black

26 84 1.0 ap 0 c Blue l TF-DNA

131 94 0.42965868187398204 ap 0 c Black

221 229 0.47903427010411037 ap 0 c Black

259 226 0.6007292980494991 ap 0 c Black

287 263 0.61239161461075 ap 0 c Black

111 121 0.36280172536363314 ap 0 c Black

61 42 0.5014121405147607 ap 0 c Black

205 90 1.0 ap 0 c Black

73 105 0.4393933987810719 ap 0 c Black

115 81 1.0 ap 0 c Lavender l TF-DNA

232 24 1.0 ap 0 c Black

116 136 0.5736432719910762 ap 0 c Black

131 93 1.0 ap 0 c Lavender l TF-DNA

308 160 0.2487352328100839 ap 0 c Black

36 134 0.6850778896568891 ap 0 c Black

104 274 0.17137941147767596 ap 0 c Black

115 110 0.13707816654328459 ap 0 c Black

26 1 1.0 ap 0 c Blue l TF-DNA

252 104 0.19103658605473714 ap 0 c Black

11 58 0.252318395647981 ap 0 c Black

115 96 1.0 ap 0 c Lavender l TF-DNA

261 272 0.3979153316136415 ap 0 c Black

115 7 1.0 ap 0 c Lavender l TF-DNA

36 153 0.6697679278342372 ap 0 c Black

146 204 0.33984329201410185 ap 0 c Black

115 8 1.0 ap 0 c Lavender l TF-DNA

311 88 1.0 ap 0 c Black

157 173 0.4426929323252815 ap 0 c Black

65 68 0.49994178291597663 ap 0 c Black

288 294 0.18141426272176422 ap 0 c Black

256 236 0.395842005538379 ap 0 c Black

186 274 0.516999887872986 ap 0 c Black

131 9 0.29940570499703645 ap 0 c Black

195 191 0.3806836101608239 ap 0 c Black

116 121 0.4345699894750008 ap 0 c Black

81 82 1.0 ap 0 c Black

173 157 0.4426929323252815 ap 0 c Black

131 116 1.0 ap 0 c Purple l Both

131 115 0.42820479349937685 ap 0 c Black

66 127 0.395484422967996 ap 0 c Black

58 11 0.252318395647981 ap 0 c Black

198 25 1.0 ap 0 c Black

136 118 0.40301000097215234 ap 0 c Black

10 243 1.0 ap 0 c Lavender l TF-DNA

131 282 0.628397503161461 ap 0 c Black

99 137 0.3989896576490773 ap 0 c Black

233 229 0.39832172988096476 ap 0 c Black

238 66 0.2875489505036219 ap 0 c Black

304 121 0.4025728077987404 ap 0 c Black

131 157 0.4533532462849016 ap 0 c Black

94 19 1.0 ap 0 c Lavender l TF-DNA

239 204 0.46229888231925276 ap 0 c Black

152 302 0.6314700569183479 ap 0 c Black

182 128 0.3644278115835904 ap 0 c Black

13 16 0.4703063612892095 ap 0 c Black

23 128 0.4206587271597029 ap 0 c Black

274 104 0.17137941147767596 ap 0 c Black

72 164 0.4136695836456755 ap 0 c Black

197 72 0.46399560301119946 ap 0 c Black

138 28 0.3222824047342254 ap 0 c Black

132 19 1.0 ap 0 c Lavender l TF-DNA

71 105 0.6045054390859078 ap 0 c Black

75 94 0.5717189335837831 ap 0 c Black

72 116 0.546282520573828 ap 0 c Black

220 253 0.3535069892065875 ap 0 c Black

271 211 0.295866509811291 ap 0 c Black

72 115 0.42054649846867037 ap 0 c Black

121 128 0.3616975618647896 ap 0 c Black

217 226 0.29337336194627706 ap 0 c Black

69 204 0.6402178536530986 ap 0 c Black

204 146 0.33984329201410185 ap 0 c Black

186 191 0.5027584480562084 ap 0 c Black

128 307 0.2920439650638414 ap 0 c Black

171 24 1.0 ap 0 c Black

147 196 0.35706425105806827 ap 0 c Black

115 138 1.0 ap 0 c Lavender l TF-DNA

115 139 1.0 ap 0 c Lavender l TF-DNA

115 141 1.0 ap 0 c Lavender l TF-DNA

134 36 0.6850778896568891 ap 0 c Black

73 309 0.5441838786524129 ap 0 c Black

73 270 0.5091824297403772 ap 0 c Black

236 190 0.42848324609013 ap 0 c Black

252 229 0.3785999063598686 ap 0 c Black

36 45 0.6814339244948303 ap 0 c Black

8 19 0.44651898773735743 ap 0 c Black

278 36 0.5989128057669467 ap 0 c Black

115 143 1.0 ap 0 c Lavender l TF-DNA

65 66 0.5277689717630464 ap 0 c Black

289 233 0.6354893497110092 ap 0 c Black

72 185 0.44779806101477204 ap 0 c Black

19 8 0.44651898773735743 ap 0 c Black

246 43 0.28189894915942904 ap 0 c Black

131 129 1.0 ap 0 c Lavender l TF-DNA

124 19 0.25079227562239914 ap 0 c Black

123 13 0.45183382828324836 ap 0 c Black

9 96 0.29033010754979977 ap 0 c Black

265 66 0.3723066536422219 ap 0 c Black

229 281 0.39930239621876523 ap 0 c Black

115 144 1.0 ap 0 c Lavender l TF-DNA

74 83 0.48156683390526334 ap 0 c Black

305 196 0.41376557453072765 ap 0 c Black

23 154 0.41625641335194513 ap 0 c Black

115 159 1.0 ap 0 c Lavender l TF-DNA

195 181 0.30334726516551574 ap 0 c Black

9 10 0.5169533390172593 ap 0 c Black

104 252 0.19103658605473714 ap 0 c Black

72 243 0.5352024943880083 ap 0 c Black

105 197 0.5835954660184537 ap 0 c Black

262 104 0.20733168448739422 ap 0 c Black

247 114 0.38621277478940275 ap 0 c Black

19 7 0.42115976186096443 ap 0 c Black

206 69 0.6872857074204007 ap 0 c Black

52 182 0.38825167904390245 ap 0 c Black

146 66 0.2927029514568834 ap 0 c Black

115 145 1.0 ap 0 c Lavender l TF-DNA

131 160 1.0 ap 0 c Lavender l TF-DNA

26 33 1.0 ap 0 c Blue l TF-DNA

71 164 0.3708991815483336 ap 0 c Black

82 186 1.0 ap 0 c Black

105 106 0.4681738462002579 ap 0 c Black

130 157 0.4419027011115432 ap 0 c Black

282 131 0.628397503161461 ap 0 c Black

94 131 0.42965868187398204 ap 0 c Black

131 14 1.0 ap 0 c Lavender l TF-DNA

208 72 0.27559550410241757 ap 0 c Black

184 71 0.30065973649117494 ap 0 c Black

45 36 0.6814339244948303 ap 0 c Black

184 72 0.16268153046528963 ap 0 c Black

134 296 0.39607207053865506 ap 0 c Black

184 73 0.1373998981440491 ap 0 c Black

229 71 0.38271164243875133 ap 0 c Black

104 314 0.15549709698770142 ap 0 c Black

274 186 0.516999887872986 ap 0 c Black

268 215 0.448855400840225 ap 0 c Black

178 291 0.4358933080166861 ap 0 c Black

105 116 0.5598303719457832 ap 0 c Black

212 161 0.34411568606441584 ap 0 c Black

188 186 1.0 ap 0 c Black

113 39 0.4761559226895662 ap 0 c Black

191 195 0.3806836101608239 ap 0 c Black

118 136 0.40301000097215234 ap 0 c Black

232 12 0.6189598143252396 ap 0 c Black

73 184 0.1373998981440491 ap 0 c Black

85 86 0.34683551129124607 ap 0 c Black

149 132 0.3787178710856305 ap 0 c Black

168 249 0.6715517124866173 ap 0 c Black

105 184 0.29784020617296303 ap 0 c Black

169 55 1.0 ap 0 c Black

115 30 1.0 ap 0 c Purple l Both

14 157 0.37182475528785647 ap 0 c Black

26 139 1.0 ap 0 c Blue l TF-DNA

267 259 0.546342266641591 ap 0 c Black

43 246 0.28189894915942904 ap 0 c Black

121 159 0.4094307169996534 ap 0 c Black

125 20 0.5433959406252865 ap 0 c Black

273 297 0.479611348047747 ap 0 c Black

131 188 1.0 ap 0 c Blue l TF-DNA

104 283 0.18080982203891435 ap 0 c Black

131 187 1.0 ap 0 c Lavender l TF-DNA

204 124 0.29415347253195406 ap 0 c Black

186 187 0.6861485640624241 ap 0 c Black

185 159 0.5268663019349323 ap 0 c Black

186 188 0.343699934713979 ap 0 c Black

162 186 1.0 ap 0 c Black

229 221 0.47903427010411037 ap 0 c Black

115 192 1.0 ap 0 c Lavender l TF-DNA

191 185 0.5681167435304667 ap 0 c Black

121 182 0.47620521764550267 ap 0 c Black

157 135 0.49220217181190745 ap 0 c Black

223 105 0.31097551473476215 ap 0 c Black

257 280 0.31755514945317725 ap 0 c Black

68 156 0.49401156160293735 ap 0 c Black

191 186 1.0 ap 0 c Black

39 46 1.0 ap 0 c Lavender l TF-DNA

128 23 0.4206587271597029 ap 0 c Black

290 121 0.3705015172145872 ap 0 c Black

5 2 0.20062341419201196 ap 0 c Black

115 167 1.0 ap 0 c Lavender l TF-DNA

260 72 0.4393376001162598 ap 0 c Black

191 184 0.2438557546958054 ap 0 c Black

233 272 0.5847838344586832 ap 0 c Black

141 29 0.1607263825425487 ap 0 c Black

116 105 0.5598303719457832 ap 0 c Black

296 100 0.4668259440927525 ap 0 c Black

274 184 0.21239169222143076 ap 0 c Black

25 198 1.0 ap 0 c Black

243 72 0.5352024943880083 ap 0 c Black

90 89 0.27695386181192194 ap 0 c Black

131 4 0.6336923912577093 ap 0 c Black

184 191 0.2438557546958054 ap 0 c Black

87 88 0.3839930299247852 ap 0 c Black

15 132 0.44854600338141004 ap 0 c Black

161 171 0.369320078614488 ap 0 c Black

84 70 0.2628852640574428 ap 0 c Black

291 178 0.4358933080166861 ap 0 c Black

162 185 0.4297287999608643 ap 0 c Black

30 285 0.49923100120929886 ap 0 c Black

64 215 0.4334542353546794 ap 0 c Black

115 158 1.0 ap 0 c Lavender l TF-DNA

131 258 0.3881033777860398 ap 0 c Black

204 127 0.6292103477127005 ap 0 c Black

131 3 1.0 ap 0 c Lavender l TF-DNA

72 132 0.4214094620914772 ap 0 c Black

150 157 0.44037896389468467 ap 0 c Black

73 162 0.23263288159888162 ap 0 c Black

72 131 0.4614257216780781 ap 0 c Black

131 39 0.2740943944363821 ap 0 c Black

115 166 1.0 ap 0 c Lavender l TF-DNA

115 23 1.0 ap 0 c Lavender l TF-DNA

44 234 1.0 ap 0 c Blue l TF-DNA

216 184 0.3137423808299148 ap 0 c Black

26 228 1.0 ap 0 c Lavender l TF-DNA

162 184 1.0 ap 0 c Black

170 180 1.0 ap 0 c Black

14 60 0.2601771393102726 ap 0 c Black

188 187 0.3253067802108278 ap 0 c Black

121 82 0.4073088831933932 ap 0 c Black

210 287 0.44578872568166034 ap 0 c Black

165 105 0.5890641167641978 ap 0 c Black

115 46 1.0 ap 0 c Lavender l TF-DNA

249 168 0.6715517124866173 ap 0 c Black

70 84 0.2628852640574428 ap 0 c Black

115 191 0.6565326110186523 ap 0 c Black

154 100 1.0 ap 0 c Black

94 75 0.5717189335837831 ap 0 c Black

26 101 1.0 ap 0 c Lavender l TF-DNA

175 200 0.4250569932843769 ap 0 c Black

218 226 0.6304720667430611 ap 0 c Black

231 66 0.23330533727670041 ap 0 c Black

112 35 0.19122411567153658 ap 0 c Black

277 131 0.2792052853300702 ap 0 c Black

9 139 1.0 ap 0 c Lavender l TF-DNA

222 194 0.5091604475773586 ap 0 c Black

105 165 0.5890641167641978 ap 0 c Black

176 248 0.2899190856638109 ap 0 c Black

301 104 0.18510061001084696 ap 0 c Black

187 186 1.0 ap 0 c Black

104 299 0.18229181227816466 ap 0 c Black

135 157 0.49220217181190745 ap 0 c Black

164 181 0.34018777850178694 ap 0 c Black

302 152 0.6314700569183479 ap 0 c Black

26 234 1.0 ap 0 c Lavender l TF-DNA

153 103 0.5906625476736882 ap 0 c Black

100 154 0.5213144257372303 ap 0 c Black

106 72 0.4870384114618382 ap 0 c Black

229 252 0.3785999063598686 ap 0 c Black

43 207 0.30951132781302043 ap 0 c Black

157 61 0.49623370779340825 ap 0 c Black

194 157 0.47291340731540615 ap 0 c Black

155 157 0.49992648514987825 ap 0 c Black

136 177 0.39558044372092216 ap 0 c Black

197 178 0.5540965112470485 ap 0 c Black

157 60 0.2935093096551368 ap 0 c Black

294 288 0.18141426272176422 ap 0 c Black

131 285 0.6817704926601744 ap 0 c Black

187 188 0.3253067802108278 ap 0 c Black

196 147 0.35706425105806827 ap 0 c Black

60 61 0.31840230361170907 ap 0 c Black

100 128 0.6093167987368007 ap 0 c Black

73 116 0.3732845099456223 ap 0 c Black

154 242 1.0 ap 0 c Black

227 191 0.5802990291435908 ap 0 c Black

306 131 0.4982185605440574 ap 0 c Black

32 204 0.2801619316889266 ap 0 c Black

154 128 0.6229374248738639 ap 0 c Black

115 116 1.0 ap 0 c Purple l Both

73 115 0.38912356889206706 ap 0 c Black

115 237 0.5668204543471446 ap 0 c Black

19 30 0.3689601226484943 ap 0 c Black

197 131 0.631016555923188 ap 0 c Black

66 265 0.3723066536422219 ap 0 c Black

116 72 0.546282520573828 ap 0 c Black

116 73 0.3732845099456223 ap 0 c Black

127 66 0.395484422967996 ap 0 c Black

115 118 1.0 ap 0 c Lavender l TF-DNA

188 184 0.3263339035702408 ap 0 c Black

267 226 0.5629883374535731 ap 0 c Black

66 238 0.2875489505036219 ap 0 c Black

105 227 0.618369999027194 ap 0 c Black

111 171 0.2889576076729204 ap 0 c Black

121 52 0.44084114598668533 ap 0 c Black

115 119 1.0 ap 0 c Lavender l TF-DNA

272 281 0.49318824641176284 ap 0 c Black

137 99 0.3989896576490773 ap 0 c Black

9 22 1.0 ap 0 c Lavender l TF-DNA

140 28 0.37067877393285364 ap 0 c Black

140 29 0.19555548275750978 ap 0 c Black

203 162 0.4078040146034043 ap 0 c Black

253 220 0.3535069892065875 ap 0 c Black

72 204 0.46121012546059253 ap 0 c Black

121 290 0.3705015172145872 ap 0 c Black

262 69 0.6526968860041908 ap 0 c Black

196 305 0.41376557453072765 ap 0 c Black

37 52 0.49085808144580906 ap 0 c Black

171 161 0.369320078614488 ap 0 c Black

281 229 0.39930239621876523 ap 0 c Black

23 255 0.5836884423479032 ap 0 c Black

60 14 0.2601771393102726 ap 0 c Black

61 157 0.49623370779340825 ap 0 c Black

107 103 0.629935707997234 ap 0 c Black

92 204 0.46339730667334145 ap 0 c Black

131 80 1.0 ap 0 c Lavender l TF-DNA

128 154 1.0 ap 0 c Black

17 200 0.576719803059603 ap 0 c Black

121 111 0.36280172536363314 ap 0 c Black

72 148 0.15910730538773551 ap 0 c Black

73 164 0.3933367865629602 ap 0 c Black

159 185 0.5268663019349323 ap 0 c Black

116 204 0.5546006240038455 ap 0 c Black

191 187 0.3538054910003756 ap 0 c Black

267 217 0.22840272937792927 ap 0 c Black

270 73 0.5091824297403772 ap 0 c Black

131 30 0.46259988592184764 ap 0 c Black

105 303 0.6346846155762462 ap 0 c Black

178 280 0.3771216069512711 ap 0 c Black

100 296 0.4668259440927525 ap 0 c Black

71 66 0.3744341283851667 ap 0 c Black

185 191 0.5681167435304667 ap 0 c Black

9 131 0.29940570499703645 ap 0 c Black

185 7 0.6496917621709739 ap 0 c Black

115 129 1.0 ap 0 c Lavender l TF-DNA

61 60 0.31840230361170907 ap 0 c Black

164 19 0.41697729856189714 ap 0 c Black

26 95 1.0 ap 0 c Blue l TF-DNA

131 166 1.0 ap 0 c Lavender l TF-DNA

182 242 1.0 ap 0 c Black

153 36 0.6697679278342372 ap 0 c Black

215 64 0.4334542353546794 ap 0 c Black

48 46 0.13215919578019492 ap 0 c Black

209 105 0.2733102511891893 ap 0 c Black

26 62 1.0 ap 0 c Blue l TF-DNA

157 131 0.4533532462849016 ap 0 c Black

131 107 0.6873535923377707 ap 0 c Black

128 90 1.0 ap 0 c Black

41 22 1.0 ap 0 c Lavender l TF-DNA

43 204 0.4653552326274724 ap 0 c Black

72 240 0.4721825538656083 ap 0 c Black

115 80 1.0 ap 0 c Lavender l TF-DNA

115 285 0.5559333486376676 ap 0 c Black

202 273 0.5321869874170707 ap 0 c Black

162 128 0.31084191742973977 ap 0 c Black

154 23 0.41625641335194513 ap 0 c Black

278 270 0.39936678092058614 ap 0 c Black

39 160 1.0 ap 0 c Lavender l TF-DNA

204 92 0.46339730667334145 ap 0 c Black

39 131 1.0 ap 0 c Purple l Both

131 81 1.0 ap 0 c Lavender l TF-DNA

105 225 0.1895823737396261 ap 0 c Black

115 187 1.0 ap 0 c Lavender l TF-DNA

26 31 1.0 ap 0 c Blue l TF-DNA

131 84 1.0 ap 0 c Lavender l TF-DNA

115 188 1.0 ap 0 c Blue l TF-DNA

204 231 0.298717902564725 ap 0 c Black

240 71 0.4789845357189038 ap 0 c Black

248 176 0.2899190856638109 ap 0 c Black

82 121 0.4073088831933932 ap 0 c Black

240 72 0.4721825538656083 ap 0 c Black

159 213 0.50534487568577 ap 0 c Black

179 105 0.28520324586008605 ap 0 c Black

244 224 0.4566093544779133 ap 0 c Black

128 100 1.0 ap 0 c Black

44 98 1.0 ap 0 c Lavender l TF-DNA

54 53 0.4203982212281799 ap 0 c Black

54 24 0.40134400538521897 ap 0 c Black

128 255 0.5974790231875076 ap 0 c Black

66 151 0.253243101258263 ap 0 c Black

157 14 0.37182475528785647 ap 0 c Black

107 131 0.6873535923377707 ap 0 c Black

4 157 0.39855343718341907 ap 0 c Black

186 193 0.5541348810092542 ap 0 c Black

128 87 1.0 ap 0 c Black

128 88 1.0 ap 0 c Black

94 166 1.0 ap 0 c Lavender l TF-DNA

259 217 0.29117003906834915 ap 0 c Black

105 171 0.44484678582193055 ap 0 c Black

50 51 0.5907166026682449 ap 0 c Black

272 233 0.5847838344586832 ap 0 c Black

157 130 0.4419027011115432 ap 0 c Black

197 115 0.507426833856509 ap 0 c Black

280 269 0.21766653541810854 ap 0 c Black

292 268 0.2947094523461154 ap 0 c Black

285 131 0.6817704926601744 ap 0 c Black

164 191 0.4508464427741266 ap 0 c Black

154 136 0.37393862921607346 ap 0 c Black

7 164 0.5219330324636395 ap 0 c Black

148 72 0.15910730538773551 ap 0 c Black

115 18 1.0 ap 0 c Lavender l TF-DNA

194 222 0.5091604475773586 ap 0 c Black

50 49 0.5651705986676755 ap 0 c Black

59 106 0.44705573624029277 ap 0 c Black

169 180 1.0 ap 0 c Black

154 121 1.0 ap 0 c Black

280 257 0.31755514945317725 ap 0 c Black

293 284 0.39450057772394886 ap 0 c Black

20 125 0.5433959406252865 ap 0 c Black

183 264 0.26064483504997615 ap 0 c Black

128 83 1.0 ap 0 c Black

109 313 0.36332829735430566 ap 0 c Black

217 218 0.29277450443837094 ap 0 c Black

35 112 0.19122411567153658 ap 0 c Black

46 48 1.0 ap 0 c Black

185 312 0.4593735443483068 ap 0 c Black

39 138 1.0 ap 0 c Lavender l TF-DNA

200 17 0.576719803059603 ap 0 c Black

251 270 0.35195700847714473 ap 0 c Black

213 159 0.50534487568577 ap 0 c Black

156 67 0.48013602704804886 ap 0 c Black

304 154 0.5389349266250408 ap 0 c Black

44 39 1.0 ap 0 c Lavender l TF-DNA

89 90 1.0 ap 0 c Black

115 19 1.0 ap 0 c Lavender l TF-DNA

131 74 1.0 ap 0 c Lavender l TF-DNA

66 65 0.5277689717630464 ap 0 c Black

200 175 0.4250569932843769 ap 0 c Black

54 38 0.4160604256945295 ap 0 c Black

132 15 0.44854600338141004 ap 0 c Black

184 164 0.2244500878594139 ap 0 c Black

39 92 1.0 ap 0 c Black l TF-DNA

164 71 0.3708991815483336 ap 0 c Black

310 88 1.0 ap 0 c Black

312 185 0.4593735443483068 ap 0 c Black

19 164 0.41697729856189714 ap 0 c Black

226 267 0.5629883374535731 ap 0 c Black

71 240 0.4789845357189038 ap 0 c Black

156 68 0.49401156160293735 ap 0 c Black

164 73 0.3933367865629602 ap 0 c Black

115 164 1.0 ap 0 c Lavender l TF-DNA

164 72 0.4136695836456755 ap 0 c Black

105 179 0.28520324586008605 ap 0 c Black

115 93 1.0 ap 0 c Lavender l TF-DNA

187 184 0.330459387196758 ap 0 c Black

189 82 0.26793645517040154 ap 0 c Black

115 22 1.0 ap 0 c Lavender l TF-DNA

214 185 0.5858773189953106 ap 0 c Black

72 197 0.46399560301119946 ap 0 c Black

131 139 1.0 ap 0 c Lavender l TF-DNA

131 138 1.0 ap 0 c Lavender l TF-DNA

260 6 0.6250516309141246 ap 0 c Black

159 121 0.4094307169996534 ap 0 c Black

104 300 0.1591533900343301 ap 0 c Black

287 210 0.44578872568166034 ap 0 c Black

127 185 0.6580840863874308 ap 0 c Black

264 183 0.26064483504997615 ap 0 c Black

103 153 0.5906625476736882 ap 0 c Black

115 94 1.0 ap 0 c Purple l Both

131 7 1.0 ap 0 c Lavender l TF-DNA

131 143 1.0 ap 0 c Lavender l TF-DNA

204 238 0.31666556613214036 ap 0 c Black

157 155 0.49992648514987825 ap 0 c Black

162 164 0.3074564285921982 ap 0 c Black

7 162 0.47118989833956715 ap 0 c Black

82 307 0.3896488787389444 ap 0 c Black

268 292 0.2947094523461154 ap 0 c Black

115 156 1.0 ap 0 c Lavender l TF-DNA

177 136 0.39558044372092216 ap 0 c Black

131 306 0.4982185605440574 ap 0 c Black

215 268 0.448855400840225 ap 0 c Black

116 131 1.0 ap 0 c Black

131 8 1.0 ap 0 c Lavender l TF-DNA

116 132 0.5362195273457306 ap 0 c Black

204 239 0.46229888231925276 ap 0 c Black

128 182 0.3644278115835904 ap 0 c Black

25 171 0.466488674763306 ap 0 c Black

154 304 0.5389349266250408 ap 0 c Black

284 293 0.39450057772394886 ap 0 c Black

259 267 0.546342266641591 ap 0 c Black

42 61 0.5014121405147607 ap 0 c Black

7 160 0.4025096690924074 ap 0 c Black

104 301 0.18510061001084696 ap 0 c Black

49 50 0.5651705986676755 ap 0 c Black

49 51 0.6803096508671154 ap 0 c Black

160 308 0.2487352328100839 ap 0 c Black

71 229 0.38271164243875133 ap 0 c Black

68 65 0.49994178291597663 ap 0 c Black

212 115 0.5176701623436856 ap 0 c Black

171 105 0.44484678582193055 ap 0 c Black

171 106 0.4573053098352048 ap 0 c Black

131 111 0.46416217419465505 ap 0 c Black

185 72 0.44779806101477204 ap 0 c Black

224 244 0.4566093544779133 ap 0 c Black

185 73 0.40040032189249264 ap 0 c Black

115 9 1.0 ap 0 c Purple l Both

26 29 1.0 ap 0 c Lavender l TF-DNA

39 113 0.4761559226895662 ap 0 c Black

237 131 0.4886342166303141 ap 0 c Black

19 121 0.40372126554925614 ap 0 c Black

170 117 0.20315517968933403 ap 0 c Black

26 35 1.0 ap 0 c Blue l TF-DNA

37 128 0.5412179384958775 ap 0 c Black

298 24 0.3787424858592195 ap 0 c Black

186 184 0.323610760976149 ap 0 c Black

157 150 0.44037896389468467 ap 0 c Black

204 69 0.6402178536530986 ap 0 c Black

115 184 1.0 ap 0 c Lavender l TF-DNA

30 115 0.5215691845184668 ap 0 c Black

115 35 1.0 ap 0 c Lavender l TF-DNA

39 22 1.0 ap 0 c Lavender l TF-DNA

115 31 1.0 ap 0 c Lavender l TF-DNA

24 298 0.3787424858592195 ap 0 c Black

162 197 0.4002797877374178 ap 0 c Black

270 278 0.39936678092058614 ap 0 c Black

39 20 1.0 ap 0 c Lavender l TF-DNA

307 82 0.3896488787389444 ap 0 c Black

127 204 0.6292103477127005 ap 0 c Black

184 162 0.2877824923962609 ap 0 c Black

133 132 0.41237134880644727 ap 0 c Black

164 162 0.3074564285921982 ap 0 c Black

132 131 0.43494197092599485 ap 0 c Black

71 131 0.4814633165238511 ap 0 c Black

121 116 0.4345699894750008 ap 0 c Black

132 133 0.41237134880644727 ap 0 c Black

145 139 0.23337999233279924 ap 0 c Black

7 19 0.42115976186096443 ap 0 c Black

117 170 0.20315517968933403 ap 0 c Black

102 115 0.40649221556106097 ap 0 c Black

142 28 1.0 ap 0 c Black

229 233 0.39832172988096476 ap 0 c Black

241 137 0.2962099061300464 ap 0 c Black

142 29 0.14547631803644356 ap 0 c Black

184 161 0.20386964523350545 ap 0 c Black

104 156 0.1561691629509758 ap 0 c Black

280 178 0.3771216069512711 ap 0 c Black

184 160 0.15015862518025225 ap 0 c Black

258 131 0.3881033777860398 ap 0 c Black

299 104 0.18229181227816466 ap 0 c Black

72 260 0.4393376001162598 ap 0 c Black

233 250 0.6016102937555475 ap 0 c Black

272 157 0.466297913262682 ap 0 c Black

255 100 0.45482486486005386 ap 0 c Black

131 144 1.0 ap 0 c Lavender l TF-DNA

6 72 0.4570244738241297 ap 0 c Black

191 151 0.4131767740128001 ap 0 c Black

164 161 0.43353014708444765 ap 0 c Black

131 132 1.0 ap 0 c Purple l Both

39 230 1.0 ap 0 c Blue l TF-DNA

181 164 0.34018777850178694 ap 0 c Black

162 160 0.3920217594792542 ap 0 c Black

228 258 0.403592034480032 ap 0 c Black

184 187 0.330459387196758 ap 0 c Black

258 228 0.403592034480032 ap 0 c Black

105 209 0.2733102511891893 ap 0 c Black

157 194 0.47291340731540615 ap 0 c Black

184 188 0.3263339035702408 ap 0 c Black

55 117 0.14851934051546847 ap 0 c Black

204 227 0.6358532216505587 ap 0 c Black

131 234 1.0 ap 0 c Blue l TF-DNA

182 52 0.38825167904390245 ap 0 c Black

47 48 1.0 ap 0 c Black

53 54 0.4203982212281799 ap 0 c Black

204 43 0.4653552326274724 ap 0 c Black

72 30 0.42640401746212525 ap 0 c Black

115 185 1.0 ap 0 c Lavender l TF-DNA

66 231 0.23330533727670041 ap 0 c Black

106 105 0.4681738462002579 ap 0 c Black

30 19 0.3689601226484943 ap 0 c Black

283 104 0.18080982203891435 ap 0 c Black

255 23 0.5836884423479032 ap 0 c Black

170 55 1.0 ap 0 c Black

91 97 0.23239944683244448 ap 0 c Black

184 186 0.323610760976149 ap 0 c Black

115 132 1.0 ap 0 c Purple l Both

115 131 0.42820479349937685 ap 0 c Black

270 251 0.35195700847714473 ap 0 c Black

307 128 0.2920439650638414 ap 0 c Black

86 85 0.34683551129124607 ap 0 c Black

30 131 0.46259988592184764 ap 0 c Black

137 241 0.2962099061300464 ap 0 c Black

160 7 0.4025096690924074 ap 0 c Black

226 218 0.6304720667430611 ap 0 c Black

60 131 0.3291584196738823 ap 0 c Black

162 71 0.4359985311201446 ap 0 c Black

71 115 0.5641625514920002 ap 0 c Black

162 72 0.33647522154480075 ap 0 c Black

297 109 0.35204170646774474 ap 0 c Black

162 73 0.23263288159888162 ap 0 c Black

131 112 1.0 ap 0 c Lavender l TF-DNA

4 131 0.6336923912577093 ap 0 c Black

73 185 0.40040032189249264 ap 0 c Black

121 19 0.40372126554925614 ap 0 c Black

255 154 0.6087465738954273 ap 0 c Black

106 171 0.4573053098352048 ap 0 c Black

225 105 0.1895823737396261 ap 0 c Black

275 57 0.381908126053177 ap 0 c Black

56 63 0.30042504019302063 ap 0 c Black

238 204 0.31666556613214036 ap 0 c Black

71 184 0.30065973649117494 ap 0 c Black

51 50 0.5907166026682449 ap 0 c Black

171 111 0.2889576076729204 ap 0 c Black

160 162 1.0 ap 0 c Black

24 171 0.49625114048090513 ap 0 c Black

105 126 1.0 ap 0 c Black

86 235 0.23488613831910496 ap 0 c Black

131 163 0.5652157743603462 ap 0 c Black

66 32 0.22779940263200232 ap 0 c Black

185 214 0.5858773189953106 ap 0 c Black

204 72 0.46121012546059253 ap 0 c Black

227 105 0.618369999027194 ap 0 c Black

19 124 0.25079227562239914 ap 0 c Black

218 217 0.29277450443837094 ap 0 c Black

272 261 0.3979153316136415 ap 0 c Black

51 49 0.6803096508671154 ap 0 c Black

67 156 0.48013602704804886 ap 0 c Black

29 142 0.14547631803644356 ap 0 c Black

26 34 0.15030827424537813 ap 0 c Black

139 28 1.0 ap 0 c Black

161 212 0.34411568606441584 ap 0 c Black

204 32 0.2801619316889266 ap 0 c Black

172 174 0.47372835680009456 ap 0 c Black

69 262 0.6526968860041908 ap 0 c Black

162 203 0.4078040146034043 ap 0 c Black

82 81 0.27730029738843576 ap 0 c Black

29 141 0.1607263825425487 ap 0 c Black

255 128 0.5974790231875076 ap 0 c Black

180 170 0.2936584950916059 ap 0 c Black

180 169 0.28387394904460655 ap 0 c Black

69 206 0.6872857074204007 ap 0 c Black

29 140 0.19555548275750978 ap 0 c Black

107 115 0.5099566332425173 ap 0 c Black

154 255 1.0 ap 0 c Black

178 197 0.5540965112470485 ap 0 c Black

115 122 1.0 ap 0 c Lavender l TF-DNA

226 217 0.29337336194627706 ap 0 c Black

131 22 1.0 ap 0 c Lavender l TF-DNA

219 131 0.5842258632287534 ap 0 c Black

162 7 0.47118989833956715 ap 0 c Black

72 6 0.4570244738241297 ap 0 c Black

151 66 0.253243101258263 ap 0 c Black

139 145 0.23337999233279924 ap 0 c Black

203 72 0.44109648506285337 ap 0 c Black

191 115 0.6565326110186523 ap 0 c Black

128 121 0.3616975618647896 ap 0 c Black

131 277 0.2792052853300702 ap 0 c Black

309 73 0.5441838786524129 ap 0 c Black

193 186 0.5541348810092542 ap 0 c Black

184 105 0.29784020617296303 ap 0 c Black

131 219 0.5842258632287534 ap 0 c Black

131 164 1.0 ap 0 c Lavender l TF-DNA

39 27 1.0 ap 0 c Lavender l TF-DNA

72 162 0.33647522154480075 ap 0 c Black

71 21 0.49823358034928855 ap 0 c Black

115 102 0.40649221556106097 ap 0 c Black

268 286 0.34254843187719686 ap 0 c Black

121 154 0.3735313171683927 ap 0 c Black

191 164 0.4508464427741266 ap 0 c Black

26 25 1.0 ap 0 c Lavender l TF-DNA

250 233 0.6016102937555475 ap 0 c Black

202 60 0.23769732745013633 ap 0 c Black

104 262 0.20733168448739422 ap 0 c Black

83 74 0.48156683390526334 ap 0 c Black

227 276 0.19526896333938196 ap 0 c Black

44 89 1.0 ap 0 c Lavender l TF-DNA

157 4 0.39855343718341907 ap 0 c Black

115 67 1.0 ap 0 c Lavender l TF-DNA

276 227 0.19526896333938196 ap 0 c Black

26 74 1.0 ap 0 c Blue l TF-DNA

88 310 0.3528412404342649 ap 0 c Black

201 245 0.3541001129648362 ap 0 c Black

88 311 0.36678266998808 ap 0 c Black

77 79 0.357298650755274 ap 0 c Black

94 115 0.6358310041016854 ap 0 c Black

131 46 1.0 ap 0 c Lavender l TF-DNA

32 66 0.22779940263200232 ap 0 c Black

181 195 0.30334726516551574 ap 0 c Black

136 154 0.37393862921607346 ap 0 c Black

205 83 1.0 ap 0 c Black

115 160 1.0 ap 0 c Lavender l TF-DNA

263 287 0.61239161461075 ap 0 c Black

217 267 0.22840272937792927 ap 0 c Black

286 268 0.34254843187719686 ap 0 c Black

111 131 0.46416217419465505 ap 0 c Black

96 9 0.29033010754979977 ap 0 c Black

211 271 0.295866509811291 ap 0 c Black

44 74 1.0 ap 0 c Lavender l TF-DNA

266 194 0.616844047802272 ap 0 c Black

185 164 0.5551252265409474 ap 0 c Black

34 26 0.15030827424537813 ap 0 c Black

131 192 1.0 ap 0 c Lavender l TF-DNA

24 54 0.40134400538521897 ap 0 c Black

296 134 0.39607207053865506 ap 0 c Black

281 272 0.49318824641176284 ap 0 c Black

273 202 0.5321869874170707 ap 0 c Black

16 13 0.4703063612892095 ap 0 c Black

131 60 0.3291584196738823 ap 0 c Black

160 184 0.15015862518025225 ap 0 c Black

115 107 0.5099566332425173 ap 0 c Black

164 184 0.2244500878594139 ap 0 c Black

82 189 0.26793645517040154 ap 0 c Black

121 304 0.4025728077987404 ap 0 c Black

13 123 0.45183382828324836 ap 0 c Black

197 105 0.5835954660184537 ap 0 c Black

131 197 0.631016555923188 ap 0 c Black

115 277 0.19980341355612483 ap 0 c Black

245 201 0.3541001129648362 ap 0 c Black

136 10 1.0 ap 0 c Black

300 104 0.1591533900343301 ap 0 c Black

171 25 0.466488674763306 ap 0 c Black

254 116 0.409131309377238 ap 0 c Black

131 89 1.0 ap 0 c Lavender l TF-DNA

226 259 0.6007292980494991 ap 0 c Black

6 260 0.6250516309141246 ap 0 c Black

38 54 0.4160604256945295 ap 0 c Black

185 162 0.4297287999608643 ap 0 c Black

128 162 0.31084191742973977 ap 0 c Black

88 87 0.3839930299247852 ap 0 c Black

190 236 0.42848324609013 ap 0 c Black

39 81 1.0 ap 0 c Lavender l TF-DNA

116 254 0.409131309377238 ap 0 c Black

105 223 0.31097551473476215 ap 0 c Black

9 115 0.4374175514297528 ap 0 c Black

235 86 0.23488613831910496 ap 0 c Black

116 115 1.0 ap 0 c Black

197 162 0.4002797877374178 ap 0 c Black

314 104 0.15549709698770142 ap 0 c Black

295 104 0.1769730085325099 ap 0 c Black

10 9 1.0 ap 0 c Purple l Both

132 73 0.38930547543812716 ap 0 c Black

39 84 1.0 ap 0 c Lavender l TF-DNA

63 56 0.30042504019302063 ap 0 c Black

105 71 0.6045054390859078 ap 0 c Black

161 184 0.20386964523350545 ap 0 c Black

157 272 0.466297913262682 ap 0 c Black

164 185 0.5551252265409474 ap 0 c Black

105 72 0.516188891733907 ap 0 c Black

105 73 0.4393933987810719 ap 0 c Black

163 131 0.5652157743603462 ap 0 c Black

19 185 0.42818824250392745 ap 0 c Black

103 76 1.0 ap 0 c Lavender l TF-DNA

26 108 1.0 ap 0 c Blue l TF-DNA

194 266 0.616844047802272 ap 0 c Black

132 72 0.4214094620914772 ap 0 c Black

115 74 1.0 ap 0 c Lavender l TF-DNA

73 131 0.3987127174935446 ap 0 c Black

73 132 0.38930547543812716 ap 0 c Black

26 47 1.0 ap 0 c Blue l TF-DNA

79 77 0.357298650755274 ap 0 c Black

131 71 0.4814633165238511 ap 0 c Black

131 72 0.4614257216780781 ap 0 c Black

161 285 0.5310431068084502 ap 0 c Black

79 78 0.29919714886718923 ap 0 c Black

26 46 1.0 ap 0 c Blue l TF-DNA

131 73 0.3987127174935446 ap 0 c Black

66 71 0.3744341283851667 ap 0 c Black

182 121 1.0 ap 0 c Black

72 203 0.44109648506285337 ap 0 c Black

128 37 0.5412179384958775 ap 0 c Black

72 105 0.516188891733907 ap 0 c Black

72 106 0.4870384114618382 ap 0 c Black

## The jActiveModule network

*Vertices 270

1 "273 (AMPH)" ellipses_6 ic Blue fos 15

2 "330 (BIRC3)" ellipses_6 ic Red fos 15

3 "466 (ATF1)" diamonds_10 ic Cyan bw 10 fos 15

4 "467 (ATF3)" diamonds_10 ic Red bw 10 fos 15

5 "476 (ATP1A1)" triangles_10 ic LightYellow bw 10 fos 15

6 "545 (ATR)" triangles_10 ic NavyBlue bw 10 fos 15

7 "580 (BARD1)" ellipses_6 ic Cyan fos 15

8 "597 (BCL2A1)" ellipses_6 ic Red fos 15

9 "605 (BCL7A)" ellipses_6 ic Blue fos 15

10 "637 (BID)" ellipses_6 ic Red fos 15

11 "639 (PRDM1)" ellipses_6 ic Red fos 15

12 "666 (BOK)" ellipses_6 ic White fos 15

13 "675 (BRCA2)" diamonds_10 ic White bw 10 fos 15

14 "694 (BTG1)" ellipses_6 ic Red fos 15

15 "819 (CAMLG)" ellipses_6 ic Blue fos 15

16 "835 (CASP2)" ellipses_6 ic Cyan fos 15

17 "843 (CASP10)" ellipses_6 ic LightYellow fos 15

18 "867 (CBL)" diamonds_10 ic Cyan bw 10 fos 15

19 "898 (CCNE1)" diamonds_10 ic Cyan bw 10 fos 15

20 "902 (CCNH)" ellipses_6 ic White fos 15

21 "969 (CD69)" triangles_10 ic Red bw 10 fos 15

22 "983 (CDC2)" diamonds_10 ic White bw 10 fos 15

23 "994 (CDC25B)" ellipses_6 ic Blue fos 15

24 "1024 (CDK8)" ellipses_6 ic Cyan fos 15

25 "1027 (CDKN1B)" diamonds_10 ic Blue bw 10 fos 15

26 "1029 (CDKN2A)" triangles_10 ic White bw 10 fos 15

27 "1050 (CEBPA)" diamonds_10 ic Blue bw 10 fos 15

28 "1052 (CEBPD)" diamonds_10 ic White bw 10 fos 15

29 "1060 (CENPC1)" ellipses_6 ic Blue fos 15

30 "1230 (CCR1)" triangles_10 ic Blue bw 10 fos 15

31 "1326 (MAP3K8)" ellipses_6 ic Red fos 15

32 "1387 (CREBBP)" boxs_10 ic White bw 10 fos 15

33 "1503 (CTPS)" ellipses_6 ic Blue fos 15

34 "1616 (DAXX)" ellipses_6 ic Yellow fos 15

35 "1642 (DDB1)" ellipses_6 ic White fos 15

36 "1647 (GADD45A)" diamonds_10 ic Red bw 10 fos 15

37 "1649 (DDIT3)" diamonds_10 ic Cyan bw 10 fos 15

38 "1677 (DFFB)" ellipses_6 ic Blue fos 15

39 "1786 (DNMT1)" ellipses_6 ic NavyBlue fos 15

40 "1796 (DOK1)" ellipses_6 ic Blue fos 15

41 "1847 (DUSP5)" ellipses_6 ic Red fos 15

42 "1857 (DVL3)" ellipses_6 ic LightCyan fos 15

43 "1871 (E2F3)" diamonds_10 ic White bw 10 fos 15

44 "1876 (E2F6)" diamonds_10 ic Cyan bw 10 fos 15

45 "1877 (E4F1)" ellipses_6 ic Blue fos 15

46 "1880 (EBI2)" ellipses_6 ic Red fos 15

47 "1942 (EFNA1)" triangles_10 ic Red bw 10 fos 15

48 "1945 (EFNA4)" triangles_10 ic Blue bw 10 fos 15

49 "1969 (EPHA2)" ellipses_6 ic Yellow fos 15

50 "2002 (ELK1)" diamonds_10 ic White bw 10 fos 15

51 "2026 (ENO2)" ellipses_6 ic Cyan fos 15

52 "2029 (ENSA)" ellipses_6 ic Yellow fos 15

53 "2068 (ERCC2)" ellipses_6 ic LightCyan fos 15

54 "2073 (ERCC5)" ellipses_6 ic Cyan fos 15

55 "2074 (ERCC6)" ellipses_6 ic LightCyan fos 15

56 "2099 (ESR1)" diamonds_10 ic White bw 10 fos 15

57 "2146 (EZH2)" ellipses_6 ic Red fos 15

58 "2152 (F3)" ellipses_6 ic Red fos 15

59 "2242 (FES)" ellipses_6 ic Blue fos 15

60 "2288 (FKBP4)" ellipses_6 ic Blue fos 15

61 "2309 (FOXO3)" diamonds_10 ic White bw 10 fos 15

62 "2526 (FUT4)" ellipses_6 ic Red fos 15

63 "2535 (FZD2)" triangles_10 ic Blue bw 10 fos 15

64 "2591 (GALNT3)" ellipses_6 ic Red fos 15

65 "2669 (GEM)" ellipses_6 ic Red fos 15

66 "2745 (GLRX)" ellipses_6 ic Red fos 15

67 "2935 (GSPT1)" ellipses_6 ic Cyan fos 15

68 "2959 (GTF2B)" diamonds_10 ic Red bw 10 fos 15

69 "3054 (HCFC1)" ellipses_6 ic LightCyan fos 15

70 "3065 (HDAC1)" ellipses_6 ic White fos 15

71 "3104 (ZBTB48)" ellipses_6 ic Blue fos 15

72 "3109 (HLA-DMB)" triangles_10 ic Red bw 10 fos 15

73 "3117 (HLA-DQA1)" triangles_10 ic LightYellow bw 10 fos 15

74 "3122 (HLA-DRA)" ellipses_6 ic Yellow fos 15

75 "3134 (HLA-F)" triangles_10 ic Red bw 10 fos 15

76 "3159 (HMGA1)" ellipses_6 ic White fos 15

77 "3183 (HNRNPC)" ellipses_6 ic Yellow fos 15

78 "3269 (HRH1)" triangles_10 ic Blue bw 10 fos 15

79 "3300 (DNAJB2)" ellipses_6 ic Blue fos 15

80 "3301 (DNAJA1)" ellipses_6 ic Red fos 15

81 "3305 (HSPA1L)" ellipses_6 ic Blue fos 15

82 "3383 (ICAM1)" triangles_10 ic Red bw 10 fos 15

83 "3399 (ID3)" ellipses_6 ic Blue fos 15

84 "3456 (IFNB1)" triangles_10 ic Red bw 10 fos 15

85 "3553 (IL1B)" triangles_10 ic Red bw 10 fos 15

86 "3556 (IL1RAP)" triangles_10 ic Red bw 10 fos 15

87 "3566 (IL4R)" triangles_10 ic White bw 10 fos 15

88 "3569 (IL6)" triangles_10 ic Red bw 10 fos 15

89 "3579 (IL8RB)" ellipses_6 ic Blue fos 15

90 "3587 (IL10RA)" ellipses_6 ic Red fos 15

91 "3593 (IL12B)" triangles_10 ic Red bw 10 fos 15

92 "3595 (IL12RB2)" ellipses_6 ic Red fos 15

93 "3627 (CXCL10)" ellipses_6 ic Red fos 15

94 "3659 (IRF1)" diamonds_10 ic Red bw 10 fos 15

95 "3662 (IRF4)" diamonds_10 ic White bw 10 fos 15

96 "3667 (IRS1)" diamonds_10 ic LightYellow bw 10 fos 15

97 "3716 (JAK1)" ellipses_6 ic Yellow fos 15

98 "3726 (JUNB)" diamonds_10 ic Red bw 10 fos 15

99 "3779 (KCNMB1)" triangles_10 ic Red bw 10 fos 15

100 "3838 (KPNA2)" ellipses_6 ic White fos 15

101 "3845 (KRAS)" triangles_10 ic White bw 10 fos 15

102 "4066 (LYL1)" ellipses_6 ic Blue fos 15

103 "4089 (SMAD4)" diamonds_10 ic White bw 10 fos 15

104 "4140 (MARK3)" ellipses_6 ic LightYellow fos 15

105 "4152 (MBD1)" ellipses_6 ic LightYellow fos 15

106 "4170 (MCL1)" ellipses_6 ic Orange fos 15

107 "4190 (MDH1)" ellipses_6 ic Blue fos 15

108 "4217 (MAP3K5)" ellipses_6 ic Blue fos 15

109 "4261 (CIITA)" ellipses_6 ic Cyan fos 15

110 "4356 (MPP3)" ellipses_6 ic Orange fos 15

111 "4435 (CITED1)" ellipses_6 ic Red fos 15

112 "4683 (NBN)" ellipses_6 ic Orange fos 15

113 "4691 (NCL)" ellipses_6 ic White fos 15

114 "4778 (NFE2)" diamonds_10 ic Blue bw 10 fos 15

115 "4790 (NFKB1)" diamonds_10 ic Red bw 10 fos 15

116 "4800 (NFYA)" diamonds_10 ic LightYellow bw 10 fos 15

117 "4801 (NFYB)" diamonds_10 ic LightCyan bw 10 fos 15

118 "4802 (NFYC)" diamonds_10 ic White bw 10 fos 15

119 "4814 (NINJ1)" ellipses_6 ic Red fos 15

120 "4899 (NRF1)" diamonds_10 ic Cyan bw 10 fos 15

121 "4913 (NTHL1)" ellipses_6 ic Blue fos 15

122 "5079 (PAX5)" diamonds_10 ic White bw 10 fos 15

123 "5366 (PMAIP1)" ellipses_6 ic Red fos 15

124 "5468 (PPARG)" diamonds_10 ic White bw 10 fos 15

125 "5499 (PPP1CA)" ellipses_6 ic White fos 15

126 "5515 (PPP2CA)" diamonds_10 ic Yellow bw 10 fos 15

127 "5599 (MAPK8)" diamonds_10 ic LightYellow bw 10 fos 15

128 "5606 (MAP2K3)" ellipses_6 ic Red fos 15

129 "5687 (PSMA6)" ellipses_6 ic Red fos 15

130 "5702 (PSMC3)" ellipses_6 ic Yellow fos 15

131 "5732 (PTGER2)" triangles_10 ic Red bw 10 fos 15

132 "5770 (PTPN1)" ellipses_6 ic Orange fos 15

133 "5806 (PTX3)" ellipses_6 ic Red fos 15

134 "5864 (RAB3A)" ellipses_6 ic Blue fos 15

135 "5893 (RAD52)" ellipses_6 ic White fos 15

136 "5931 (RBBP7)" ellipses_6 ic White fos 15

137 "5971 (RELB)" diamonds_10 ic Red bw 10 fos 15

138 "5977 (DPF2)" ellipses_6 ic Blue fos 15

139 "5987 (TRIM27)" ellipses_6 ic Blue fos 15

140 "6204 (RPS10)" ellipses_6 ic White fos 15

141 "6351 (CCL4)" ellipses_6 ic Red fos 15

142 "6352 (CCL5)" ellipses_6 ic Red fos 15

143 "6364 (CCL20)" ellipses_6 ic Red fos 15

144 "6389 (SDHA)" ellipses_6 ic Blue fos 15

145 "6426 (SFRS1)" ellipses_6 ic White fos 15

146 "6453 (ITSN1)" triangles_10 ic LightCyan bw 10 fos 15

147 "6456 (SH3GL2)" ellipses_6 ic LightYellow fos 15

148 "6457 (SH3GL3)" ellipses_6 ic White fos 15

149 "6624 (FSCN1)" ellipses_6 ic Red fos 15

150 "6790 (AURKA)" ellipses_6 ic Cyan fos 15

151 "6874 (TAF4)" ellipses_6 ic Cyan fos 15

152 "6877 (TAF5)" ellipses_6 ic Blue fos 15

153 "6878 (TAF6)" ellipses_6 ic Cyan fos 15

154 "6880 (TAF9)" ellipses_6 ic LightYellow fos 15

155 "6883 (TAF12)" ellipses_6 ic White fos 15

156 "6890 (TAP1)" ellipses_6 ic Red fos 15

157 "7077 (TIMP2)" ellipses_6 ic White fos 15

158 "7097 (TLR2)" triangles_10 ic Red bw 10 fos 15

159 "7124 (TNF)" triangles_10 ic Red bw 10 fos 15

160 "7128 (TNFAIP3)" ellipses_6 ic Red fos 15

161 "7132 (TNFRSF1A)" triangles_10 ic Blue bw 10 fos 15

162 "7150 (TOP1)" ellipses_6 ic LightYellow fos 15

163 "7157 (TP53)" diamonds_10 ic LightYellow bw 10 fos 15

164 "7159 (TP53BP2)" ellipses_6 ic Red fos 15

165 "7178 (TPT1)" ellipses_6 ic Yellow fos 15

166 "7185 (TRAF1)" ellipses_6 ic Red fos 15

167 "7251 (TSG101)" ellipses_6 ic White fos 15

168 "7324 (UBE2E1)" ellipses_6 ic Red fos 15

169 "7341 (SUMO1)" ellipses_6 ic White fos 15

170 "7494 (XBP1)" ellipses_6 ic Red fos 15

171 "7507 (XPA)" ellipses_6 ic NavyBlue fos 15

172 "7529 (YWHAB)" ellipses_6 ic LightCyan fos 15

173 "7832 (BTG2)" ellipses_6 ic Red fos 15

174 "8321 (FZD1)" triangles_10 ic Red bw 10 fos 15

175 "8445 (DYRK2)" ellipses_6 ic Blue fos 15

176 "8487 (SIP1)" ellipses_6 ic Cyan fos 15

177 "8638 (OASL)" ellipses_6 ic Red fos 15

178 "8645 (KCNK5)" triangles_10 ic Red bw 10 fos 15

179 "8676 (STX11)" ellipses_6 ic Red fos 15

180 "8737 (RIPK1)" ellipses_6 ic Red fos 15

181 "8767 (RIPK2)" ellipses_6 ic Red fos 15

182 "8772 (FADD)" ellipses_6 ic Blue fos 15

183 "8795 (TNFRSF10B)" triangles_10 ic Red bw 10 fos 15

184 "8809 (IL18R1)" ellipses_6 ic Red fos 15

185 "8837 (CFLAR)" ellipses_6 ic Red fos 15

186 "8870 (IER3)" ellipses_6 ic Red fos 15

187 "8996 (NOL3)" ellipses_6 ic Cyan fos 15

188 "9001 (HAP1)" ellipses_6 ic White fos 15

189 "9046 (DOK2)" ellipses_6 ic Blue fos 15

190 "9112 (MTA1)" ellipses_6 ic LightYellow fos 15

191 "9134 (CCNE2)" diamonds_10 ic Red bw 10 fos 15

192 "9232 (PTTG1)" ellipses_6 ic White fos 15

193 "9308 (CD83)" triangles_10 ic Red bw 10 fos 15

194 "9586 (CREB5)" ellipses_6 ic Blue fos 15

195 "9700 (ESPL1)" ellipses_6 ic Red fos 15

196 "9734 (HDAC9)" ellipses_6 ic Cyan fos 15

197 "9858 (KIAA0649)" ellipses_6 ic Blue fos 15

198 "9968 (MED12)" ellipses_6 ic LightYellow fos 15

199 "10023 (FRAT1)" ellipses_6 ic Blue fos 15

200 "10197 (PSME3)" ellipses_6 ic White fos 15

201 "10260 (DENND4A)" ellipses_6 ic Red fos 15

202 "10285 (SMNDC1)" ellipses_6 ic LightCyan fos 15

203 "10301 (DLEU1)" ellipses_6 ic White fos 15

204 "10425 (ARIH2)" ellipses_6 ic White fos 15

205 "10615 (SPAG5)" ellipses_6 ic Blue fos 15

206 "10664 (CTCF)" ellipses_6 ic Blue fos 15

207 "10891 (PPARGC1A)" diamonds_10 ic White bw 10 fos 15

208 "10946 (SF3A3)" ellipses_6 ic Blue fos 15

209 "10957 (PNRC1)" ellipses_6 ic Red fos 15

210 "11044 (POLS)" ellipses_6 ic Red fos 15

211 "11052 (CPSF6)" ellipses_6 ic White fos 15

212 "11140 (CDC37)" ellipses_6 ic Red fos 15

213 "11156 (PTP4A3)" ellipses_6 ic Red fos 15

214 "11200 (CHEK2)" ellipses_6 ic Blue fos 15

215 "11218 (DDX20)" ellipses_6 ic NavyBlue fos 15

216 "11232 (POLG2)" ellipses_6 ic Blue fos 15

217 "22927 (HABP4)" ellipses_6 ic LightCyan fos 15

218 "23020 (ASCC3L1)" ellipses_6 ic Blue fos 15

219 "23309 (SIN3B)" ellipses_6 ic NavyBlue fos 15

220 "23401 (FRAT2)" ellipses_6 ic Blue fos 15

221 "23450 (SF3B3)" ellipses_6 ic White fos 15

222 "23512 (SUZ12)" ellipses_6 ic LightCyan fos 15

223 "23633 (KPNA6)" ellipses_6 ic White fos 15

224 "23645 (PPP1R15A)" ellipses_6 ic Red fos 15

225 "23708 (GSPT2)" ellipses_6 ic Blue fos 15

226 "23764 (MAFF)" diamonds_10 ic Red bw 10 fos 15

227 "25816 (TNFAIP8)" ellipses_6 ic Red fos 15

228 "25929 (GEMIN5)" ellipses_6 ic Blue fos 15

229 "29127 (RACGAP1)" ellipses_6 ic Blue fos 15

230 "51058 (ZNF691)" ellipses_6 ic Blue fos 15

231 "51191 (HERC5)" ellipses_6 ic Red fos 15

232 "51497 (TH1L)" ellipses_6 ic White fos 15

233 "51499 (TRIAP1)" ellipses_6 ic Blue fos 15

234 "51545 (ZNF581)" ellipses_6 ic Blue fos 15

235 "53615 (MBD3)" ellipses_6 ic Cyan fos 15

236 "54665 (RSBN1)" ellipses_6 ic Blue fos 15

237 "55035 (NOL8)" ellipses_6 ic Blue fos 15

238 "55070 (DET1)" ellipses_6 ic Blue fos 15

239 "55198 (APPL2)" ellipses_6 ic LightCyan fos 15

240 "55207 (ARL8B)" ellipses_6 ic Red fos 15

241 "55367 (LRDD)" ellipses_6 ic NavyBlue fos 15

242 "55602 (CDKN2AIP)" ellipses_6 ic Blue fos 15

243 "57541 (ZNF398)" ellipses_6 ic Blue fos 15

244 "60491 (NIF3L1)" ellipses_6 ic Blue fos 15

245 "63967 (CLSPN)" ellipses_6 ic White fos 15

246 "64395 (GMCL1)" ellipses_6 ic Cyan fos 15

247 "65056 (GPBP1)" ellipses_6 ic Red fos 15

248 "79447 (C16orf53)" ellipses_6 ic Blue fos 15

249 "79724 (ZNF768)" ellipses_6 ic Blue fos 15

250 "79728 (PALB2)" ellipses_6 ic Blue fos 15

251 "79833 (GEMIN6)" ellipses_6 ic Blue fos 15

252 "80851 (SH3BP5L)" ellipses_6 ic Blue fos 15

253 "83593 (RASSF5)" ellipses_6 ic Red fos 15

254 "84078 (KBTBD7)" ellipses_6 ic Blue fos 15

255 "84148 (MYST1)" ellipses_6 ic Blue fos 15

256 "84206 (MEX3B)" ellipses_6 ic Blue fos 15

257 "84246 (MED10)" ellipses_6 ic LightYellow fos 15

258 "84273 (C4orf14)" ellipses_6 ic Blue fos 15

259 "114112 (TXNRD3)" ellipses_6 ic Blue fos 15

260 "115509 (ZNF689)" ellipses_6 ic Blue fos 15

261 "126272 (EID2B)" ellipses_6 ic Blue fos 15

262 "140885 (SIRPA)" triangles_10 ic Red bw 10 fos 15

263 "148327 (CREB3L4)" diamonds_10 ic Blue bw 10 fos 15

264 "149420 (PDIK1L)" ellipses_6 ic Blue fos 15

265 "151246 (SGOL2)" ellipses_6 ic Blue fos 15

266 "170954 (KIAA1949)" ellipses_6 ic Red fos 15

267 "196513 (DCP1B)" ellipses_6 ic Blue fos 15

268 "219541 (MED19)" ellipses_6 ic LightCyan fos 15

269 "221079 (ARL5B)" ellipses_6 ic Red fos 15

270 "255488 (RNF144B)" triangles_10 ic Red bw 10 fos 15

*Arcs

137 115 0.5421148828035831 ap 0 c Lavender

97 161 0.43626516275795535 ap 0 c Blue

55 214 0.2624864799517808 ap 0 c Black

108 66 0.44705573624029277 ap 0 c Blue

2 166 0.6345072259226064 ap 0 c Lavender

247 32 0.63305964790485 ap 0 c Lavender

253 101 0.47099339014679353 ap 0 c Lavender

27 91 1.0 ap 0 c Blue l TF-DNA

6 214 1.0 ap 0 c Lavender

115 81 0.38912356889206706 ap 0 c Blue

118 74 1.0 ap 0 c Lavender l TF-DNA

116 163 0.6510069773088731 ap 0 c Black

16 17 0.554296553707171 ap 0 c Blue

169 161 0.47233330604145624 ap 0 c Lavender

115 80 0.5641625514920002 ap 0 c Lavender

30 142 0.36156212200445986 ap 0 c Blue

30 141 0.3222824047342254 ap 0 c Blue

50 32 0.5374594384852609 ap 0 c Lavender

235 136 0.31749782675870214 ap 0 c Lavender

81 31 0.37694984811917964 ap 0 c Blue

11 95 0.29213691360429156 ap 0 c Lavender

166 2 0.6345072259226064 ap 0 c Lavender

202 221 0.4947771400597229 ap 0 c Black

190 20 0.6126170062362062 ap 0 c Black

149 185 0.4131767740128001 ap 0 c Lavender

185 212 0.5802990291435908 ap 0 c Lavender

118 73 1.0 ap 0 c Lavender l TF-DNA

115 91 1.0 ap 0 c Lavender l TF-DNA

108 128 1.0 ap 0 c Blue

31 81 0.37694984811917964 ap 0 c Blue

34 108 1.0 ap 0 c Blue

19 231 0.4168771745141613 ap 0 c Blue

172 33 0.2844500934331095 ap 0 c Black

56 38 1.0 ap 0 c Lavender l TF-DNA

124 76 0.5885503510983404 ap 0 c Lavender

180 241 0.21239169222143076 ap 0 c Blue

152 154 0.6586379338413391 ap 0 c Blue

160 166 0.43353014708444765 ap 0 c Lavender

70 69 0.318582506754236 ap 0 c Lavender

152 155 0.3134691239277407 ap 0 c Lavender

115 88 1.0 ap 0 c Lavender l TF-DNA

10 106 0.5835243351520584 ap 0 c Lavender

87 97 1.0 ap 0 c Lavender

37 4 0.2963369885101992 ap 0 c Blue

123 106 0.5947570260762174 ap 0 c Lavender

169 163 0.63827257257528 ap 0 c Lavender

229 172 0.6503106268507691 ap 0 c Blue

114 226 0.38621277478940275 ap 0 c Blue

118 72 1.0 ap 0 c Lavender l TF-DNA

130 179 0.26512464579940354 ap 0 c Lavender

163 22 0.6441999141267628 ap 0 c Black

76 116 0.5084296078499602 ap 0 c Lavender

169 162 0.681093985656905 ap 0 c Lavender

152 153 0.6458619663098123 ap 0 c Lavender

111 115 0.13707816654328459 ap 0 c Lavender

80 161 0.4359985311201446 ap 0 c Blue

161 17 0.3716908816166187 ap 0 c Blue

126 186 0.6416984190681595 ap 0 c Lavender

115 193 1.0 ap 0 c Lavender l TF-DNA

166 17 0.5142324271844838 ap 0 c Blue

152 151 0.4901944359783349 ap 0 c Lavender

183 185 0.3538054910003756 ap 0 c Lavender

218 172 0.6142525209669462 ap 0 c Blue

103 269 0.26245123353253424 ap 0 c Lavender

23 150 0.6705901599993618 ap 0 c Lavender

59 40 0.49085808144580906 ap 0 c Lavender

224 125 0.3900633335094465 ap 0 c Lavender

42 126 0.5752281170070164 ap 0 c Black

163 192 1.0 ap 0 c Black l Both

101 253 0.47099339014679353 ap 0 c Lavender

67 2 0.5933229164881911 ap 0 c Blue

56 98 1.0 ap 0 c Lavender l Both

56 20 0.3744360107810151 ap 0 c Lavender

103 61 0.5876833891569492 ap 0 c Lavender

22 163 0.6441999141267628 ap 0 c Lavender l kinase_substrate

181 129 0.6580840863874308 ap 0 c Lavender

117 116 0.5286272717571262 ap 0 c Black

27 116 1.0 ap 0 c Blue l Both

110 258 0.35204170646774474 ap 0 c Blue

117 118 0.4104589424463649 ap 0 c Black

215 251 0.6354893497110092 ap 0 c Lavender

27 117 1.0 ap 0 c Blue l TF-DNA

27 118 1.0 ap 0 c Lavender l TF-DNA

73 147 0.21542483640198576 ap 0 c Black

268 80 0.4819811026412987 ap 0 c Black

3 142 1.0 ap 0 c Blue l TF-DNA

163 248 1.0 ap 0 c Black l TF-DNA

27 88 1.0 ap 0 c Blue l TF-DNA

268 81 0.4485081686806283 ap 0 c Black

268 257 0.40752137614320805 ap 0 c Black

103 111 0.15633008804862547 ap 0 c Lavender

172 18 0.6564136707035352 ap 0 c Black

153 109 0.47492090010509813 ap 0 c Lavender

34 122 0.14095221988048456 ap 0 c Lavender

57 5 0.3924164955115633 ap 0 c Blue

92 97 1.0 ap 0 c Lavender

236 169 0.6137773200237612 ap 0 c Lavender

22 19 0.49646340307721537 ap 0 c Lavender

230 188 0.5211854683824386 ap 0 c Lavender

254 7 0.21717145625743584 ap 0 c Lavender

115 85 1.0 ap 0 c Lavender l TF-DNA

231 19 0.4168771745141613 ap 0 c Blue

137 140 0.4219511033601224 ap 0 c Lavender

214 23 1.0 ap 0 c Lavender

163 164 1.0 ap 0 c Black l Both

22 205 0.3477897789475774 ap 0 c Lavender

264 159 0.2487352328100839 ap 0 c Blue

187 182 0.5407341925951569 ap 0 c Lavender

163 162 0.663973806447376 ap 0 c Black

70 115 0.21849400879123262 ap 0 c Lavender

6 245 0.5745792717376188 ap 0 c Lavender

41 127 1.0 ap 0 c Blue

34 32 0.24571102682394758 ap 0 c Lavender

39 139 0.6850778896568891 ap 0 c Lavender

115 111 0.13707816654328459 ap 0 c Lavender

3 22 1.0 ap 0 c Lavender l TF-DNA

98 56 0.15582913603252696 ap 0 c Lavender

137 223 0.2887322884652811 ap 0 c Lavender

12 106 0.4534140726429272 ap 0 c Lavender

151 153 0.4750706102070733 ap 0 c Lavender

257 81 0.4242800933742042 ap 0 c Black

147 1 0.3397497908727391 ap 0 c Black

228 176 0.6543495325842783 ap 0 c Lavender

251 176 0.6667631637949012 ap 0 c Lavender

151 152 0.4901944359783349 ap 0 c Lavender

6 112 0.6410871080730496 ap 0 c Blue

61 97 1.0 ap 0 c Lavender l TF-DNA

115 98 1.0 ap 0 c Lavender l TF-DNA

172 108 0.45179642944708737 ap 0 c Black

130 259 0.22791099084736105 ap 0 c Blue

39 34 0.3430524952511575 ap 0 c Blue

232 190 0.6398053487441339 ap 0 c Lavender

163 204 0.6353572223199553 ap 0 c Black

19 112 0.5661079171655526 ap 0 c Blue

126 125 0.6727989908934118 ap 0 c Lavender

124 88 1.0 ap 0 c Lavender l TF-DNA

115 2 1.0 ap 0 c Lavender l TF-DNA

100 32 0.21358086483587532 ap 0 c Lavender

32 3 0.5884893435563874 ap 0 c Lavender

163 150 0.681995891743701 ap 0 c Black

28 32 0.4738106606672744 ap 0 c Lavender

60 95 0.4653538387713733 ap 0 c Lavender

182 241 0.516999887872986 ap 0 c Lavender

163 173 1.0 ap 0 c Black l TF-DNA

124 187 1.0 ap 0 c Lavender l TF-DNA

189 185 0.3806836101608239 ap 0 c Blue

85 86 1.0 ap 0 c Lavender

113 26 0.45843544270982556 ap 0 c Lavender

117 73 1.0 ap 0 c Black l TF-DNA

151 154 0.4608697672731732 ap 0 c Blue

154 221 0.6530361759518899 ap 0 c Black

151 155 0.3478148932404389 ap 0 c Lavender

191 25 1.0 ap 0 c Blue

215 213 0.39832172988096476 ap 0 c Blue

163 169 0.63827257257528 ap 0 c Black

268 24 0.44376857546092735 ap 0 c Black

23 32 0.555322060169378 ap 0 c Lavender

172 218 0.6142525209669462 ap 0 c Black

4 163 1.0 ap 0 c Blue l Both

124 193 1.0 ap 0 c Lavender l TF-DNA

117 72 1.0 ap 0 c Black l TF-DNA

49 18 0.3047383224689104 ap 0 c Blue

8 10 0.4703063612892095 ap 0 c Lavender

61 209 1.0 ap 0 c Lavender l TF-DNA

69 136 0.31391637585307947 ap 0 c Black

163 20 1.0 ap 0 c Black l Both

169 236 0.6137773200237612 ap 0 c Lavender

268 215 0.6242863318701964 ap 0 c Black

71 42 0.5518757794336856 ap 0 c Blue

104 172 0.6255829088008602 ap 0 c Black

235 70 0.3255175989652347 ap 0 c Lavender

141 30 0.3222824047342254 ap 0 c Blue

117 75 1.0 ap 0 c Black l TF-DNA

106 165 0.6000898986950852 ap 0 c Lavender

206 103 0.6570995921483023 ap 0 c Lavender

10 17 0.5499907027034969 ap 0 c Blue

8 12 0.3573563714281889 ap 0 c Lavender

162 77 0.6768081510254423 ap 0 c Black

27 157 1.0 ap 0 c Lavender l TF-DNA

179 130 0.26512464579940354 ap 0 c Lavender

47 49 1.0 ap 0 c Lavender

10 16 0.6487923433000795 ap 0 c Blue

117 74 1.0 ap 0 c Black l TF-DNA

113 163 0.6610321976313341 ap 0 c Lavender

182 185 0.5027584480562084 ap 0 c Blue

53 22 0.47330803038036895 ap 0 c Black

36 22 1.0 ap 0 c Lavender

163 159 1.0 ap 0 c Black l TF-DNA

113 162 0.6739187982188775 ap 0 c Lavender

56 111 0.13258597642416625 ap 0 c Lavender

162 145 0.691980940204597 ap 0 c Black

115 141 1.0 ap 0 c Lavender l TF-DNA

115 142 1.0 ap 0 c Lavender l TF-DNA

18 49 0.3047383224689104 ap 0 c Blue

98 26 1.0 ap 0 c Lavender l TF-DNA

7 188 0.18228596703668107 ap 0 c Lavender

192 140 0.4175695946798654 ap 0 c Lavender

139 39 0.6850778896568891 ap 0 c Lavender

81 267 0.5441838786524129 ap 0 c Lavender

34 169 0.3180547630528444 ap 0 c Lavender

148 147 0.3846504947407948 ap 0 c Lavender

81 257 0.4242800933742042 ap 0 c Blue

50 106 1.0 ap 0 c Lavender l TF-DNA

39 57 0.6814339244948303 ap 0 c Blue

76 22 0.5149633562574866 ap 0 c Lavender

115 143 1.0 ap 0 c Lavender l TF-DNA

32 34 0.24571102682394758 ap 0 c Lavender

132 56 0.1439143729853139 ap 0 c Lavender

157 200 0.5649920089388739 ap 0 c Lavender

251 215 0.6354893497110092 ap 0 c Lavender

34 163 0.345315545249255 ap 0 c Blue

147 148 0.3846504947407948 ap 0 c Black

162 113 0.6739187982188775 ap 0 c Black

126 89 0.2481138515672485 ap 0 c Blue

18 168 0.6736359903387633 ap 0 c Blue

225 67 0.2599273262919289 ap 0 c Lavender

4 98 0.29033010754979977 ap 0 c Lavender

123 8 0.45183382828324836 ap 0 c Lavender

70 39 0.3363650034627969 ap 0 c Lavender

70 235 0.3255175989652347 ap 0 c Lavender

268 35 0.5091338319254661 ap 0 c Black

115 158 1.0 ap 0 c Lavender l TF-DNA

202 208 0.3447089120552874 ap 0 c Black

27 32 0.3926874934411427 ap 0 c Lavender

208 202 0.3447089120552874 ap 0 c Blue

217 224 0.16947975008542498 ap 0 c Black

17 161 0.3716908816166187 ap 0 c Black

226 114 0.38621277478940275 ap 0 c Blue

88 147 0.12095461996870945 ap 0 c Blue

150 125 0.6778269061899242 ap 0 c Lavender

154 69 0.6174140218692834 ap 0 c Black

80 166 0.3708991815483336 ap 0 c Lavender

86 182 1.0 ap 0 c Blue

7 22 0.302404749010349 ap 0 c Lavender

167 34 0.33860245109983195 ap 0 c Lavender

118 75 1.0 ap 0 c Lavender l TF-DNA

163 125 0.6808143657499419 ap 0 c Black

115 50 1.0 ap 0 c Lavender l TF-DNA

207 113 0.3832731015883574 ap 0 c Lavender

23 56 0.3972230435041284 ap 0 c Lavender

6 171 0.47088197060911985 ap 0 c Lavender

180 80 0.30065973649117494 ap 0 c Lavender

163 6 1.0 ap 0 c Black l Both

57 39 0.6814339244948303 ap 0 c Blue

20 24 0.6418143347404589 ap 0 c Lavender

180 81 0.1373998981440491 ap 0 c Blue

56 23 0.3972230435041284 ap 0 c Lavender

213 80 0.38271164243875133 ap 0 c Lavender

16 187 0.540354003991133 ap 0 c Lavender

22 76 0.5149633562574866 ap 0 c Lavender l kinase_substrate

171 6 0.47088197060911985 ap 0 c Lavender

161 97 0.43626516275795535 ap 0 c Blue

241 182 0.516999887872986 ap 0 c Lavender

174 42 1.0 ap 0 c Blue

116 76 0.5084296078499602 ap 0 c Black

113 260 0.546380092609623 ap 0 c Lavender

163 121 1.0 ap 0 c Black l TF-DNA

77 101 0.6073182918731387 ap 0 c Lavender

113 207 0.3832731015883574 ap 0 c Lavender

172 252 0.6578424414042975 ap 0 c Black

163 127 1.0 ap 0 c Black l Both

185 189 0.3806836101608239 ap 0 c Blue

214 6 0.6189598143252396 ap 0 c Lavender

81 180 0.1373998981440491 ap 0 c Blue

103 56 1.0 ap 0 c Lavender l Both

28 85 1.0 ap 0 c Lavender l TF-DNA

224 217 0.16947975008542498 ap 0 c Blue

3 216 1.0 ap 0 c Lavender l TF-DNA

125 163 0.6808143657499419 ap 0 c Lavender

28 88 1.0 ap 0 c Lavender l TF-DNA

22 192 0.5291881382364844 ap 0 c Lavender

56 124 1.0 ap 0 c Lavender l TF-DNA

115 31 1.0 ap 0 c Lavender l Both

125 164 0.5603684175621613 ap 0 c Lavender

27 142 1.0 ap 0 c Blue l TF-DNA

188 100 0.18219385533303775 ap 0 c Lavender

7 254 0.21717145625743584 ap 0 c Lavender

153 151 0.4750706102070733 ap 0 c Lavender

70 105 0.31305106251766535 ap 0 c Lavender

125 150 0.6778269061899242 ap 0 c Lavender

5 165 0.32837563879355003 ap 0 c Black

153 152 0.6458619663098123 ap 0 c Lavender

257 24 0.4543895701383582 ap 0 c Black

34 185 0.22976599424946068 ap 0 c Lavender

56 132 0.1439143729853139 ap 0 c Lavender

163 7 0.2896792965626264 ap 0 c Black

35 238 0.5791240263803448 ap 0 c Lavender

70 136 0.31999759412377843 ap 0 c Lavender

26 45 0.4726293060279612 ap 0 c Lavender

94 76 0.3879109084324568 ap 0 c Lavender

182 183 0.6861485640624241 ap 0 c Blue

181 158 0.5268663019349323 ap 0 c Lavender

126 265 0.6315932447925552 ap 0 c Blue

161 182 1.0 ap 0 c Lavender

115 186 1.0 ap 0 c Lavender l TF-DNA

185 181 0.5681167435304667 ap 0 c Lavender

127 98 0.1570017092312279 ap 0 c Black

185 182 1.0 ap 0 c Blue

52 51 0.4940536837443621 ap 0 c Blue

163 187 1.0 ap 0 c Black l TF-DNA

192 195 1.0 ap 0 c Lavender

49 47 0.1427128397891996 ap 0 c Lavender

163 34 0.345315545249255 ap 0 c Black

49 48 0.22783803761163635 ap 0 c Blue

124 127 0.5657128348632011 ap 0 c Lavender

260 113 0.546380092609623 ap 0 c Lavender

185 180 0.2438557546958054 ap 0 c Lavender

132 96 1.0 ap 0 c Blue

63 42 1.0 ap 0 c Blue

160 172 0.4368329125278328 ap 0 c Blue

171 55 0.301486935994481 ap 0 c Blue

22 125 0.5006292343655876 ap 0 c Lavender

112 6 0.6410871080730496 ap 0 c Blue

45 26 0.4726293060279612 ap 0 c Lavender

106 12 0.4534140726429272 ap 0 c Lavender

192 80 0.4759318946808244 ap 0 c Lavender

169 135 0.6246766814828026 ap 0 c Lavender

25 191 1.0 ap 0 c Blue

241 180 0.21239169222143076 ap 0 c Blue

32 68 0.2859605895816904 ap 0 c Lavender

92 91 0.27695386181192194 ap 0 c Lavender

17 182 0.5209375402068913 ap 0 c Black

56 243 1.0 ap 0 c Lavender l Both

172 96 0.6169477686643221 ap 0 c Black

180 185 0.2438557546958054 ap 0 c Lavender

9 137 0.44854600338141004 ap 0 c Blue

163 241 1.0 ap 0 c Black l TF-DNA

34 39 0.3430524952511575 ap 0 c Blue

88 78 0.2628852640574428 ap 0 c Blue

124 36 1.0 ap 0 c Lavender l TF-DNA

150 23 0.6705901599993618 ap 0 c Lavender

70 139 0.3550420437552346 ap 0 c Lavender

217 34 0.20111684333888633 ap 0 c Black

65 172 0.46415513877658937 ap 0 c Blue

80 192 0.4759318946808244 ap 0 c Lavender

268 237 0.42709386671549676 ap 0 c Black

161 181 0.4297287999608643 ap 0 c Blue

103 83 1.0 ap 0 c Lavender l TF-DNA

5 161 0.5603478405235656 ap 0 c Black

22 7 0.302404749010349 ap 0 c Lavender

207 56 0.3421337484366975 ap 0 c Lavender

100 268 0.32422337387848726 ap 0 c Lavender

222 57 0.6660336872645332 ap 0 c Black

81 161 0.23263288159888162 ap 0 c Lavender

163 111 0.14436105540750013 ap 0 c Black

32 84 1.0 ap 0 c Lavender l TF-DNA

105 169 0.6525415664318799 ap 0 c Black

161 180 1.0 ap 0 c Blue

163 41 1.0 ap 0 c Black l TF-DNA

169 50 0.641153329641437 ap 0 c Lavender

256 103 0.5072818661541781 ap 0 c Lavender

202 218 0.4931008662094727 ap 0 c Black

120 207 0.4163322820079477 ap 0 c Lavender

153 155 0.2976776882129614 ap 0 c Lavender

153 154 0.6408741039707171 ap 0 c Blue

32 70 0.24714132713367798 ap 0 c Lavender

148 18 0.43200009201272604 ap 0 c Lavender

96 127 0.47794095036114737 ap 0 c Black

163 37 1.0 ap 0 c Black l TF-DNA

145 176 0.6791625483791727 ap 0 c Lavender

163 36 1.0 ap 0 c Black l TF-DNA

192 163 0.675692893537361 ap 0 c Lavender

115 58 1.0 ap 0 c Lavender l TF-DNA

163 26 0.4117892269481872 ap 0 c Black

122 34 0.14095221988048456 ap 0 c Lavender

70 57 0.32824894045605774 ap 0 c Lavender

4 37 0.2963369885101992 ap 0 c Blue

197 125 0.41544840253241494 ap 0 c Lavender

181 103 0.612514790002361 ap 0 c Lavender

78 88 0.2628852640574428 ap 0 c Blue

34 70 0.34688630084381855 ap 0 c Lavender

106 10 0.5835243351520584 ap 0 c Lavender

115 185 0.6565326110186523 ap 0 c Lavender

205 22 0.3477897789475774 ap 0 c Lavender

215 176 0.6141254554149878 ap 0 c Lavender

120 144 1.0 ap 0 c Lavender l TF-DNA

161 5 0.5603478405235656 ap 0 c Blue

4 142 1.0 ap 0 c Lavender l TF-DNA

24 268 0.44376857546092735 ap 0 c Blue

32 247 0.63305964790485 ap 0 c Lavender

13 56 1.0 ap 0 c Lavender l TF-DNA

183 182 1.0 ap 0 c Blue

3 32 0.5884893435563874 ap 0 c Lavender

22 53 0.47330803038036895 ap 0 c Lavender

32 100 0.21358086483587532 ap 0 c Lavender

201 172 0.5614490549685793 ap 0 c Blue

13 163 0.6387506456940936 ap 0 c Lavender

122 50 1.0 ap 0 c Lavender l TF-DNA

81 163 0.3925393857563809 ap 0 c Blue

112 214 0.5685967057091559 ap 0 c Blue

163 126 0.6656638590792652 ap 0 c Black

220 42 1.0 ap 0 c Blue

50 169 0.641153329641437 ap 0 c Lavender

188 110 0.5308373717631789 ap 0 c Lavender

194 32 1.0 ap 0 c Lavender

87 189 0.5675658451154768 ap 0 c Lavender

30 97 0.32463296552328597 ap 0 c Blue

125 224 0.3900633335094465 ap 0 c Lavender

43 262 1.0 ap 0 c Lavender l TF-DNA

172 201 0.5614490549685793 ap 0 c Black

163 27 1.0 ap 0 c Black l TF-DNA

77 162 0.6768081510254423 ap 0 c Blue

103 40 1.0 ap 0 c Lavender l TF-DNA

242 26 0.4456683824561266 ap 0 c Lavender

56 207 0.3421337484366975 ap 0 c Lavender

76 32 0.3205454476663399 ap 0 c Lavender

212 185 0.5802990291435908 ap 0 c Lavender

103 32 1.0 ap 0 c Lavender

87 96 0.3256528871564077 ap 0 c Lavender

87 204 0.35440531218988297 ap 0 c Lavender

267 103 0.615377871756672 ap 0 c Lavender

126 42 0.5752281170070164 ap 0 c Blue

190 70 0.3005766089773699 ap 0 c Black

81 115 0.38912356889206706 ap 0 c Blue

257 268 0.40752137614320805 ap 0 c Black

211 137 0.4533707651700622 ap 0 c Lavender

163 43 1.0 ap 0 c Black l TF-DNA

109 153 0.47492090010509813 ap 0 c Lavender

53 135 0.5512521536694577 ap 0 c Black

163 45 0.5582326568644927 ap 0 c Black

59 87 0.33890662272904204 ap 0 c Lavender

163 100 0.34151040575979275 ap 0 c Black

190 163 0.60857036686755 ap 0 c Black

163 214 1.0 ap 0 c Black l Both

238 35 0.5791240263803448 ap 0 c Lavender

198 268 0.40229986687112657 ap 0 c Black

54 55 0.2381277587718034 ap 0 c Blue

163 123 1.0 ap 0 c Black l TF-DNA

35 268 0.5091338319254661 ap 0 c Lavender

59 97 0.45463781922717805 ap 0 c Blue

61 103 1.0 ap 0 c Lavender l Both

122 32 0.1407426966309886 ap 0 c Lavender

96 132 0.29452583726298337 ap 0 c Black

145 162 0.691980940204597 ap 0 c Lavender

4 21 1.0 ap 0 c Lavender l TF-DNA

132 66 0.25595266216377655 ap 0 c Lavender

155 152 0.3134691239277407 ap 0 c Lavender

155 153 0.2976776882129614 ap 0 c Lavender

72 74 0.664388272121303 ap 0 c Lavender

155 151 0.3478148932404389 ap 0 c Lavender

234 77 0.6526968860041908 ap 0 c Blue

40 59 0.49085808144580906 ap 0 c Lavender

50 83 0.25860718525276394 ap 0 c Lavender

28 159 1.0 ap 0 c Lavender l TF-DNA

68 154 0.33100953321235527 ap 0 c Blue

218 162 0.6406688232995487 ap 0 c Blue

26 67 0.49723864360162123 ap 0 c Lavender

32 114 0.399843204766859 ap 0 c Lavender

172 10 0.6469633768256914 ap 0 c Black

155 154 0.3557731453975958 ap 0 c Lavender

190 232 0.6398053487441339 ap 0 c Black

163 217 0.17525542809669736 ap 0 c Black

136 70 0.31999759412377843 ap 0 c Lavender

69 207 0.39532706658130257 ap 0 c Black

32 23 0.555322060169378 ap 0 c Lavender

127 146 0.5842110493932429 ap 0 c Black

257 198 0.40954354149966365 ap 0 c Black

67 26 0.49723864360162123 ap 0 c Lavender

50 159 1.0 ap 0 c Lavender l TF-DNA

81 166 0.3933367865629602 ap 0 c Blue

43 222 1.0 ap 0 c Lavender l TF-DNA

158 181 0.5268663019349323 ap 0 c Lavender

185 183 0.3538054910003756 ap 0 c Lavender

56 61 0.43878199996804446 ap 0 c Lavender

37 88 1.0 ap 0 c Blue l TF-DNA

27 50 1.0 ap 0 c Lavender l TF-DNA

97 96 0.38866327906115083 ap 0 c Blue

268 198 0.40229986687112657 ap 0 c Black

52 204 0.6392079948680434 ap 0 c Lavender

50 178 1.0 ap 0 c Lavender l TF-DNA

54 53 0.5029310702866125 ap 0 c Blue

136 69 0.31391637585307947 ap 0 c Lavender

70 261 0.22518943995382226 ap 0 c Lavender

103 181 0.612514790002361 ap 0 c Lavender

128 50 0.6344567970808227 ap 0 c Lavender

169 34 0.3180547630528444 ap 0 c Lavender

181 185 0.5681167435304667 ap 0 c Lavender

243 56 0.43670599183326725 ap 0 c Lavender

163 57 1.0 ap 0 c Black l Both

147 88 0.12095461996870945 ap 0 c Black

42 71 0.5518757794336856 ap 0 c Black

136 235 0.31749782675870214 ap 0 c Lavender

53 54 0.5029310702866125 ap 0 c Black

56 163 0.42203007696081735 ap 0 c Lavender

190 136 0.28035121465562995 ap 0 c Black

115 133 1.0 ap 0 c Lavender l TF-DNA

32 163 1.0 ap 0 c Lavender l Both

22 23 0.6653329510579603 ap 0 c Lavender l kinase_substrate

53 163 0.6616150487831145 ap 0 c Black

120 64 1.0 ap 0 c Blue l TF-DNA

163 210 1.0 ap 0 c Black l TF-DNA

56 32 0.5016823212910863 ap 0 c Lavender

90 97 1.0 ap 0 c Lavender

100 137 0.28544738182908336 ap 0 c Lavender

122 56 1.0 ap 0 c Lavender l TF-DNA

76 124 0.5885503510983404 ap 0 c Lavender

116 27 0.38139628900243316 ap 0 c Black

1 147 0.3397497908727391 ap 0 c Blue

188 230 0.5211854683824386 ap 0 c Lavender

172 23 1.0 ap 0 c Black

44 222 1.0 ap 0 c Blue l TF-DNA

25 19 1.0 ap 0 c Lavender

56 115 0.39505959444811334 ap 0 c Lavender

115 84 1.0 ap 0 c Lavender l TF-DNA

172 25 0.5319136570679897 ap 0 c Black

140 137 0.4219511033601224 ap 0 c Lavender

136 190 0.28035121465562995 ap 0 c Lavender

100 122 0.14340865695015392 ap 0 c Lavender

97 30 0.32463296552328597 ap 0 c Blue

163 203 0.3280349149315121 ap 0 c Black

137 100 0.28544738182908336 ap 0 c Lavender

7 163 0.2896792965626264 ap 0 c Blue

103 98 0.19255827366073094 ap 0 c Lavender

218 202 0.4931008662094727 ap 0 c Blue

56 209 1.0 ap 0 c Lavender l TF-DNA

146 202 0.5925424825938719 ap 0 c Black

16 185 0.5397215786220453 ap 0 c Blue

169 105 0.6525415664318799 ap 0 c Lavender

98 127 0.1570017092312279 ap 0 c Blue

187 16 0.540354003991133 ap 0 c Lavender

115 183 1.0 ap 0 c Lavender l TF-DNA

98 103 1.0 ap 0 c Lavender l Both

103 79 0.3961467453825637 ap 0 c Lavender

164 125 0.5603684175621613 ap 0 c Lavender

100 163 0.34151040575979275 ap 0 c Lavender

187 244 0.40136898235251883 ap 0 c Lavender

135 169 0.6246766814828026 ap 0 c Lavender

221 202 0.4947771400597229 ap 0 c Lavender

69 70 0.318582506754236 ap 0 c Black

32 122 0.1407426966309886 ap 0 c Lavender

66 132 0.25595266216377655 ap 0 c Lavender

76 94 0.3879109084324568 ap 0 c Lavender

19 22 0.49646340307721537 ap 0 c Lavender

27 124 1.0 ap 0 c Lavender l TF-DNA

3 159 1.0 ap 0 c Blue l TF-DNA

163 82 1.0 ap 0 c Black l TF-DNA

26 130 0.34673184528070666 ap 0 c Lavender

127 50 1.0 ap 0 c Black

32 103 0.6472531175943291 ap 0 c Lavender

125 126 0.6727989908934118 ap 0 c Lavender

28 27 0.4652042521107459 ap 0 c Lavender

87 59 0.33890662272904204 ap 0 c Lavender

139 70 0.3550420437552346 ap 0 c Lavender

95 60 0.4653538387713733 ap 0 c Lavender

232 80 0.4965763805788062 ap 0 c Lavender

39 70 0.3363650034627969 ap 0 c Lavender

56 190 0.4311699886375525 ap 0 c Lavender

198 24 0.38834365992930225 ap 0 c Black

56 187 1.0 ap 0 c Lavender l TF-DNA

109 117 0.44642315790407594 ap 0 c Blue

110 188 0.5308373717631789 ap 0 c Lavender

262 187 0.23304629068643554 ap 0 c Blue

96 97 0.38866327906115083 ap 0 c Black

116 117 0.5286272717571262 ap 0 c Black

116 118 0.4896275848806323 ap 0 c Black

157 134 0.20520734666979773 ap 0 c Lavender

10 172 0.6469633768256914 ap 0 c Blue

24 257 0.4543895701383582 ap 0 c Blue

172 160 0.4368329125278328 ap 0 c Black

150 163 0.681995891743701 ap 0 c Blue

56 170 0.45272198920994144 ap 0 c Lavender

250 13 0.6295970447700182 ap 0 c Lavender

80 268 0.4819811026412987 ap 0 c Blue

56 156 0.2518116969441986 ap 0 c Lavender

81 268 0.4485081686806283 ap 0 c Blue

166 185 0.4508464427741266 ap 0 c Lavender

232 103 0.6249815043037912 ap 0 c Lavender

122 100 0.14340865695015392 ap 0 c Lavender

169 249 0.569075560489231 ap 0 c Lavender

57 44 0.492127960709823 ap 0 c Blue

32 56 0.5016823212910863 ap 0 c Lavender

79 103 0.3961467453825637 ap 0 c Lavender

66 108 0.44705573624029277 ap 0 c Blue

43 19 1.0 ap 0 c Lavender l TF-DNA

95 11 0.29213691360429156 ap 0 c Lavender

249 169 0.569075560489231 ap 0 c Lavender

172 104 0.6255829088008602 ap 0 c Black

27 96 1.0 ap 0 c Blue l TF-DNA

48 49 1.0 ap 0 c Blue

176 251 0.6667631637949012 ap 0 c Lavender

240 203 0.28512793439135264 ap 0 c Lavender

100 214 0.28941499007057625 ap 0 c Lavender

28 142 1.0 ap 0 c Lavender l TF-DNA

116 157 1.0 ap 0 c Black l TF-DNA

163 233 0.6441021104625074 ap 0 c Black

117 163 0.5174651237673246 ap 0 c Black

34 29 0.2869989024383434 ap 0 c Blue

187 262 0.23304629068643554 ap 0 c Blue

176 215 0.6141254554149878 ap 0 c Lavender

69 154 0.6174140218692834 ap 0 c Black

156 75 0.48013602704804886 ap 0 c Lavender

115 70 0.21849400879123262 ap 0 c Lavender

162 218 0.6406688232995487 ap 0 c Black

91 92 1.0 ap 0 c Lavender

59 96 0.3842672280172877 ap 0 c Blue

50 2 1.0 ap 0 c Lavender l TF-DNA

163 190 0.60857036686755 ap 0 c Black

170 56 0.45272198920994144 ap 0 c Lavender

137 9 0.44854600338141004 ap 0 c Blue

180 166 0.2244500878594139 ap 0 c Lavender

156 56 0.2518116969441986 ap 0 c Lavender

24 198 0.38834365992930225 ap 0 c Blue

70 219 0.2389055568990938 ap 0 c Lavender

166 80 0.3708991815483336 ap 0 c Lavender

100 188 0.18219385533303775 ap 0 c Lavender

166 81 0.3933367865629602 ap 0 c Blue

115 166 1.0 ap 0 c Lavender l TF-DNA

221 154 0.6530361759518899 ap 0 c Lavender

57 163 0.658017594712802 ap 0 c Blue

17 10 0.5499907027034969 ap 0 c Black

176 228 0.6543495325842783 ap 0 c Lavender

115 93 1.0 ap 0 c Lavender l TF-DNA

17 185 0.5599753548903221 ap 0 c Black

183 180 0.330459387196758 ap 0 c Lavender

184 86 0.26793645517040154 ap 0 c Lavender

150 26 0.4488942124224344 ap 0 c Lavender

115 21 1.0 ap 0 c Lavender l TF-DNA

70 196 0.28064033739024885 ap 0 c Lavender

214 112 0.5685967057091559 ap 0 c Blue

29 34 0.2869989024383434 ap 0 c Blue

163 19 1.0 ap 0 c Black l TF-DNA

107 110 0.3674257974002626 ap 0 c Blue

204 163 0.6353572223199553 ap 0 c Lavender

28 58 1.0 ap 0 c Lavender l TF-DNA

61 56 0.43878199996804446 ap 0 c Lavender

163 70 0.3429908479684564 ap 0 c Black

163 88 1.0 ap 0 c Black l TF-DNA

56 103 1.0 ap 0 c Lavender l Both

176 145 0.6791625483791727 ap 0 c Lavender

163 13 1.0 ap 0 c Black l Both

122 163 1.0 ap 0 c Lavender l TF-DNA

226 120 0.5665932439474632 ap 0 c Blue

129 181 0.6580840863874308 ap 0 c Lavender

80 232 0.4965763805788062 ap 0 c Lavender

163 113 0.6610321976313341 ap 0 c Black

221 185 0.3782557683442009 ap 0 c Lavender

16 241 0.5289412432354668 ap 0 c Lavender

50 128 0.6344567970808227 ap 0 c Lavender

188 7 0.18228596703668107 ap 0 c Lavender

67 225 0.2599273262919289 ap 0 c Lavender

115 94 1.0 ap 0 c Lavender l Both

163 12 1.0 ap 0 c Black l TF-DNA

163 270 1.0 ap 0 c Black l TF-DNA

34 167 0.33860245109983195 ap 0 c Lavender

161 166 0.3074564285921982 ap 0 c Blue

86 262 0.3896488787389444 ap 0 c Lavender

116 75 1.0 ap 0 c Black l TF-DNA

204 52 0.6392079948680434 ap 0 c Lavender

115 156 1.0 ap 0 c Lavender l TF-DNA

165 5 0.32837563879355003 ap 0 c Blue

244 187 0.40136898235251883 ap 0 c Lavender

124 131 1.0 ap 0 c Lavender l TF-DNA

61 227 1.0 ap 0 c Lavender l TF-DNA

124 207 1.0 ap 0 c Lavender l Both

182 187 0.5407341925951569 ap 0 c Lavender

110 107 0.3674257974002626 ap 0 c Blue

204 87 0.35440531218988297 ap 0 c Lavender

163 85 1.0 ap 0 c Black l TF-DNA

44 57 0.492127960709823 ap 0 c Blue

45 163 0.5582326568644927 ap 0 c Blue

100 16 0.3191177498796565 ap 0 c Lavender

217 51 0.321858932256685 ap 0 c Black

167 39 0.668500891921627 ap 0 c Lavender

116 74 1.0 ap 0 c Black l TF-DNA

157 129 0.5702443791350399 ap 0 c Lavender

203 240 0.28512793439135264 ap 0 c Lavender

120 226 0.5665932439474632 ap 0 c Blue

159 264 0.2487352328100839 ap 0 c Blue

96 59 0.3842672280172877 ap 0 c Black

80 213 0.38271164243875133 ap 0 c Lavender

140 192 0.4175695946798654 ap 0 c Lavender

70 190 0.3005766089773699 ap 0 c Lavender

163 185 1.0 ap 0 c Black l TF-DNA

163 167 0.6734999111884129 ap 0 c Black

163 81 0.3925393857563809 ap 0 c Black

163 2 1.0 ap 0 c Black l TF-DNA

116 73 1.0 ap 0 c Black l TF-DNA

115 95 1.0 ap 0 c Lavender l TF-DNA

118 117 0.4104589424463649 ap 0 c Lavender

235 150 0.6488369437046476 ap 0 c Lavender

70 34 0.34688630084381855 ap 0 c Lavender

22 1 0.12140136691684811 ap 0 c Lavender l kinase_substrate

23 22 1.0 ap 0 c Lavender

181 81 0.40040032189249264 ap 0 c Blue

115 4 1.0 ap 0 c Lavender l Both

25 172 0.5319136570679897 ap 0 c Blue

245 6 0.5745792717376188 ap 0 c Lavender

118 116 0.4896275848806323 ap 0 c Lavender

27 36 1.0 ap 0 c Blue l TF-DNA

182 180 0.323610760976149 ap 0 c Blue

192 22 0.5291881382364844 ap 0 c Lavender

252 172 0.6578424414042975 ap 0 c Blue

115 180 1.0 ap 0 c Lavender l TF-DNA

31 115 0.5215691845184668 ap 0 c Lavender

103 256 0.5072818661541781 ap 0 c Lavender

115 36 1.0 ap 0 c Lavender l TF-DNA

116 72 1.0 ap 0 c Black l TF-DNA

105 177 0.6020502501014634 ap 0 c Black

145 116 0.6685933656663615 ap 0 c Lavender

32 50 0.5374594384852609 ap 0 c Lavender

24 20 0.6418143347404589 ap 0 c Lavender

262 86 0.3896488787389444 ap 0 c Lavender

207 124 0.37085644516366706 ap 0 c Lavender

180 161 0.2877824923962609 ap 0 c Blue

138 137 0.41237134880644727 ap 0 c Blue

185 34 0.22976599424946068 ap 0 c Lavender

166 161 0.3074564285921982 ap 0 c Blue

111 32 0.1539229934592591 ap 0 c Lavender

163 154 1.0 ap 0 c Black l Both

137 138 0.41237134880644727 ap 0 c Blue

213 215 0.39832172988096476 ap 0 c Blue

102 115 0.40649221556106097 ap 0 c Blue

127 124 0.5657128348632011 ap 0 c Black

180 160 0.20386964523350545 ap 0 c Lavender

180 159 0.15015862518025225 ap 0 c Lavender

50 99 1.0 ap 0 c Lavender l TF-DNA

57 70 0.32824894045605774 ap 0 c Lavender

196 70 0.28064033739024885 ap 0 c Lavender

215 228 0.6016102937555475 ap 0 c Lavender

185 221 0.3782557683442009 ap 0 c Lavender

163 186 1.0 ap 0 c Black l TF-DNA

27 3 1.0 ap 0 c Lavender l TF-DNA

168 18 0.6736359903387633 ap 0 c Blue

39 235 0.6580825508837854 ap 0 c Lavender

70 163 0.3429908479684564 ap 0 c Lavender

185 149 0.4131767740128001 ap 0 c Lavender

214 163 1.0 ap 0 c Blue

266 125 0.507739653032172 ap 0 c Lavender

166 160 0.43353014708444765 ap 0 c Lavender

136 239 0.22664008137896272 ap 0 c Lavender

235 39 0.6580825508837854 ap 0 c Lavender

127 96 1.0 ap 0 c Black

6 163 1.0 ap 0 c Blue

161 159 0.3920217594792542 ap 0 c Blue

69 268 0.6506071325890367 ap 0 c Black

17 16 0.554296553707171 ap 0 c Black

26 242 0.4456683824561266 ap 0 c Lavender

1 22 0.12140136691684811 ap 0 c Lavender

172 229 0.6503106268507691 ap 0 c Black

180 183 0.330459387196758 ap 0 c Lavender

140 188 0.46459061949426445 ap 0 c Lavender

103 232 0.6249815043037912 ap 0 c Lavender

34 217 0.20111684333888633 ap 0 c Blue

125 197 0.41544840253241494 ap 0 c Lavender

20 190 0.6126170062362062 ap 0 c Lavender

200 157 0.5649920089388739 ap 0 c Lavender

115 181 1.0 ap 0 c Lavender l TF-DNA

18 97 1.0 ap 0 c Blue

163 15 1.0 ap 0 c Black l TF-DNA

135 53 0.5512521536694577 ap 0 c Lavender

167 163 0.6734999111884129 ap 0 c Lavender

68 32 0.2859605895816904 ap 0 c Lavender

51 52 0.4940536837443621 ap 0 c Blue

214 100 0.28941499007057625 ap 0 c Lavender

5 57 0.3924164955115633 ap 0 c Black

18 148 0.43200009201272604 ap 0 c Lavender

214 55 0.2624864799517808 ap 0 c Blue

109 74 1.0 ap 0 c Blue l TF-DNA

163 4 1.0 ap 0 c Black l Both

162 169 0.681093985656905 ap 0 c Black

180 182 0.323610760976149 ap 0 c Blue

202 146 0.5925424825938719 ap 0 c Black

18 147 0.3868365918960931 ap 0 c Blue

207 120 0.4163322820079477 ap 0 c Lavender

125 266 0.507739653032172 ap 0 c Lavender

115 137 1.0 ap 0 c Lavender l Both

125 22 0.5006292343655876 ap 0 c Lavender

196 219 0.5829815111301031 ap 0 c Lavender

109 75 1.0 ap 0 c Blue l TF-DNA

37 183 1.0 ap 0 c Blue l TF-DNA

163 32 0.5404486627383511 ap 0 c Black

154 68 0.33100953321235527 ap 0 c Black

115 187 1.0 ap 0 c Blue l TF-DNA

32 82 1.0 ap 0 c Lavender l TF-DNA

161 80 0.4359985311201446 ap 0 c Blue

80 115 0.5641625514920002 ap 0 c Lavender

146 127 0.5842110493932429 ap 0 c Black

258 110 0.35204170646774474 ap 0 c Blue

112 19 0.5661079171655526 ap 0 c Blue

161 81 0.23263288159888162 ap 0 c Lavender

162 163 0.663973806447376 ap 0 c Black

26 150 0.4488942124224344 ap 0 c Lavender

117 157 1.0 ap 0 c Black l TF-DNA

246 167 0.6656029638832649 ap 0 c Lavender

81 181 0.40040032189249264 ap 0 c Blue

19 25 1.0 ap 0 c Lavender

23 172 0.6712630446412372 ap 0 c Blue

219 69 0.6260750747461369 ap 0 c Blue

27 28 0.4652042521107459 ap 0 c Lavender

124 56 1.0 ap 0 c Lavender l TF-DNA

51 217 0.321858932256685 ap 0 c Blue

3 88 1.0 ap 0 c Blue l TF-DNA

80 180 0.30065973649117494 ap 0 c Lavender

56 36 1.0 ap 0 c Lavender l TF-DNA

108 34 0.15555070951709518 ap 0 c Blue

115 56 0.39505959444811334 ap 0 c Lavender

219 70 0.2389055568990938 ap 0 c Lavender

118 37 1.0 ap 0 c Lavender l TF-DNA

32 28 0.4738106606672744 ap 0 c Lavender

96 87 0.3256528871564077 ap 0 c Black

159 161 1.0 ap 0 c Blue

32 27 0.3926874934411427 ap 0 c Lavender

108 172 0.45179642944708737 ap 0 c Blue

57 222 0.6660336872645332 ap 0 c Blue

163 183 1.0 ap 0 c Black l TF-DNA

196 169 0.6349804203372583 ap 0 c Lavender

3 85 1.0 ap 0 c Blue l TF-DNA

127 163 1.0 ap 0 c Black l kinase_substrate

13 250 0.6295970447700182 ap 0 c Lavender

217 163 0.17525542809669736 ap 0 c Black

268 69 0.6506071325890367 ap 0 c Black

75 156 0.48013602704804886 ap 0 c Lavender

163 49 1.0 ap 0 c Black l TF-DNA

132 22 0.2938630770459048 ap 0 c Lavender

142 30 1.0 ap 0 c Blue

237 268 0.42709386671549676 ap 0 c Blue

77 234 0.6526968860041908 ap 0 c Blue

233 163 0.6441021104625074 ap 0 c Blue

20 163 0.6331586693420733 ap 0 c Lavender

50 127 0.48390084890662927 ap 0 c Lavender

32 142 1.0 ap 0 c Lavender l TF-DNA

27 163 1.0 ap 0 c Blue l TF-DNA

172 65 0.46415513877658937 ap 0 c Black

86 85 0.27730029738843576 ap 0 c Lavender

106 123 0.5947570260762174 ap 0 c Lavender

165 106 0.6000898986950852 ap 0 c Lavender

26 113 0.45843544270982556 ap 0 c Lavender

268 100 0.32422337387848726 ap 0 c Black

55 54 0.2381277587718034 ap 0 c Black

190 56 0.4311699886375525 ap 0 c Black

26 163 0.4117892269481872 ap 0 c Lavender

96 172 0.6169477686643221 ap 0 c Black

28 19 1.0 ap 0 c Lavender l TF-DNA

129 157 0.5702443791350399 ap 0 c Lavender

103 206 0.6570995921483023 ap 0 c Lavender

134 157 0.20520734666979773 ap 0 c Lavender

97 59 0.45463781922717805 ap 0 c Blue

185 16 0.5397215786220453 ap 0 c Blue

18 172 0.6564136707035352 ap 0 c Blue

185 115 0.6565326110186523 ap 0 c Lavender

18 90 1.0 ap 0 c Blue

17 166 0.5142324271844838 ap 0 c Black

267 81 0.5441838786524129 ap 0 c Lavender

223 154 0.3091458718489178 ap 0 c Lavender

116 145 0.6685933656663615 ap 0 c Black

115 102 0.40649221556106097 ap 0 c Blue

109 72 1.0 ap 0 c Blue l TF-DNA

177 105 0.6020502501014634 ap 0 c Blue

185 17 0.5599753548903221 ap 0 c Blue

69 219 0.6260750747461369 ap 0 c Black

207 69 0.39532706658130257 ap 0 c Lavender

185 166 0.4508464427741266 ap 0 c Lavender

163 14 1.0 ap 0 c Black l TF-DNA

83 50 0.25860718525276394 ap 0 c Lavender

182 17 1.0 ap 0 c Blue

103 267 0.615377871756672 ap 0 c Lavender

18 92 1.0 ap 0 c Blue

228 215 0.6016102937555475 ap 0 c Lavender

27 25 1.0 ap 0 c Lavender l TF-DNA

269 103 0.26245123353253424 ap 0 c Lavender

109 73 1.0 ap 0 c Blue l TF-DNA

22 36 0.29341961340926564 ap 0 c Lavender

241 16 0.5289412432354668 ap 0 c Lavender

164 163 0.545375011554996 ap 0 c Blue

154 163 0.6285344501550918 ap 0 c Black

167 246 0.6656029638832649 ap 0 c Lavender

115 75 1.0 ap 0 c Lavender l TF-DNA

114 32 0.399843204766859 ap 0 c Lavender

111 103 0.15633008804862547 ap 0 c Lavender

27 82 1.0 ap 0 c Blue l TF-DNA

39 167 0.668500891921627 ap 0 c Lavender

18 87 1.0 ap 0 c Lavender

101 77 0.6073182918731387 ap 0 c Lavender

219 196 0.5829815111301031 ap 0 c Lavender

163 117 0.5174651237673246 ap 0 c Black

163 116 0.6510069773088731 ap 0 c Black

97 90 0.39080799799474836 ap 0 c Lavender

94 115 0.6358310041016854 ap 0 c Lavender

150 235 0.6488369437046476 ap 0 c Lavender

74 72 0.664388272121303 ap 0 c Lavender

70 32 0.24714132713367798 ap 0 c Lavender

115 159 1.0 ap 0 c Lavender l TF-DNA

56 68 0.1545039519628401 ap 0 c Lavender

111 56 0.13258597642416625 ap 0 c Lavender

33 172 0.2844500934331095 ap 0 c Blue

98 4 0.29033010754979977 ap 0 c Lavender

54 121 0.5236958195979478 ap 0 c Lavender

32 111 0.1539229934592591 ap 0 c Lavender

163 119 1.0 ap 0 c Black l TF-DNA

181 166 0.5551252265409474 ap 0 c Lavender

221 110 0.3520205550205446 ap 0 c Lavender

130 26 0.34673184528070666 ap 0 c Lavender

43 26 1.0 ap 0 c Lavender l TF-DNA

103 26 1.0 ap 0 c Lavender l TF-DNA

147 18 0.3868365918960931 ap 0 c Black

22 132 0.2938630770459048 ap 0 c Lavender

103 25 1.0 ap 0 c Lavender l TF-DNA

50 62 1.0 ap 0 c Lavender l TF-DNA

2 67 0.5933229164881911 ap 0 c Blue

32 76 0.3205454476663399 ap 0 c Lavender

115 163 1.0 ap 0 c Blue l TF-DNA

16 10 0.6487923433000795 ap 0 c Blue

10 8 0.4703063612892095 ap 0 c Lavender

20 56 0.3744360107810151 ap 0 c Lavender

27 19 1.0 ap 0 c Lavender l TF-DNA

159 180 0.15015862518025225 ap 0 c Lavender

261 70 0.22518943995382226 ap 0 c Lavender

105 70 0.31305106251766535 ap 0 c Black

115 13 1.0 ap 0 c Lavender l TF-DNA

188 140 0.46459061949426445 ap 0 c Lavender

166 180 0.2244500878594139 ap 0 c Lavender

55 171 0.301486935994481 ap 0 c Black

111 163 0.14436105540750013 ap 0 c Blue

86 184 0.26793645517040154 ap 0 c Lavender

265 126 0.6315932447925552 ap 0 c Blue

8 123 0.45183382828324836 ap 0 c Lavender

255 163 0.5035310834413266 ap 0 c Blue

169 196 0.6349804203372583 ap 0 c Lavender

83 122 0.14747466305657914 ap 0 c Lavender

46 190 0.2519395226442197 ap 0 c Blue

163 55 0.3171869077960314 ap 0 c Black

3 109 1.0 ap 0 c Lavender l TF-DNA

186 126 0.6416984190681595 ap 0 c Lavender

154 151 0.4608697672731732 ap 0 c Black

137 211 0.4533707651700622 ap 0 c Lavender

147 73 0.21542483640198576 ap 0 c Black

181 161 0.4297287999608643 ap 0 c Blue

154 153 0.6408741039707171 ap 0 c Black

154 152 0.6586379338413391 ap 0 c Black

239 136 0.22664008137896272 ap 0 c Black

215 268 0.6242863318701964 ap 0 c Blue

55 163 0.3171869077960314 ap 0 c Black

118 157 1.0 ap 0 c Lavender l TF-DNA

163 53 0.6616150487831145 ap 0 c Black

117 109 0.44642315790407594 ap 0 c Black

12 8 0.3573563714281889 ap 0 c Lavender

189 87 0.5675658451154768 ap 0 c Lavender

4 115 0.4374175514297528 ap 0 c Lavender

95 85 1.0 ap 0 c Lavender l TF-DNA

121 54 0.5236958195979478 ap 0 c Lavender

259 130 0.22791099084736105 ap 0 c Blue

122 83 0.14747466305657914 ap 0 c Lavender

116 37 1.0 ap 0 c Black l TF-DNA

137 81 0.38930547543812716 ap 0 c Blue

163 56 1.0 ap 0 c Black l Both

161 169 0.47233330604145624 ap 0 c Lavender

160 180 0.20386964523350545 ap 0 c Lavender

43 191 1.0 ap 0 c Lavender l TF-DNA

166 181 0.5551252265409474 ap 0 c Lavender

190 46 0.2519395226442197 ap 0 c Black

154 223 0.3091458718489178 ap 0 c Black

198 257 0.40954354149966365 ap 0 c Black

16 100 0.3191177498796565 ap 0 c Lavender

89 126 0.2481138515672485 ap 0 c Blue

110 221 0.3520205550205446 ap 0 c Lavender

97 87 0.385761611612577 ap 0 c Lavender

199 42 1.0 ap 0 c Blue

68 56 0.1545039519628401 ap 0 c Lavender

223 137 0.2887322884652811 ap 0 c Lavender

117 37 1.0 ap 0 c Black l TF-DNA

163 255 0.5035310834413266 ap 0 c Black

203 163 0.3280349149315121 ap 0 c Lavender

263 32 1.0 ap 0 c Lavender

154 155 0.3557731453975958 ap 0 c Black

115 82 1.0 ap 0 c Lavender l TF-DNA

43 57 1.0 ap 0 c Lavender l TF-DNA

81 137 0.38930547543812716 ap 0 c Blue

43 175 1.0 ap 0 c Lavender l TF-DNA

27 58 1.0 ap 0 c Blue l TF-DNA

126 163 0.6656638590792652 ap 0 c Blue

# References

1. Maglott D, Ostell J, Pruitt KD, Tatusova T (2007) Entrez Gene: gene-centered information at NCBI. Nucleic Acids Res 35: D26-31.

2. Bruford EA, Lush MJ, Wright MW, Sneddon TP, Povey S, et al. (2008) The HGNC Database in 2008: a resource for the human genome. Nucleic Acids Res 36: D445-448.

3. Kanehisa M, Araki M, Goto S, Hattori M, Hirakawa M, et al. (2008) KEGG for linking genomes to life and the environment. Nucleic Acids Res 36: D480-484.

4. Ideker T, Ozier O, Schwikowski B, Siegel AF (2002) Discovering regulatory and signalling circuits in molecular interaction networks. Bioinformatics 18 Suppl 1: S233-240.

5. Batagelj V, Mrvar A (1998) Pajek: program for large network analysis. Connections 21: 47-57.
